# The assessment of left ventricular diastolic function: guidance and recommendations from the British Society of Echocardiography

**DOI:** 10.1186/s44156-024-00051-2

**Published:** 2024-06-03

**Authors:** Shaun Robinson, Liam Ring, David Oxborough, Allan Harkness, Sadie Bennett, Bushra Rana, Nilesh Sutaria, Francesco Lo Giudice, Matthew Shun-Shin, Maria Paton, Rae Duncan, James Willis, Claire Colebourn, Gemma Bassindale, Kate Gatenby, Mark Belham, Graham Cole, Daniel Augustine, Otto A. Smiseth

**Affiliations:** 1 Imperial College Healthcare NHS Trust, London, UK; 2 West Suffolk Hospital NHS Trust, Bury St Edmunds, UK; 3 Liverpool John Moore’s University, Liverpool, UK; 4 East Suffolk and North Essex NHS Foundation Trust, Colchester, UK; 5 University Hospital of the North Midlands, Stoke-On-Trent, UK; 6 Leeds Teaching Hospitals, Leeds, UK; 7 Newcastle Upon Tyne Hospitals NHS Foundation Trust, Newcastle, UK; 8 Royal United Hospital Bath, Bath, UK; 9 Oxford University Hospitals, Oxford, UK; 10 Addenbrookes Hospital, Cambridge University Hospitals, Cambridge, UK; 11 Division of Cardiovascular and Pulmonary Diseases, Oslo University Hospital, Rikshospitalet and University of Oslo, Oslo, Norway

**Keywords:** Diastolic function, Filling pressures, Left atrial pressure, HFpEF

## Abstract

Impairment of left ventricular (LV) diastolic function is common amongst those with left heart disease and is associated with significant morbidity. Given that, in simple terms, the ventricle can only eject the volume with which it fills and that approximately one half of hospitalisations for heart failure (HF) are in those with normal/’preserved’ left ventricular ejection fraction (HFpEF) (Bianco et al. in JACC Cardiovasc Imaging. 13:258–271, 2020. 10.1016/j.jcmg.2018.12.035), where abnormalities of ventricular filling are the cause of symptoms, it is clear that the assessment of left ventricular diastolic function (LVDF) is crucial for understanding global cardiac function and for identifying the wider effects of disease processes. Invasive methods of measuring LV relaxation and filling pressures are considered the gold-standard for investigating diastolic function. However, the high temporal resolution of trans-thoracic echocardiography (TTE) with widely validated and reproducible measures available at the patient’s bedside and without the need for invasive procedures involving ionising radiation have established echocardiography as the primary imaging modality. The comprehensive assessment of LVDF is therefore a fundamental element of the standard TTE (Robinson et al. in Echo Res Pract7:G59–G93, 2020. 10.1530/ERP-20-0026). However, the echocardiographic assessment of diastolic function is complex. In the broadest and most basic terms, ventricular diastole comprises an early filling phase when blood is drawn, by suction, into the ventricle as it rapidly recoils and lengthens following the preceding systolic contraction and shortening. This is followed in late diastole by distension of the compliant LV when atrial contraction actively contributes to ventricular filling. When LVDF is normal, ventricular filling is achieved at low pressure both at rest and during exertion. However, this basic description merely summarises the complex physiology that enables the diastolic process and defines it according to the mechanical method by which the ventricles fill, overlooking the myocardial function, properties of chamber compliance and pressure differentials that determine the capacity for LV filling. Unlike ventricular systolic function where single parameters are utilised to define myocardial performance (LV ejection fraction (LVEF) and Global Longitudinal Strain (GLS)), the assessment of diastolic function relies on the interpretation of multiple myocardial and blood-flow velocity parameters, along with left atrial (LA) size and function, in order to diagnose the presence and degree of impairment. The echocardiographic assessment of diastolic function is therefore multifaceted and complex, requiring an algorithmic approach that incorporates parameters of myocardial relaxation/recoil, chamber compliance and function under variable loading conditions and the intra-cavity pressures under which these processes occur. This guideline outlines a structured approach to the assessment of diastolic function and includes recommendations for the assessment of LV relaxation and filling pressures. Non-routine echocardiographic measures are described alongside guidance for application in specific circumstances. Provocative methods for revealing increased filling pressure on exertion are described and novel and emerging modalities considered. For rapid access to the core recommendations of the diastolic guideline, a quick-reference guide (additional file 1) accompanies the main guideline document. This describes in very brief detail the diastolic investigation in each patient group and includes all algorithms and core reference tables.

**Supplementary Information:**

The online version contains supplementary material available at 10.1186/s44156-024-00051-2.

## Ventricular anatomy, physiology and mechanics

### Myocardial architecture and function

To appreciate the myocardial mechanics that enable global LV contraction and relaxation, it is important to understand the composition of the LV myocardium that enables these processes.

#### Myocyte alignment defining myocardial layers

It is the shortening and lengthening of cardiac myocytes along planes of alignment that enables the ventricular cavity to decrease and increase volume, producing systolic ejection and diastolic filling. The bulk of the ventricular myocardium is composed of contractile myocytes that branch at each end to form connections with adjacent myocytes [3]. This branch-connectivity creates an interconnected network of cardiomyocytes that forms the basis of the multi-layered architecture of the ventricular myocardium, enabling the complex processes of ventricular contraction and relaxation. When considered according to myocyte alignment and orientation, the LV myocardium consists of three layers, albeit without distinct borders between them: the sub-epicardium, mid-wall and sub-endocardium. Sub-epicardial fibres are orientated in a left-handed (LH) helical arrangement and account for around 25% of the total myocardial wall thickness [4]. Aligned obliquely longitudinal, they extend from the level of the atrioventricular valves at the base of the ventricles. When viewed from an anterior perspective, the sub-epicardial fibres run down obliquely leftward and continue to the apex; the fibres originating from the base of the left ventricle extend towards the diaphragmatic surface of the heart, crossing the posterior interventricular groove [3–5]. Contraction of this layer is largely responsible for torsion of the apex relative to the base [6]. Fibres in the mid-wall account for around 53–59% of the myocardial thickness, increasing in the elderly [7], and are arranged circumferentially and in near parallel alignment with the mitral valve (MV) annulus; these fibres largely generate radial contraction [6]. The sub-endocardial layer is the thinnest layer, accounting for < 20% of the total myocardial thickness. These fibres are arranged in a right-handed (RH) helix and obliquely longitudinal pattern, generating longitudinal and rotational contraction of this layer [8]. Fundamentally, the LV consists of two muscular helixes that surround the midventricular circumferential layer of muscular fibres.

**Fig. 1:**
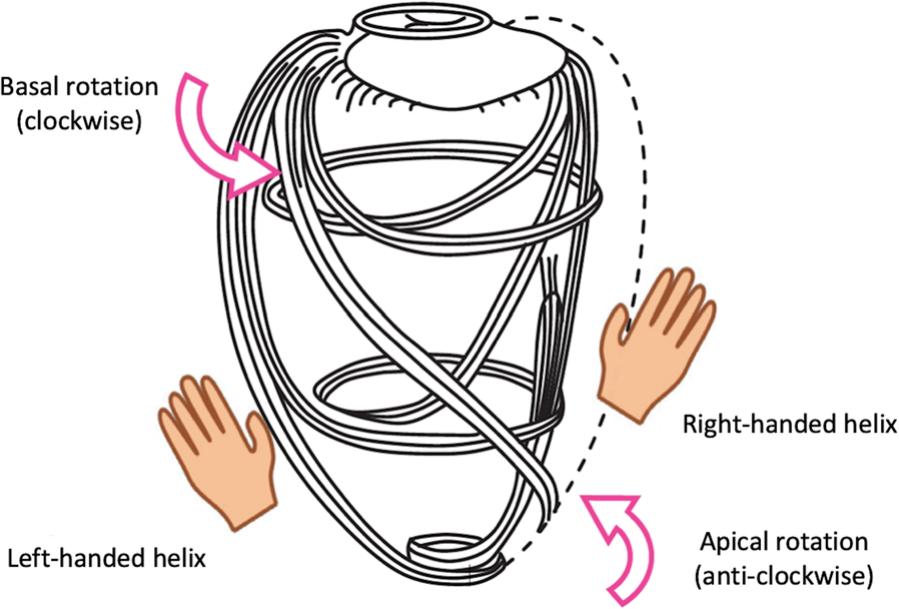
Helical arrangement of myocardial fibres—Nakatani, 2011 [8]

#### Myocardial mechanics and ventricular contraction

This complex configuration of myocyte alignment (sub-epicardial—LH helix, sub-endocardial—RH helix, mid-wall—circumferential) enables three-dimensional contraction that results in rotation, shortening and inward contraction of the myocardium with the net effect of reducing LV cavity dimensions in all planes.

**Fig. 2:**
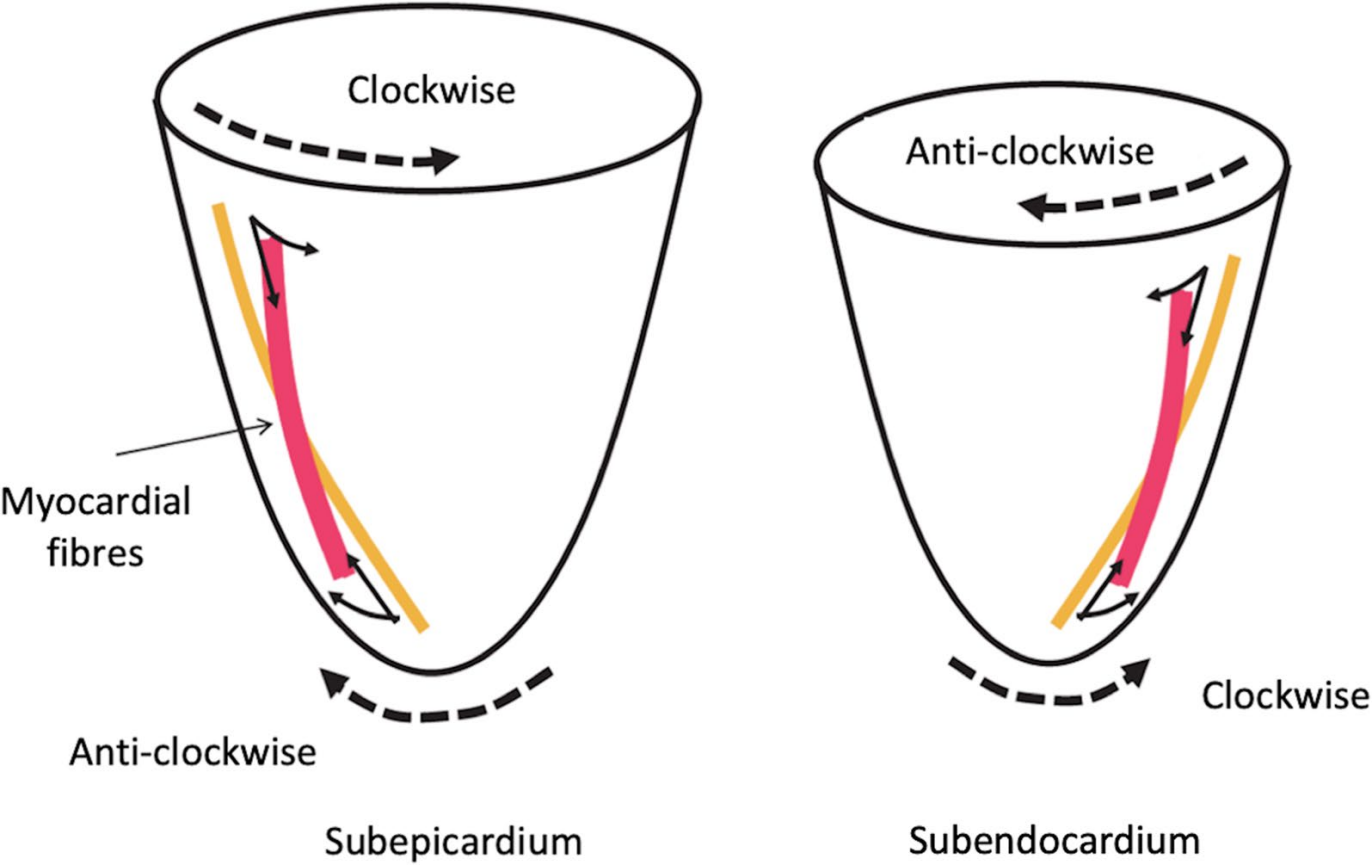
Basal and apical rotational direction of myocardial contraction—Nakatani, 2011 [8]

**Fig. 3:**
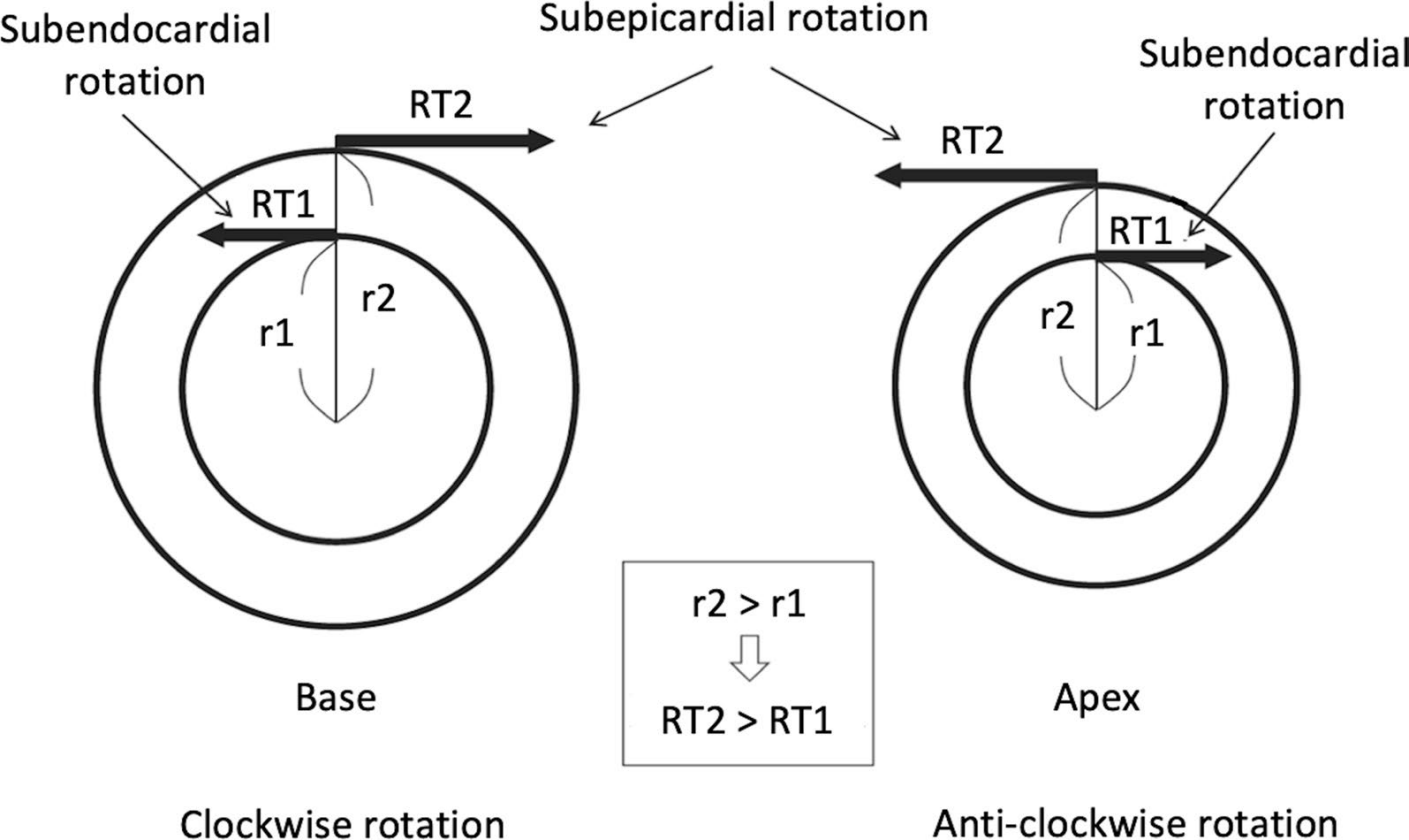
Subendocardial and subepicardial rotational directions at the base and apex—from Nakatani, 2011 [8]

Contraction of sub-epicardial fibres, arranged in a LH helix, causes anti-clockwise rotation of the apical sub-epicardium and clockwise rotation of the base, while contraction of the sub-endocardial fibres, arranged in a RH helix, causes clockwise rotation of the apical sub-endocardium and anticlockwise rotation at the base (Fig. 1) [8, 9]. However, because the rotational radius of the outer layer is greater than that of the inner layer and therefore produces greater torque, the sub-epicardial direction of contraction dominates when both layers contract simultaneously [9]. Consequently, global apical rotation is clockwise very briefly during isovolumetric contraction before reversing and rotating anti-clockwise during the ejection phase (Fig. 2). Global basal contraction mirrors this process, rotating anti-clockwise very briefly during isovolumetric contraction before reversing to clockwise rotation during systolic ejection. Therefore, global LV systolic rotation is predominantly anti-clockwise at the apex and clockwise at the base (Fig. 3).

From a mechanistic perspective, twisting of the myocardium along these planes helps maintain a uniform myocardial fibre stress and shortening that produces a high global contraction percentage (LVEF ~ 60%) from relatively small myocyte shortening (~ 20%) [10–12]. Importantly for the efficiency of diastolic filling, twisting and deformation of the myocardial matrix throughout systole causes a progressive build-up of potential energy [13]. This energy is released following peak contraction resulting in rapid recoil of the circumferential fibres and untwisting of the sub-epi and sub-endocardial helices during isovolumic relaxation and the early period of diastolic filling. The clockwise untwisting of the apex and simultaneous anti-clockwise untwisting of the base generates rapid relaxation of the LV cavity and consequently early diastolic suction [14–16].

Due to the tomographic nature of routine 2D TTE, this complex three-dimensional contraction is viewed, and therefore measured, in 2D orthogonal planes: mitral annular Tissue-Doppler Imaging (TDI) and GLS measure longitudinal lengthening/shortening of the LV, while LVEF predominantly measures radial contractility with some minor contribution from longitudinal shortening. Therefore, because contraction and relaxation of the cardiomyocytes is not purely longitudinal or radial in direction, 2D indices of deformation in these planes are not precisely reflective of LV myocardial contraction. Nonetheless, these echocardiographic measures of longitudinal and radial contraction are well validated and provide important diagnostic and prognostic insight into disease processes and their effect on myocardial function.

#### Normal left ventricular function throughout the cardiac cycle

The LV cardiac cycle is broadly divided into two phases: systole and diastole. Although these phases are often considered independently when being investigated by echocardiography, the proficiency of the ventricle to fill and eject reflects global myocardial performance. Given that the extent of systolic contraction and ejection must be equal to that of diastolic relaxation and filling (as the ventricle cannot perpetually eject more volume than has entered the chamber during the preceding filling period, and vice-versa), there exists a crucial interdependence between LV systolic and diastolic function. Consequently, both phases are simultaneously susceptible to deterioration secondary to disease processes and although dysfunction of one phase may be the predominate cause of symptoms, it is highly likely that this will be accompanied by some degree of impairment of the other. Therefore, in order to fully understand the physiological mechanisms that govern the timings and pressure differentials that enable ventricular filling, and to appreciate the importance of systolic/diastolic interdependence, it is essential to understand ventricular function throughout the cardiac cycle and how this determines filling, filling pressure (LVFP) and ejection.

### Left ventricular systole

LV systole is defined as the period between MV closure and aortic valve (AV) closure and consists of two phases that are termed according to the changes of LV volume: isovolumetric contraction and the ejection phase.

#### Isovolumetric contraction

**Fig. 4:**
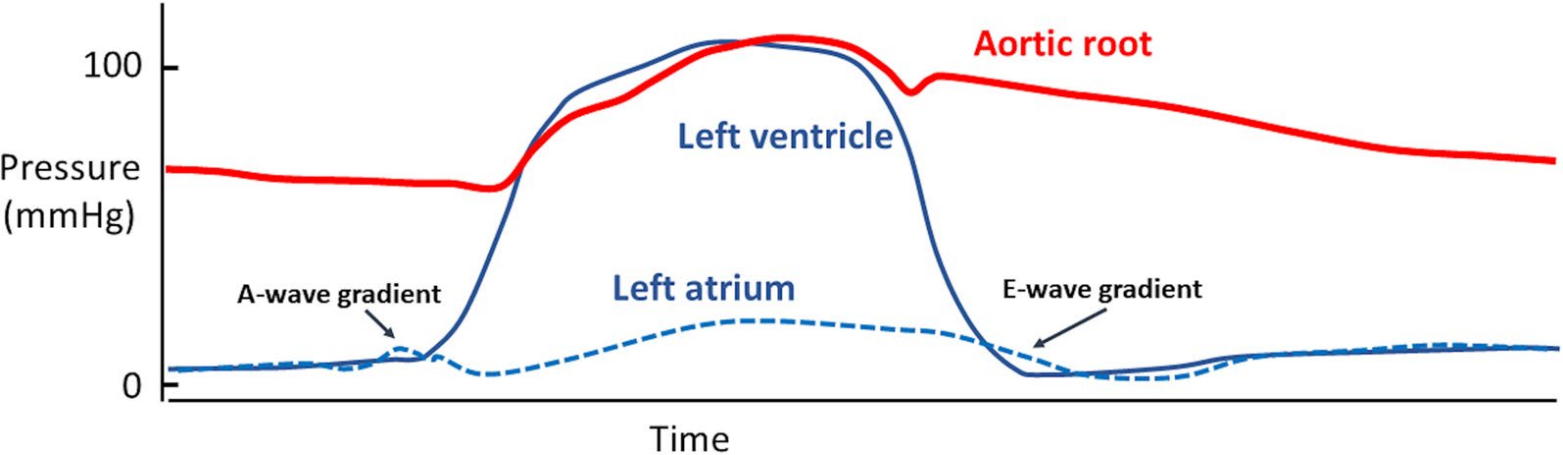
Recording of simultaneous LV and Ao pressures (the representation of LAP has been added)—O.Smiseth’s own work

LV diastole and filling end when LA contraction is complete and LA-LV pressures are close to equal, leading to the start of MV closure (Fig. 4) [17]. With the onset of systolic contraction, LV cavity pressure increases above LA pressure (LAP) and the MV closes. Although LV pressure is increasing and exceeds LAP, it remains below the pressure within the aorta and the AV remains closed [18]. Therefore, because the ventricle is within the contractile phase but internal pressure prevents blood from entering or leaving the chamber, this period is referred to as the isovolumetric contraction time (IVCT).

#### Systolic ejection

Once LV pressure exceeds aortic pressure, the AV is thrust open and blood is ejected from the LV; this period is referred to as the ejection phase. Although peak systolic contraction and deformation is achieved in late systole, peak LV pressure, and consequently outflow velocities, peak in mid systole before falling as the ventricular volume decreases towards end-systole. The AV closes following peak LV contraction when the cavity pressure falls below the pressure in the aorta, thus defining the end of the systolic period.

### Left ventricular diastole

Diastole is defined as the period between AV closure and MV closure and includes periods of isovolumetric relaxation and ventricular filling. In sinus rhythm (SR) with normal heart rate (HR) and no conduction delay between the atria and ventricles, diastole is a four-phase process that comprises periods of: isovolumetric relaxation, early rapid filling, a period of little or no filling (diastasis) and atrial contraction (Fig. 4).

#### Isovolumetric relaxation

Throughout systole, compression and torsion of the myocardium generates a progressive build-up of potential energy within the elastic elements of the cardiomyocytes and extracellular matrix [19], peaking at end-systole. This energy is then released in early diastole as the twisted and compressed cardiomyocytes recoil and relax back to their unstressed/resting orientation, resulting in rapid recoil and untwist of the LV. Combined with a contribution from active myocyte relaxation, this leads to a rapid increase in LV cavity dimensions that causes an equally rapid fall in intracavity pressure. This near constant rate of LV relaxation causes a near exponential rate of pressure decay that can be measured by the time constant, Tau (τ). Since the rate at which intracavity pressure falls is determined by τ, the rate of pressure-decay can be measured as an indicator of LV relaxation and therefore diastolic myocardial function [20].

**Fig. 5:**
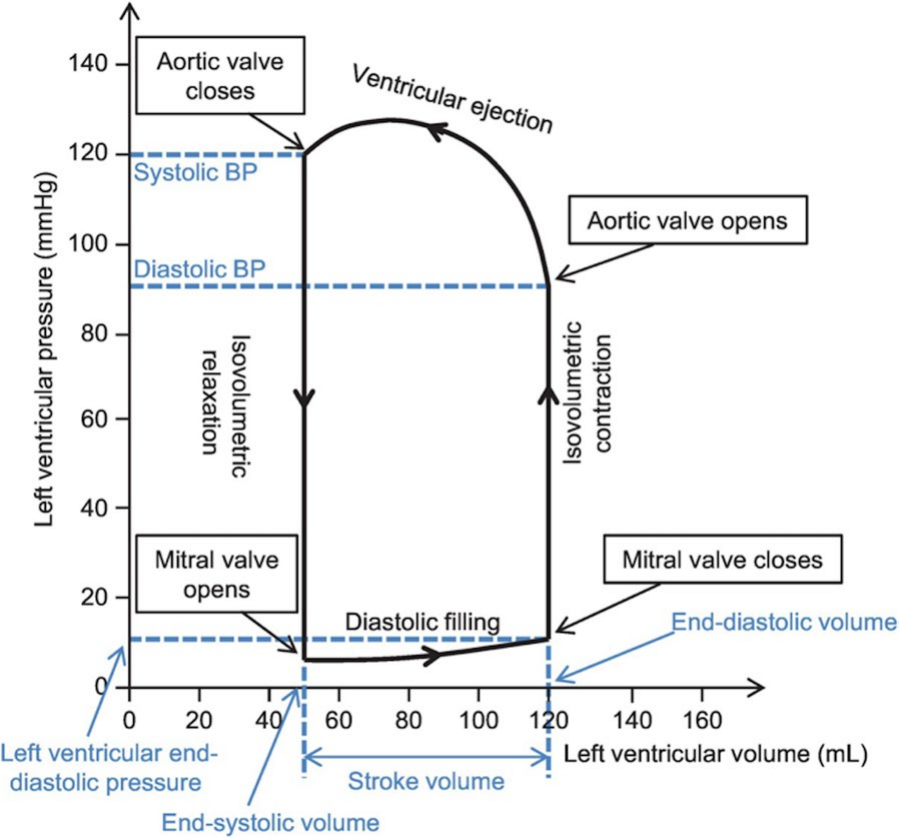
Pressure–volume loop demonstrating changes in ventricular volume during filling and ejection with corresponding changes in intracavity pressure. The isovolumetric relaxation and contraction periods are highlighted [21]

The onset of diastole is defined mechanically by closure of the AV. LV pressure at the start of diastole is therefore high and just below aortic pressure (Fig. 5). For a short period following AV closure, despite falling, pressure within the rapidly relaxing LV continues to exceed LAP and the MV remains shut with consequently no ventricular filling [21]. Given that both the AV and MV are shut and LV volume is unchanged from the point of AV closure, this phase is described as the Isovolumetric Relaxation Time (IVRT) (Fig. 5) and lasts between AV closure and MV opening. In both normal and disease states, the rate of relaxation is constant with a near linear relationship with τ; because a normal τ is typically less than 45 ms in most age groups, the IVRT is short in those with normal diastolic function [22].

#### Early filling

**Fig. 6:**
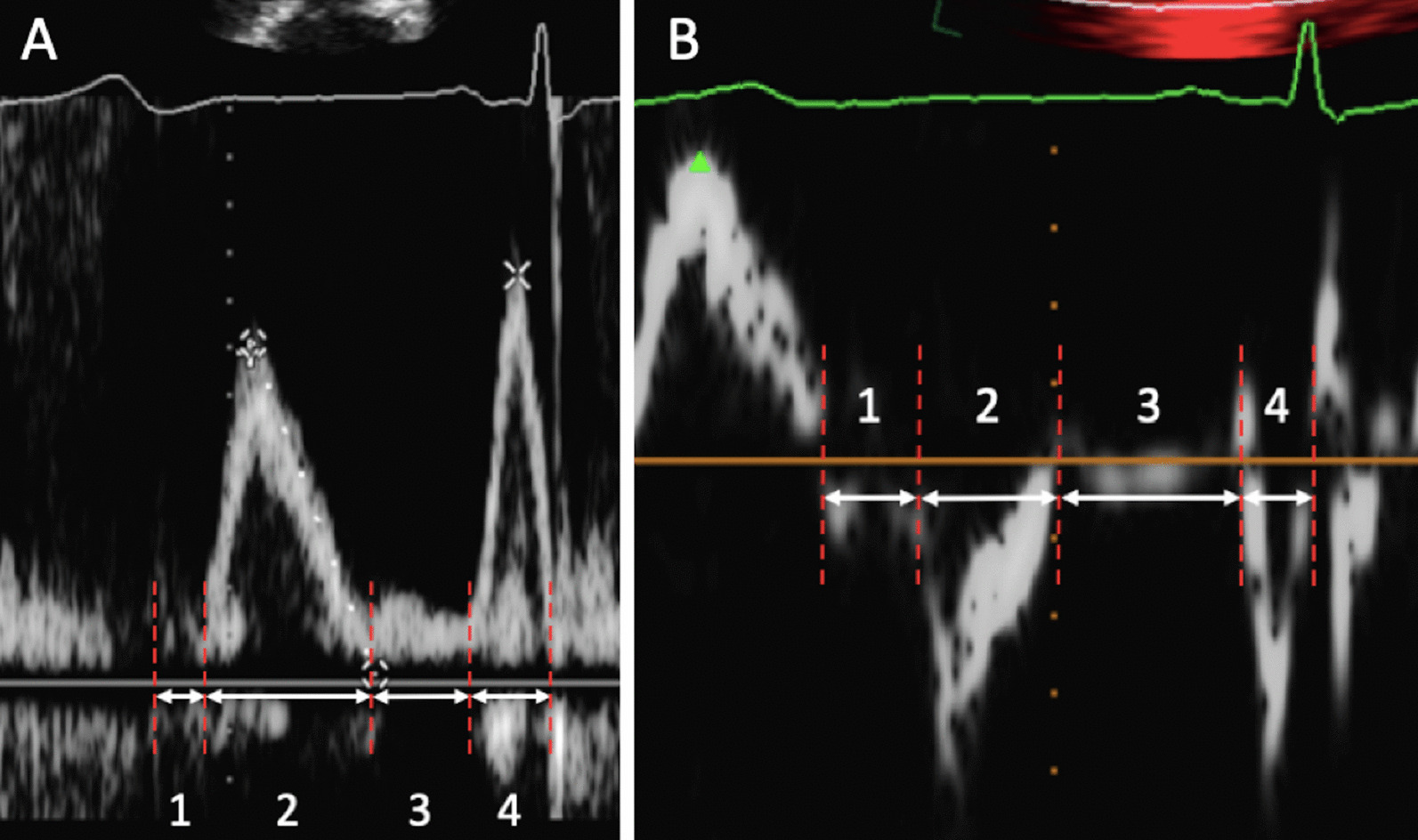
The four phases of diastole shown on a spectral Doppler trace of mitral inflow (**A**) and mitral annular tissue-Doppler imaging (**B**)—(1) IVRT, (2) early filling, (3) diastasis, (4) late filling from atrial contraction

Continued untwisting and relaxation of the LV causes intracavity pressure to fall. Once pressure in the LV falls below pressure in the LA, suction effect causes the MV to open and blood flows from the LA into the LV, marking the onset of the rapid early filling phase and the end of the IVRT (Fig. 6). For a very brief period of 30**–**40 ms following MV opening, the rapid rate of LV relaxation is such that pressure within the LV continues to fall despite the initial increase in volume, creating a pressure gradient from the LA to LV apex that results in flow accelerating out of the LA [24–26]; minimal LV diastolic pressure is therefore typically reached at around 3.5 × τ [22]. With continued LV relaxation and rapidly increasing LV volume towards its relaxed capacity, LV cavity pressure rises with a progressive reduction in the LA-LV pressure difference and resultant fall in the transmitral flow velocity. In young healthy hearts and at a normal HR, between 80 and 90% of total LV filling occurs during the early diastolic filling phase with the majority of early LV filling completed by 140 ms.

#### Diastasis

As the early filling period ends and the LV reaches its relaxed volume, LV diastolic pressures increase with little to no pressure difference existing between the LA and LV. Consequently, transmitral flow volume and velocity fall significantly and the MV leaflets return to a semi-open or even almost closed position. This period of little to no flow following early passive filling and before atrial contraction is termed diastasis. With a normal P-R interval, the duration of diastasis is determined by the diastolic period and therefore HR, with bradycardia resulting in a longer period between the early filling phase and atrial contraction and therefore longer diastasis.

#### Late filling from atrial contraction

The final phase of ventricular filling occurs when LA contraction increases LA pressure, forcing the MV to open and ejecting blood into the LV. In the setting of normal diastolic function, the relaxed LV offers very little resistance to additional filling and the majority of blood ejected by the LA enters the LV, with only a small proportion being ejected back into the pulmonary veins. During this final stage of diastole, because the LV cardiomyocytes are completely relaxed, highly compliant and distensible, the 10–20% additional volume from atrial contraction is achieved with a < 5 mmHg increase in EDP [27]. This enables the LV to fill at very low pressure with consequently low pressure within the LA, pulmonary veins (PV) and therefore pulmonary capillary bed (Fig. 6).

### Atrial function and pulmonary vein flow

**Fig. 7:**
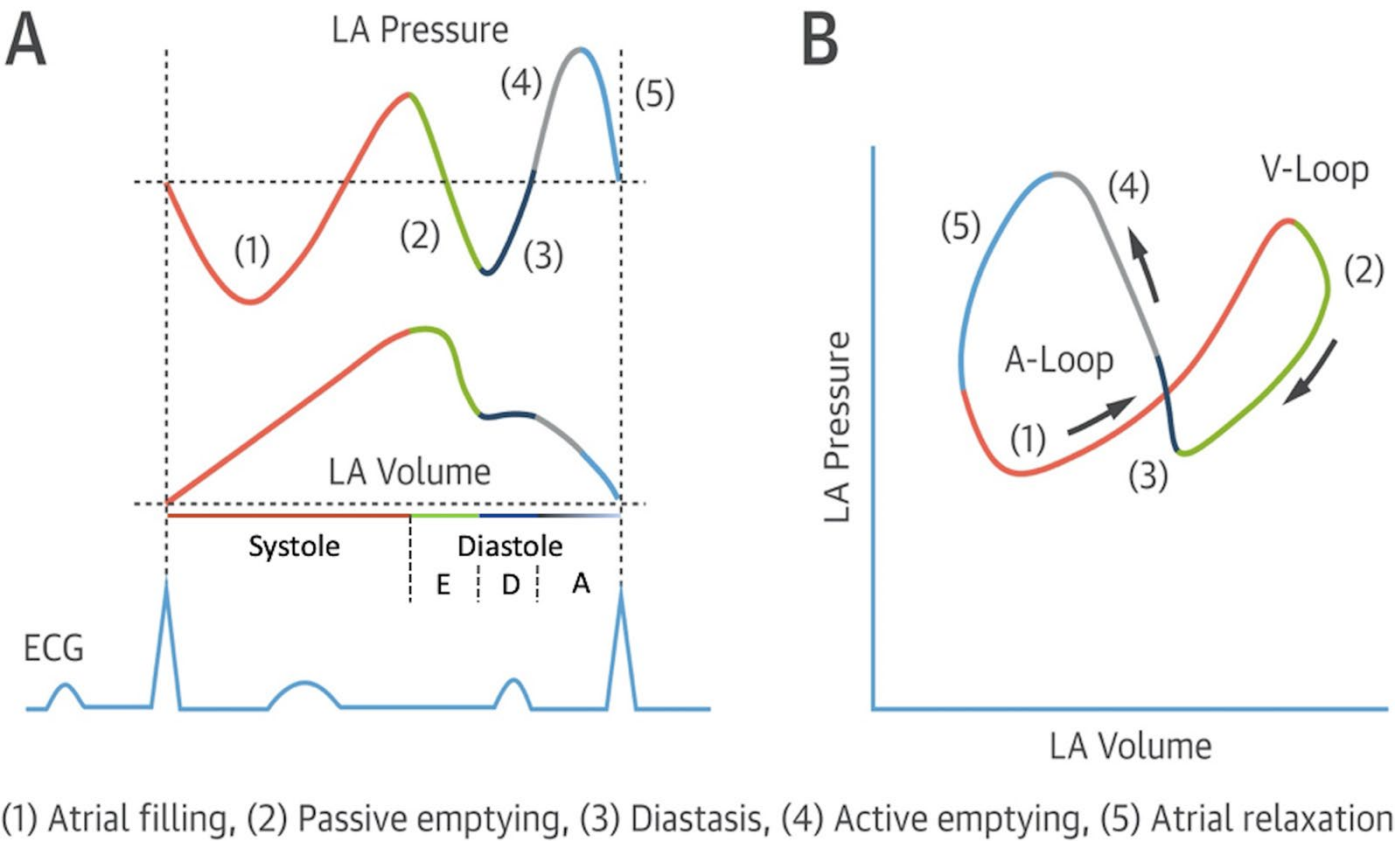
The phases of atrial function demonstrated using pressure volume loop—adapted from Negishi et al. The phases of the cardiac cycle have been highlighted on image A. Diastole has been divided into: E—early filling, D—diastasis and A—atrial contraction. The reservoir phase (red trace—1) occurs during ventricular systole: pulmonary venous blood enters the LA resulting in LA volume increasing from minimum to maximum. In normal circumstances, the associated increase in LA pressure is small, owing to atrial compliance and distensibility. Immediately after mitral valve opening, there is reduction in LA volume and pressure as blood enters the LV during the conduit phase (green trace—2). At a low enough HR there is a period between early and late diastolic filling where LA and LV pressures are close to equal with consequently minimal transmitral flow (dark blue trace—3). Finally, the atrium contracts, marking the onset of the pump phase (grey trace—4). This is accompanied by a rapid increase in LA pressure and blood is ejected from the LA into the LV with some retrograde flow into the PV’s (the A-reversal wave). Immediately after contraction, the LA recoils and relaxation commences (light blue trace—5), leading to the start of the reservoir phase [29]

As a reservoir for blood prior to early LV diastolic filling and through pump contribution in late diastole, LA function modulates LV filling and is therefore an important component of LVDF. In connection with the LA, flow from the PV’s reflects the phases of LA filling and contraction and therefore provides insight into LVDF, LVFP and LAP [28]. In SR, there are three phases of LA function, each of which are identifiable during TTE by alterations in LA chamber dimensions and by blood flow into and out of the LA [29] (Fig. 7).

#### Reservoir phase

The LA reservoir phase occurs during LV systolic contraction with PV flow during this period occurring over two phases. At the very start of LV systole, elastic recoil of the LA immediately after atrial contraction causes dimensions to increase and LAP to fall, drawing blood in from the PV’s and marking the initial phase of PV systolic flow. Ongoing systolic contraction and shortening of the LV leads to descent of the MV annulus towards the LV apex. As the roof of the LA is relatively fixed in position, this motion stretches the LA and increases dimensions from a contracted minimum at end-diastole to a maximum volume at ventricular end-systole (Fig. 7).

**Fig. 8:**
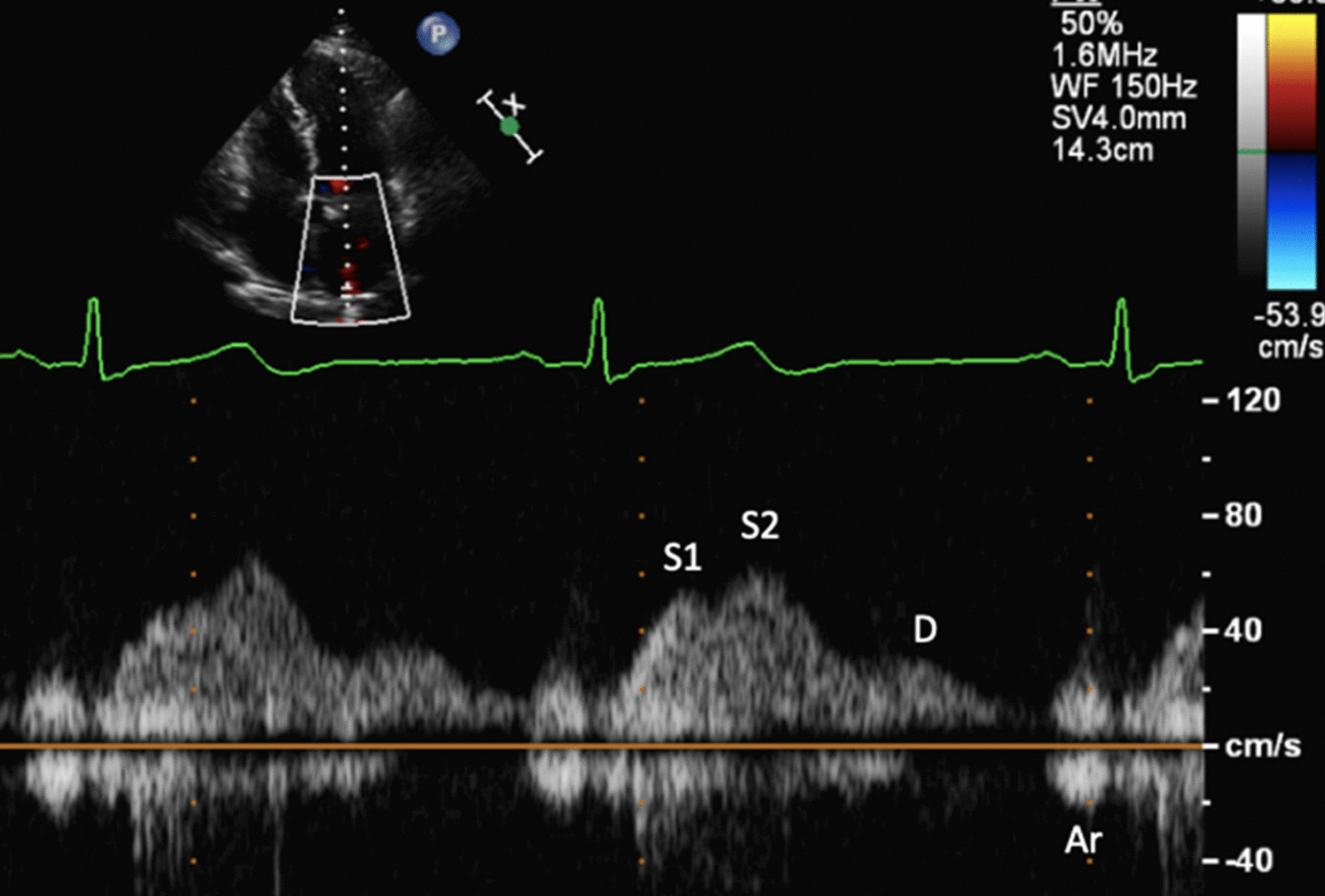
Pulsed Wave Doppler spectral display of pulmonary vein flow. S1 and S2 waves reflect left atrial filling during LV systole (LA reservoir phase). The D wave reflects pulmonary vein flow during early ventricular diastole (LA conduit phase). The Ar wave reflects flow reversal within the pulmonary veins secondary to atrial contraction (LA pump phase)

The increase in LA dimensions causes LAP to fall further while right ventricular (RV) systolic pressure is simultaneously propagated through the pulmonary vasculature. These actions combine to create a pressure differential that drives blood flow through the lungs and PV’s into the LA—noted as the second phase of PV flow [30] (Fig. 8). The LA therefore functions as a ‘reservoir’ of blood during LV systole. As LA filling during this phase relies on a combination of chamber stretch (through LV longitudinal shortening), RV systolic pressure propagated through the lungs [30] and intrinsic LA distensibility, this aspect of LA function is broadly related to LV stroke volume (SV) and atrial compliance [31, 32]. The total LA reservoir volume is ejected into the LV over two phases: passive LA contraction/compression in early diastole (secondary to LV recoil and relaxation) and active contraction (pump) in atrial systole.

#### Conduit phase

The LA conduit phase starts with MV opening and continues until active atrial contraction. However, in atrial fibrillation (AF), the absence of atrial contraction means that this phase continues until end-diastole. The conduit phase is represented by the volume of blood that is transported from the PV’s to the LV without being stored in the atrium and can be estimated by considering changes in LA dimensions in comparison to LV SV, as follows:

In normal circumstances (absence of aortic or mitral regurgitation), the volume of blood ejected by the LV in systole, the SV, is equal to the volume of blood that it fills with in diastole (total filling volume). This total diastolic filling volume (and therefore SV) is achieved by two filling mechanisms that occur simultaneously:

- emptying of the LA from its maximum volume at end-systole to a minimum volume following atrial contraction at end-diastole—*LA reservoir volume*.
- blood drawn into the LV by suction effect, secondary to LV relaxation in early to mid-diastole, that simply passes through the LA from PV’s—*LA conduit volume*.

The contribution of LA reservoir volume to LV filling is calculated as the difference between maximum LA volume and minimum LA volume. Therefore, because the total LV filling volume and SV are equal, the contribution of conduit volume to LV filling is calculated as the difference between LA reservoir volume and SV.

For example:

$${\text{LA}}\;{\text{reservoir}}\;{\text{volume}}\, = \,{\text{maximum}}\;{\text{LA}}\;{\text{volume}}-{\text{minimum}}\;{\text{LA}}\;{\text{volume}}$$

$${\text{LA}}\;{\text{conduit}}\;{\text{volume}}\, = \,{\text{LVSV}}-\;{\text{LA}}\;{\text{reservoir}}\;{\text{volume}}{.}$$

∴

$${\text{LVSV}}\, = \,90\;{\text{mL}}.$$

$${\text{LA}}\;{\text{reservoir}}\;{\text{volume}}\, = \,{\text{LA}}\;\max {\text{volume (60 mL)}}-\;{\text{LA}}\;\min {\text{volume (35 mL)}}{.}$$

$${\text{LA}}\;{\text{conduit}}\;{\text{volume}}\, = \,{\text{LVSV}}\;{\text{(90 mL)}}-\;{\text{LA}}\;{\text{reservoir}}\;{\text{volume (25 mL)}}{.}$$

$${\text{LA}}\;{\text{conduit}}\;{\text{volume}}\, = \,6{\text{5 mL}}{.}$$

Although this calculation does not consider the volume of blood ejected back into the PV’s during the LA pump phase, this volume of blood is insignificant and cannot be measured by echocardiography.

#### Active contraction phase

The final stage of LA function is the pump phase, sometimes described as the contractile phase, when blood is actively ejected into the LV. LA contraction contributes between 10–15% of total filling in the healthy young [32], increasing up to 35–40% in the healthy elderly [33]. When LVEDP is normal, the majority of blood ejected from the LA enters the LV and is identified as the A wave on transmitral Doppler. However, even in normal healthy hearts, LA contraction results in a small volume of blood being ejected backward into the pulmonary veins (Fig. 8). The LA pump phase is an important mechanism by which LV filling can be maximised and SV maintained.

### The spectrum of impaired diastolic function

Although sudden cardiac events may have an immediate adverse effect on LVDF and LVFP, the development of impaired diastolic function is typically a chronic process where deterioration is usually determined by aetiology and the effectiveness of medical management of the underlying disease. Many pathological processes affect myocardial function and consequently alter the properties of left ventricular relaxation and compliance, thus limiting the capacity of LV filling and causing LV and LA diastolic pressures to increase. Although impaired diastolic function broadly describes the complex continuum from normality to restrictive filling, three important physiological aspects define LV filling capability and should be considered by echocardiography: myocardial relaxation, chamber compliance and LVFP [34]. While there is no certainty of progression, impaired diastolic function will initially present in the very early stages as a subclinical abnormality of relaxation with no significant effect on LVFP or associated symptoms. If progressive, it may advance through the spectrum of impairment to restrictive ventricular filling with the significant haemodynamic consequences of markedly raised LVFP and pulmonary hypertension (PH) that cause the symptoms of HF.

#### Impaired relaxation, normal LV compliance

The earliest stage of diastolic impairment is characterised by impaired relaxation but with normal chamber compliance. Impaired relaxation is defined by a longer τ and reduced relaxation velocities [20]. Given that τ is linearly related to the rate of pressure fall within the LV, a slower rate of LV relaxation results in a slower rate of pressure fall and consequently longer time period between AV closure at the very start of diastole and MV opening with the onset of LV filling. Impaired LV relaxation is therefore reflected by a longer IVRT. Following the extended IVRT, the MV opens and the LV begins to fill. However, because the early diastolic filling rate is proportional to the rate of pressure decay, which in turn is determined by the rate of relaxation (τ), the early diastolic ventricular filling rate, and therefore volume and velocity, is reduced when relaxation is impaired [35]. In essence, for the same filling period, impaired relaxation reduces the early diastolic filling rate and therefore volume with a greater proportion of total filling, approaching 35–40%, occurring through atrial contraction in late diastole in order to maintain SV and cardiac output (CO).

**Fig. 9:**
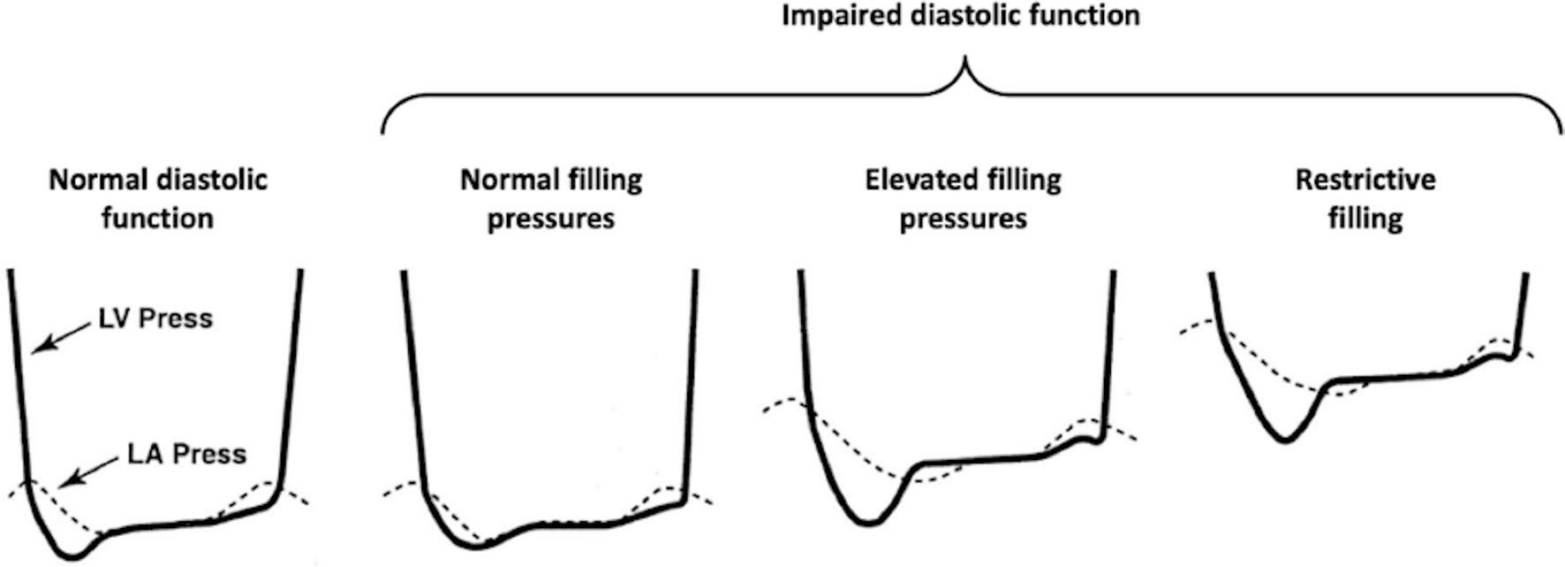
Relative changes in LA and LV diastolic pressures in different stages of impaired diastolic function. LAP remains normal when relaxation is impaired but becomes elevated when LV compliance is decreased. [42] Adapted from Panesar, Dilveer and Burch

Despite relaxation being impaired the LV remains compliant, meaning that the chamber is able to distend/stretch to accommodate the filling volume and maintain low LVFP. Minimum LV diastolic pressure is closely related to the relaxation properties of the LV and therefore occurs very early in diastole [36]. With normal relaxation and filling, the minimum filling pressure is low—in the young and athletic, low minimum pressure increases the transmitral pressure difference while LAP remains normal, creating a suction effect that facilitates rapid early filling at normal filling pressure. However, as relaxation becomes impaired and the rate of pressure decay falls, minimum LV diastolic pressure increases and reduces the early diastolic pressure difference, therefore attenuating the suction force for flow between the LA and LV [37] and reducing early diastolic transmitral velocities. Although impaired LV relaxation leads to increased minimum pressure, because the LV remains compliant and is able to distend to accommodate atrial pump volume, LVFP, and therefore LAP, remain normal (Fig. 9). Importantly, impaired relaxation may cause a mild increase in LV EDP that although not contributory to symptoms, is an early indicator of impaired diastolic function [38].

#### Impaired relaxation, reduced LV compliance

Worsening diastolic function is characterised by continued deterioration of LV relaxation rate and velocities with increasing myocardial stiffness and reduced chamber compliance. Increasing LV stiffness and consequently reduced LV compliance leads to increased LV diastolic pressure even at normal filling volume. As the myocardium becomes stiffer and LV compliance deteriorates further, LV filling becomes restrictive with significantly elevated LV diastolic pressure, and therefore LAP, for even low filling volumes (Fig. 9).

Elevation of LV diastolic pressures and LAP has a significant impact on all phases of ventricular filling. Whereas impaired relaxation with normal LAP results in a longer IVRT, elevated LAP causes the MV to open sooner and therefore shortens the IVRT. Although relaxation is impaired with marked attenuation of the diastolic suction effect, filling in early diastole is now dominated by elevated LAP. Therefore, upon MV opening, high LAP causes high transmitral flow velocity in early diastole. Due to reduced LV compliance, LV pressure rises rapidly with rapid equalisation between LA-LV pressures and an equally rapid deceleration of early diastolic transmitral flow velocity—the early diastolic filling period is therefore typically shortened when LV diastolic pressure and LAP are elevated. Following early diastolic filling, flow between the LA and LV during diastasis is typically low volume and low velocity when LAP is normal. However, LA incompliance due to raised LAP (secondary to LV impaired diastolic function) may result in continued flow between the LA and LV during this period (as flow from the PV’s into a stiff LA continues during diastasis, LAP increases resulting in transmitral forward flow (L-wave)) [39]. With reduced LV compliance causing elevated diastolic pressure, LA contraction at end-diastole is against a higher resistance (afterload) and causes further elevation of LV EDP. Increased resistance to LA contraction reduces the transmitral forward flow volume, velocity and duration and results in a greater proportion of blood being ejected back into the PV’s and for a longer duration than forward flow [39]. When LVEDP is significantly raised, LA pump function deteriorates in the face of significantly increased afterload. Consequently, because the LV, LA and PV’s are in continuity with the pulmonary capillary bed, increased LV diastolic pressure is transmitted back through the LA and PV’s leading to increased pulmonary capillary pressure and consequently PH [40, 41].

#### Effect of normal aging on LV filling

The effect of normal aging on LV filling mimics the early stages of impaired diastolic function such that a slower rate of myocardial relaxation and lower relaxation velocities are expected findings in the elderly. Therefore, an extended IVRT, reduced early diastolic filling and increased late diastolic filling are normal findings. Mean LAP does not increase with aging, although LV EDP may become mildly elevated but is not typically associated with symptoms [43]. Although these findings are similar to the initial stages of impaired LV diastolic function, they should be expected in those over the age of 65 years. In fact, a short IVRT and predominance of early diastolic filling in the elderly should raise the suspicion of impaired LV diastolic function with raised LVFP.

## Echocardiographic measurements: routine, supplementary and non-routine measures

Given the multiple aspects of diastolic mechanics and associated LVFP, there is no single parameter or measure that accurately describes ventricular diastolic function and the response of intracavity pressure. The echocardiographic assessment of LV diastole must therefore incorporate multiple parameters of intracardiac blood flow velocity, myocardial relaxation velocity and left atrial size and function in order to consider the presence and degree of diastolic impairment. Optimisation of all two-dimensional images and Doppler waveforms should be in accordance with the recommendations made with the BSE Minimum Dataset [2]. Within this guideline, diastolic measures have been categorised into: *routine*—measures that are anticipated to be performed in all patients; *supplementary*—additional measures that may be required to confirm LVDF and LVFP; and *non-routine—*measures that are occasionally performed and only in specific scenarios. Practical guidance for how to acquire images and perform the *routine*, *supplementary* and *non-routine* measurements can be found in tables at the end of each section.

### Routine echocardiographic measures of LV diastolic function

### Transmitral E wave

The transmitral Doppler E wave represents early LV filling from rapid relaxation. Although measurement of peak E is not a direct measure of LAP when applying the simplified Bernoulli equation (due to inertial resistance of blood within valve [44, 45]), the peak E velocity is determined by the LA to LV pressure difference and is therefore reflective of LAP. As such, E velocity can be considered alongside other measures for the interpretation of diastolic function and LVFP. In the young healthy heart, rapid relaxation causes low minimum LV pressure that increases the transmitral pressure difference. This creates a suction effect in the setting of normal LAP that enables the majority of ventricular filling to occur in the early diastolic phase and at high velocity, resulting in a high E velocity [46]. With normal aging, slowing of the rate of ventricular relaxation causes the suction effect to become attenuated, in-turn leading to a higher minimal pressure, a reduction in the early diastolic transmitral pressure gradient and a fall in the transmitral E velocity.

#### Transmitral E velocity—impaired diastolic function

Similar to normal aging, impaired LV relaxation results in a slower rate of pressure fall within the LV, an increase in the minimum diastolic pressure and therefore attenuated suction effect. In the setting of normal LAP, this reduces the transmitral pressure difference and results in lower inflow velocities. Peak E velocity therefore falls as LV relaxation becomes impaired (Table 1). As diastolic impairment progresses and LV compliance decreases, LAP becomes elevated causing the E velocity to increase. The relationship between LV diastolic function and E wave velocity is therefore U-shaped, with E velocity falling in the early stages of diastolic impairment before increasing as disease progresses.

**Table 1:** Age/sex specific values for E/A ratio and E velocity from the World Alliance of Societies of Echocardiography

Age and sex-specific cut-off for E/A ratio and E velocity				
Age	18–40 y		41–65 y	> 65 y
Sex	Male	Female	All	All
E/A ratio	< 0.9	< 1.1	< 0.7	< 0.5
E velocity (m/s)	< 0.5	< 0.6	< 0.5	< 0.4

Each value represents the lowest expected ratio/velocity in those with normal diastolic function, any value below that presented is considered abnormal and therefore suggestive of impaired LV diastolic function [46]

#### Limitations of E wave velocity

*Valve disease:* significant MV disease causing elevated LAP will cause the E velocity to increase, altering the E/A ratio irrespective of LV diastolic function [47]. Furthermore, because the peak A wave velocity is flow/load dependent, the E/A ratio may also be affected by moderate or severe aortic regurgitation (AR) that increases LVEDP, decreases the end-diastolic LA-LV pressure difference and therefore decreases A wave velocity [48]. *LV disease:* in patients with coronary artery disease (CAD) or hypertrophic cardiomyopathy (HCM) and normal LVEF (≥ 50%), peak E velocity correlates poorly with LAP [49]. Peak E velocity is heavily influenced by changes in LV volume and LV elastic recoil (and therefore systolic contractility) and should not be utilised as a standalone indicator of LVFP in any scenario. *Flow timing:* although not typically considered for the assessment of diastolic function, it is important when considering timing of flow to bear in mind that flow response to changing pressure is not instantaneous. Transmitral flow velocity increases so long as there is an accelerating force and therefore positive pressure gradient, while a reversal of the pressure gradient acts as a decelerator of flow. The inertial effect of pooled blood in early diastole therefore explains the temporal difference between peak pressure gradient and peak transmitral flow velocity, meaning that the peak pressure gradient and peak transmitral velocity do not coincide to provide exact timings [50].

#### Transmitral E deceleration time

E wave deceleration measures the time between peak early transmitral flow velocity (peak E) and the point when flow ends, or the point at which atrial contraction occurs. Onset of mitral E-deceleration corresponds to LA-LV pressure crossover and reversal of the transmitral pressure gradient which acts as a deceleration force. In patients with a remodelled and stiff ventricle, a large early filling volume, as reflected in a high E velocity, leads to rapid rise in LV pressure and a high deceleration gradient which causes a short E deceleration time. E-deceleration time is therefore a marker of LV diastolic stiffness, although in milder degrees of diastolic impairment E-deceleration time is more influenced by LV relaxation. In young healthy hearts, rapid LV recoil and relaxation result in highly efficient filling and a large volume of blood entering the LV very quickly with consequently rapid equalisation of pressure between the LA and LV [51]. The deceleration of flow is therefore equally rapid with a short time period between peak E velocity and the end of early diastolic flow, reflected as short E wave deceleration. However, as LV relaxation slows with normal aging, the rate and volume of LV filling falls, and there is lengthening of the E wave deceleration time. An extended E deceleration time is therefore expected in the elderly [52].

#### Transmitral E deceleration time—impaired diastolic function

The early stages of impaired diastolic function are characterised by slowing in the rate of LV relaxation with therefore extended E wave deceleration time, as seen with normal aging—typically > 220 ms in those < 50 years and > 280 ms in those > 50 years [53]. As diastolic impairment progresses and LV compliance decreases, LAP increases to maximise LV filling and maintain SV. The early diastolic flow into an incompliant LV causes LV pressure to rise rapidly and LA-LV pressures to equalise quickly with a consequently rapid deceleration of flow. The E wave deceleration time is therefore short (< 150 ms) when diastolic impairment is advanced and LVFP increased. Hence, the relationship between impaired LVDF and the E deceleration is inverse U-shaped (increasing with impaired relaxation before decreasing as LVFP increases), with an inverse relationship between rising LAP and E deceleration time.

In those with confirmed heart disease, decreased LV compliance and restrictive filling are associated with worsening mortality. Given that the E deceleration time relates to LV compliance and therefore filling, there is a demonstrable association with heart failure (HF) symptoms, death and hospitalisation in both those presenting with acute myocardial infarction or HF with reduced ejection fraction (HFrEF). The E deceleration time is therefore of significant prognostic importance in patients with known heart disease [54–56].

#### Limitations of E wave deceleration time

*Normal LVEF:* when LV systolic function is normal, the E deceleration time is not a consistently accurate measure of LVDF [51]. *E/A fusion:* the deceleration time may be unmeasurable when E and A waves are fused due to tachycardia, raised pre-A velocity or first-degree AV block. *MV disease:* the deceleration time is likely determined by the severity of stenosis in patients with mitral stenosis (MS) [47].

### Transmitral A wave velocity

In SR, LV filling concludes with atrial contraction in late diastole, represented as the transmitral A wave on Doppler imaging. A distensible and compliant LV is able to increase end-diastolic volume with only a small increase in pressure. With normal diastolic function, the majority of total LV filling occurs in early diastole with a small contribution of LA contraction to overall filling, usually 10–20%. Therefore, with normal LV compliance, the low pressure difference between the LA and LV in late diastole and the low volume contribution from LA contraction result in a transmitral A wave velocity that is typically lower than the E wave velocity. In young individuals, the A velocity is usually < 50 cm/s [46]*.* However, the age-related decline in LV relaxation reduces early diastolic filling with consequently reduced emptying of the LA [57–59]. Reduced emptying leads to an increase in LA volume (preload) prior to contraction in late diastole resulting in greater LA pump volume and therefore greater A wave velocity, often to around 75 cm/s in the elderly.

#### Transmitral A wave velocity—impaired diastolic function

Similar in physiology and echocardiographic appearance to normal aging, impaired LV relaxation but with normal compliance causes the transmitral A velocity to increase. As diastolic impairment progresses and LV compliance decreases, raised LV end-diastolic pressure increases LA afterload and therefore resistance to LA ejection, leading to a reduction in the transmitral A volume, velocity and duration of flow. Therefore, the relationship between impaired LVDF and A velocity is inverse U-shaped, where A velocity initially increases as relaxation slows before decreasing as LV diastolic pressure increases.

#### Limitations of A wave velocity

*E/A fusion:* may prevent identification of the MV A wave duration and peak velocity. *Aortic regurgitation:* transmitral A velocity is affected by increases in LA afterload. When severe AR significantly increases LVEDP, LA afterload is markedly increased with reduced LA-LV pressure difference and consequently reduced LA pump volume and therefore low A wave velocity and reduced duration [48]. *Pre-A velocity:* the pre-A velocity describes the cross-over point between fused E and A waves. With normal diastolic function and normal resting HR, the pre-A velocity is typically < 20 cm/s. When diastolic function is impaired, the slower rate of relaxation may extend into the later diastolic period (reflected by longer E deceleration time), causing early diastolic flow to continue to the point of atrial contraction and consequently fusing the E and A waves; E/A fusion can also be seen secondary to dyssynchronous relaxation (left bundle branch block (LBBB)), 1st degree AV block or by sinus tachycardia. If atrial contraction occurs at a point when the pre-A velocity exceeds 20 cm/s, the higher starting blood-flow velocity of the associated A wave results in a higher peak A wave velocity that may cause E/A ratio reversal, irrespective of LAP [60]. This discordant finding is common in elderly hypertensive patients with HFpEF where E velocity is increased and exceeds 1 m/s, therefore suggesting increased LAP, while the E/A ratio is reduced, and may be less < 1 due to the pre-A velocity exceeding 20 cm/s. However, the algorithm for the assessment of LVFP remains accurate for the global assessment of LVDF and should be utilised. As with all measures of blood-flow Doppler, signal clarity and consequently measurement accuracy are limited by artefact and the signal-to-noise ratio. Given the relatively low velocity of flow, measurement of the pre-A velocity should be performed once pulsed-wave Doppler (PW) signals have been optimised (minimised wall-filter/low-velocity reject) and transit time artefacts reduced. In cases of eccentric AR where regurgitant flow contaminates the assessment of transmitral forward flow, the pre-A velocity may not be clearly identified and may prevent a measurement from being made.

### E/A ratio

The ratio of transmitral E and A velocities reflects the ratio of early and late LV diastolic filling. Efficient relaxation in young healthy hearts enables the majority of LV filling to occur in early diastole, with relatively low contribution from atrial contraction and at low velocity. The E/A ratio in young healthy individuals is therefore typically > 1. In those who are athletically trained, highly efficient early diastolic relaxation can result in an E/A ratio of up to 2 [61]. Natural aging leads to a slower rate of LV relaxation with a reduction in the early diastolic filling volume and fall in E velocity, normal SV is maintained by an increase in the atrial contraction filling volume [62]. This increase in atrial contraction volume is reflected by an increase in A velocity to exceed E velocity and therefore a shift in the E/A ratio to < 1 [63].

#### E/A ratio—impaired diastolic function

As with normal aging, the early stages of LVDF impairment are characterised by impaired (slowed) relaxation but with normal chamber compliance. As such, E velocity falls and A velocity increases with a reduction in E/A ratio to < 1 (Table 1). As diastolic impairment progresses, LV compliance decreases and both LV diastolic pressure and LVFP increase. The increase in LAP causes E velocity to increase while increased LV end-diastolic pressure increases LA afterload and decreases the end-diastolic LA**-**LV pressure difference, causing A velocity to decrease; E/A ratio therefore returns to > 1 as LVFP increase. As the LV becomes progressively less compliant, filling becomes restrictive with normal or even low filling volumes causing a significant increase in LV diastolic pressure and consequently LAP. Higher LAP leads to increasing E velocity while significantly increased LA afterload results in further reduction of A velocity and subsequently increasing E/A ratio, typically > 2, as restrictive filling develops. Therefore, the relationship between E/A ratio and diastolic function is, like E wave velocity, U-shaped.

#### Limitations of E/A ratio

When the E and A are fused and pre-A velocity exceeds 20 cm/s, the A wave velocity is increased and E/A ratio altered irrespective of LAP. E/A fusion may also prevent identification of the MV A wave duration and peak velocity. *LA afterload:* transmitral A velocity is affected by increases in LA afterload. When severe AR significantly increases LV end-diastolic pressure, LA afterload is markedly increased with consequently reduced LA pump volume and therefore low A wave velocity and reduced duration [64]. *LA function:* when LA pump function is reduced due to cardiomyopathy, CAD, following heart transplantation or stunning following cardioversion to SR from an atrial arrhythmia, the ejected volume is reduced with consequently low A velocity. E/A ratio will therefore be altered by a lower peak A velocity. *Short P-R interval:* the A wave duration may be truncated by a shortened PR interval when LV systolic contraction occurs before atrial contraction has been completed. *Atrial Flutter:* because of the very high atrial rate with consequently low atrial pump volume and velocity, the E/A ratio should not be measured during atrial flutter.

### Early diastolic mitral annular Tissue Doppler Imaging (TDI)—e′

As the fibrous boundary between the LA and LV, motion of the MV annulus throughout the cardiac cycle reflects longitudinal shortening and lengthening of both the LV and LA. In systole, the LV shortens, pulling the MV downwards and towards the LV apex, therefore stretching the LA and increasing its dimensions. Following peak systolic contraction and shortening, the LV relaxes and lengthens in early diastole, pushing the mitral annulus upwards and shortening/compressing the LA. In late diastole, atrial contraction causes further LA shortening, pulling the MV annulus further upwards. The phasic velocity of mitral annular motion therefore reflects myocardial function throughout the cardiac cycle and can be measured by TDI as an indicator of systolic contractility and diastolic relaxation and compliance – S′ reflecting LV systolic shortening, e′ reflecting early diastolic LV relaxation and a′ reflecting atrial contraction in late diastole.

In the healthy heart of the young and middle-aged, MV annular velocities are high during both systolic descent and diastolic ascent [46] reflecting the dynamic function of both the LA and LV. In those who are athletically trained, adaptive cardiac remodelling leads to super-efficient cardiac function and marked predominance of early diastolic filling, typically reflected by systolic and early diastolic MV annular velocities that are supra-normal [61]. With normal LV filling, due to the rapid and efficient relaxation of the healthy heart the onset of early diastolic transmitral blood flow occurs near simultaneously with e′. However, even in the normal heart there are expected regional differences in peak relaxation velocities. Due to differences in the extent of longitudinal motion between septal and lateral walls, septal annular velocities are typically lower in comparison to that of the lateral wall [64]. As the relationship between peak e′ velocity and τ is inverse, where slower relaxation leads to increasing τ and decreasing e′, e′ velocities typically fall with normal aging (Table 2).

**Table 2:** Septal and lateral e′ values suggestive of impaired LV diastolic function

Septal and lateral e′ values suggestive of impaired LV relaxation				
Age	18–40 y		41–65 y	> 65 y
Sex	Male	Female	All	All
Septal e′ (cm/s)	< 7.0	< 8.0	< 5.0	< 4.0
Lateral e′ (cm/s)	< 9.0	< 11.0	< 6.0	< 5.0

Any value below that stated within the table is suggestive of impaired LV relaxation [46]

Similar to the process of aging, impaired LV relaxation and longer τ leads to both a delay in onset of e′ and a fall in peak velocity [65, 66]. When LV relaxation is normal, e′ velocities may be preload dependent (e′ increases as the transmitral gradient increases). When LV relaxation is impaired, the effect of increased LAP on e′ velocity is negligible such that e′ remains low [67, 68]; an e′ below the age-specific cut-off is therefore considered an indicator of impaired LV relaxation. However, due to the wide range of normal e′, with some patients having naturally very high values, a value greater than the age-specific cut-off does not confirm normal LV relaxation.

### E/e′

In normal hearts with normal LVDF and relaxation, e′ velocity is directly related to the transmitral pressure gradient and indexing to E velocity for E/e′ does not correlate with LVFP. When LVFP are normal but the rate of LV relaxation is reduced secondary to either normal aging or impaired LVDF, both E and e′ velocities are reduced. However, because the reduction in both E and e′ is almost proportional, the ratio between them remains relatively unchanged and < 14 in the majority of individuals across all age groups [46]. Progression of impaired diastolic function is characterised by worsening of LV relaxation, with further reduction of e′ velocity, and decreasing LV compliance leading to elevated LVFP and therefore increased E velocity. An increased E/e′ ratio is therefore a marker for increased LVFP and LAP. A value < 8 is specific for normal LVFP and an average of septal and lateral E/e′ > 14 is highly specific for a PCWP of > 15 mmHg and therefore raised LVFP [69, 70]. Where only a single site measure is possible or valid, a lateral ratio > 13 or septal ratio > 15 may be used. Although a progressive age-related decline is observed for of both E and e′ velocity, the proportional decline in e′ is greater than for E with a consequent increase in E/e′ ratio with normal aging, although rarely to a level that suggests elevated LVFP [46]. An E/e′ ratio > 14 is therefore a supportive finding for elevated LVFP for the majority of individuals across all age groups and certainly in those ≤ 40 years.

#### Limitations of E/e′

*Doppler alignment:* Measures of peak e′ velocity may be underestimated when Doppler alignment is not parallel. Although lateral annular velocities are typically higher than septal, parallel Doppler alignment is more easily achieved with the septal annular motion. Doppler alignment with lateral annulus motion should be considered when interpreting annular velocity and diastolic function. *Regional variation and annular tethering:* The correlation between E/e′ and LVFP is not strong in those with LBBB or paced rhythm but is strong when LV systolic function is globally impaired and the LVEF is reduced [71, 72]. As a measure of peak annular velocity, differentiation between active motion and translational motion through tethering with normally functioning myocardium cannot be made by peak velocity alone. Regional differences in myocardial function may therefore complicate the assessment of LV filling and should be considered. The presence of: severe annular calcification; mitral annular repair ring; or MV replacement will also reduce annular velocities and alter the E/e′. The interpretation of septal and lateral e′ is also important during the assessment of pericardial disease with pericardial adhesion often causing reduced lateral wall longitudinal motion and therefore reduced lateral e′ velocity, thus complicating the assessment of intrinsic LV diastolic function [73, 74]. The septal e′ may be reduced post-cardiac surgery, during atrial arrhythmia and in conditions that significantly affect right heart pressure and volume [70, 75, 76]. *LVEF:* the correlation of E/e′ with LVFP is less strong in those with normal LVEF in comparison to those with impaired LV systolic function [69, 77]. *Load dependency:* although less load dependent than E velocity, e′ is not entirely independent of LV loading conditions with septal and lateral annular velocities possibly affected to different degrees by changes in LV preload [78, 79]. e′ may also be increased by alterations in LV preload secondary to severe mitral regurgitation (MR). When increased LV afterload causes a reduction in LV contractility, LV recoil and relaxation are attenuated with an increase in τ and an associated decline in e′ velocity. e′ parameters should therefore be considered alongside the effect of systolic blood pressure (SBP) on LV function. *Age:* e′ reflects LV relaxation and therefore decreases with age; age-specific limits for abnormal velocities should be used to avoid over-classification of the elderly as abnormal. However, although an E/e′ < 14 does not confirm normal LVFP, a ratio > 14 is extremely uncommon in young and middle-aged individuals with normal hearts and is only seen in a very small number of elderly patients with normal LVFP [46].

Although numerous studies have confirmed the utility of E/e′ for the assessment of raised LVFP, it is a single value that should be considered alongside all other parameters of LVDF. It should be viewed in the context of its limitations and should not be considered as a deciding parameter for the assessment of LVDF.

### LA volume

In the normal healthy heart, the LA is a thin-walled chamber that functions under low pressure, usually at a mean of around 8 mmHg. LA dimensions are primarily determined by patient lean body mass and because LA size does not increase due to mere aging alone, LA dilation is therefore considered an abnormal finding across all age groups. A detailed description of LA function can be found in the earlier section of this document.

#### LA volume—impaired diastolic function

The extent to which the LA dilates secondary to impaired LV diastolic function is determined not only by LAP, but also intrinsic LA compliance, general hydration/volume status and compliance of the left atrial appendage to help off-load LAP [80]. Therefore, because the relationship between LA volume and pressure is not linear, a specific LAP does not correlate directly with a specific LA volume. Consequently, the direct correlation between LAP and LA volume is not strong [81, 82]. However, LA maximum and minimum volumes are related to LAP and increase in response to chronic elevation. LA dilation is therefore an expected finding in those with impaired LVDF and chronically raised LVFP.

Importantly, LA compliance is a major determinant of LAP. A stiff and incompliant LA causes increased LAP but limits the degree of LA dilation. For example, patients with dilated cardiomyopathy (DCM) and more compliant LV and LA myocardium are likely to have more severe LA dilation with greater maximum volume than those with restrictive cardiomyopathy (RCM) in whom decreased LA compliance limits the degree of dilation (because a stiff chamber cannot dilate to the same extent as a compliant chamber). However, despite lower LA volume, the stiffer incompliant LA in patients with RCM causes higher LAP in comparison to those with DCM and larger but more compliant LA [83, 84].

#### Limitations of LA volume

*Normal LAP:* There are a number of limitations when interpreting LA size as an indicator of LAP. Although LA dilation is present in the vast majority of patients with elevated LAP, not all patients with large LA have elevated LAP. Other causes of LA dilation should always be excluded, including: atrial arrhythmia, significant MV disease, transplanted hearts, bradycardia, ventricular septal defects, athletic remodelling and high output states [85]. *Normal variation:* LA dimensions do not increase significantly with normal aging. However, the normal range of LA volume is wide across all age groups [46, 86]. In around 90% of non-obese patients with normal healthy hearts, the biplane Simpson’s Method of Disks (MoD) LA volume does not exceed 34 mL/m^2^ when indexed (LAVi) to body surface area (BSA). This value is considered the upper reference interval of normal LA size and therefore the recommended cut-off when assessing LVDF. However, even in entirely normal hearts, LA volume measures > 34 mL/m^2^ in around 10% of patients and > 37 mL/m^2^ in around 5% [85], with some studies reporting LAVi up to 43–45 mL/m^2^ in normal males and females across most age-groups [46]. LA volume alone is therefore not a reliable indicator of LAP. *Obesity:* furthermore, increasing obesity limits the utility of indexing to BSA. Cardiac size is primarily determined by fat-free lean muscle mass—LA dimensions do not increase in response to obesity alone. However, when indexing heart size to patient habitus during echocardiography, BSA is estimated according to simple measures of patient height and weight. The normal age-related decline in adult height has a minimal impact upon BSA. However, increasing weight can significantly increase the BSA, consequently ‘normalising’ chamber volumes when dividing their absolute dimensions by a larger BSA value. The utility of indexed LA measures is therefore reduced in obese patients with decreasing utility as BSA increases. For this reason, and because obesity is common in patients with impaired diastolic function, it has been suggested that a lower LAVi cut-off value may be considered to describe LA dilation in obese patients [66]. However, because the same LA volume will result in progressively lower LAVi as BSA increases, applying a single dichotomous cut-off parameter to this continuous variable for all patients is inherently limited. Interpretation of LA size should therefore be based on a global judgement of cardiac function and alongside the Doppler based parameters of LV filling. *Chronicity:* LA dilation is a chronic process and reverse remodelling is not immediate. LA volume may therefore remain persistently increased despite normalisation of LVFP, for example with the introduction of medical treatment [87]. Similarly, sudden increases in LAP secondary to acute deterioration in LV function (myocardial infarction, myocarditis) may be associated with normal LAVi. However, sudden increases in LAP would likely be reflected clinically and by other parameters of diastolic function.

### TR velocity

The continuity between the LA, pulmonary veins and the pulmonary capillary system causes elevation of LAP to be transmitted through the pulmonary vasculature, leading to elevated pulmonary pressures. Therefore, in the absence of other causes of PH, the velocity of tricuspid regurgitation (TR) can be considered indicative of systolic pulmonary artery pressure (SPAP) and therefore incorporated into the assessment of LVDF and estimation of LVFP.

#### Limitations of TR velocity

*Doppler:* like all Doppler measures of blood flow, estimation of TR velocity is subject to error associated with the angle of insonation and alignment between the transmitted sound and the blood flow. Although perfect parallel alignment is not achieved in most cases, the degree of velocity underestimation increases as the angle of misalignment increases. Crucially, some degree of TR is required in order to measure the peak velocity. However, even when present, it is very often of insufficient volume to provide a complete Doppler waveform and therefore of measurable peak velocity [46]. *Other causes:* as any cause of PH will cause TR velocity to increase, the presence of undiagnosed pulmonary disease may lead to a TR velocity that exceeds 2.8 m/s [88]. *Very severe/torrential TR:* when TR becomes very severe or massive/torrential, the large volume of blood entering the RA causes RAP to rise rapidly and become markedly elevated, leading to rapid equalisation of RA and RV pressure. Although systolic pulmonary pressures may be elevated, the very high RAP results in low pressure difference between the RV and RA and therefore low TR velocity with consequently underestimated SPAP. In this scenario, the waveform of the TR continuous wave (CW) Doppler can help identify when the severity of regurgitation is such that the peak velocity is underestimated. The CW Doppler waveform of non-torrential TR is typically parabola in shape due to the pressure difference between the RV an RA being sustained throughout systole, and therefore sustaining the velocity of regurgitant flow. However, torrential TR causes RAP to rise to very high levels and rapidly, equalising with RV pressure and leading to a rapid cessation of TR. This rapid equalisation of pressures with no sustained pressure difference throughout systole results in a triangular CW Doppler waveform and subsequently underestimated peak TR velocity. Therefore, a velocity of ≤ 2.8 m/s does not confirm normal SPAP when TR is massive/torrential. *Underestimation of RAP:* as the TR velocity merely reflects the pressure difference between the RV and RA, any increase in RAP will reduce the velocity of TR. As such, a TR velocity below the threshold of 2.8 m/s may be seen in the setting of elevated SPAP when RAP is also raised.

### LA function—strain analysis

Although atrial function can be assessed by measuring LA volume at specific points of the cardiac cycle, this is a time-consuming process and is limited by low temporal resolution (low frame-rates) at higher heart rates. Strain analysis is considerably quicker and is largely automated, and therefore lends itself more readily for use in clinical medicine. The analysis of LA strain (LAs) provides parameters for all phases of LA function, the reservoir phase (LARs) and pump phase (LAPs) being important for the assessment of LVDF.

**Fig. 10:**
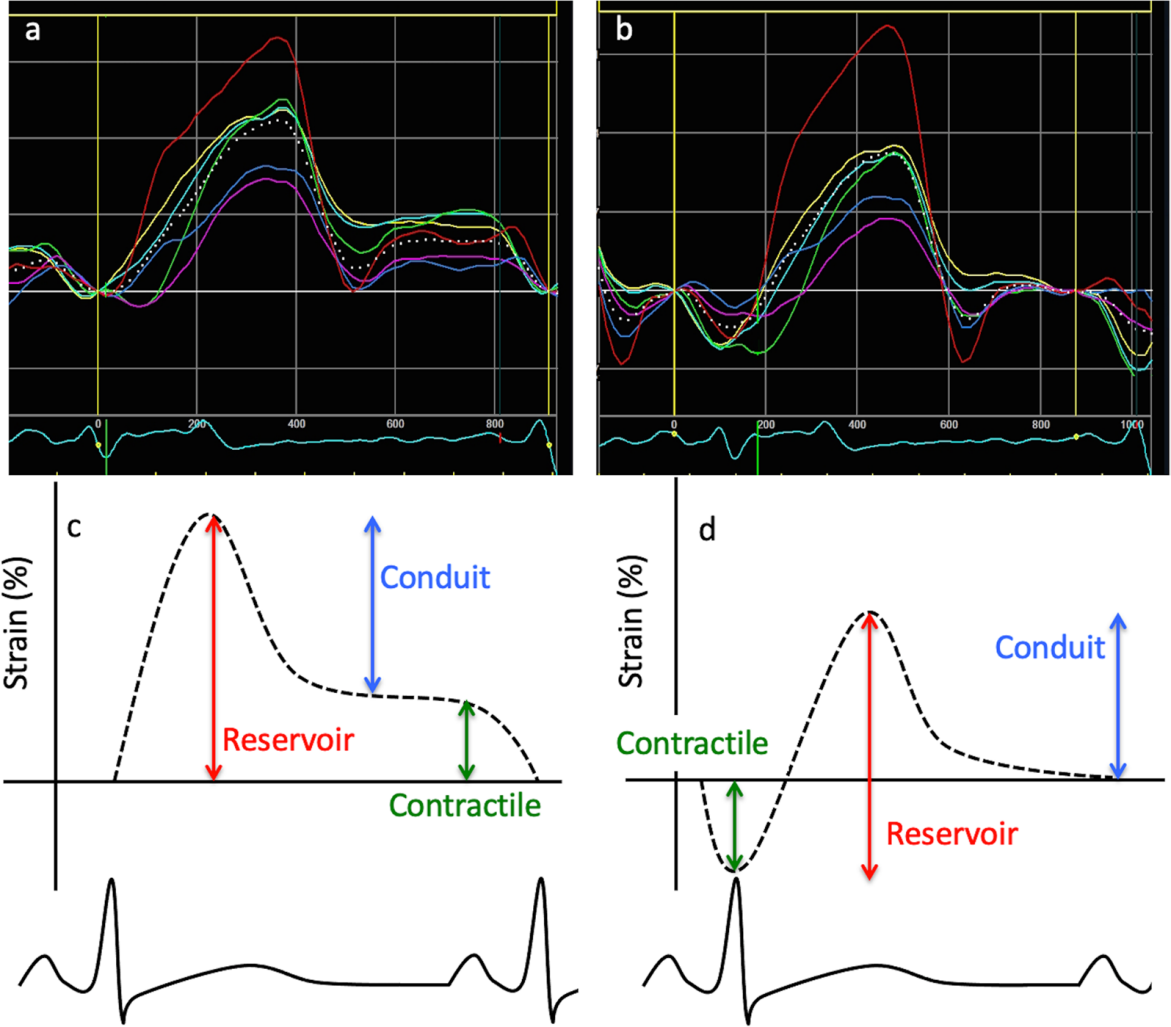
Atrial strain, with examples of different zero-reference points. Figure (**a**) displays a typical LA strain waveform while (**b**) demonstrates measured atrial strain parameters. On the lower left (**c**), the onset of the QRS has been chosen as the zero-reference point. With this method, the atrium is at the beginning of the reservoir phase and initially expands, resulting in a *positive* deflection (**c**). On the right, the p-wave is used as the zero-reference, and consequently the first deflection is *negative* as the atrium enters the pump phase (**d**). Red vertical arrow demonstrates peak reservoir strain, blue vertical arrow the passive contraction strain (conduit phase), and green vertical arrow active pump (contractile) strain (**c** and **d**). The choice of zero-reference point will systematically alter the values obtained, with the QRS-onset methodology (**c**) leading to systematically larger strain values than the p-wave methodology

Two methodologies for the assessment of atrial strain have been established yet differ according to the zero-reference point (i.e., the defined starting point of analysis; Fig. 10). The convention for measuring LV strain is that relative fibre *shortening* is associated with compression of the myocardium and therefore a ‘negative’ strain value, and relative fibre *lengthening* with a ‘positive’ value. Although this remains the principle for assessment of LAs, the point in the cardiac cycle at which the measurement starts will determine the calculated strain value.

When the peak of the R-wave (i.e., onset of ventricular systole) is used as the starting point for LAs analysis (R–R gating), the zero-reference point coincides with the onset of the ‘reservoir’ phase. In this setting, because analysis starts at the point when LA dimensions are at their smallest, the first observed change in LA myocardial length occurs as the LA increases from its minimum to its maximum size; the associated fibre elongation therefore results in a strain value that is *positive* in reference to the pre-lengthening baseline. Alternatively, if the onset of the P-wave is chosen as the zero-reference point (P–P gating), analysis will begin immediately prior to the onset of the pump phase and LA shortening, in which case the initial strain deflection will be *negative* as the atrial myocardium contracts and shortens. Therefore, because strain assessment describes the *proportional change* in myocardial length, the differences in zero-reference point becomes important. For example: if strain analysis starts with a pre-contractile atrial fibre length of 4 cm, and the contracted length shortens to 2 cm, this reflects a strain of -50%. However, if analysis of the same segment of atrial tissue starts at a contracted length of 2 cm, which then relaxes to a length of 4 cm, strain is calculated at + 100%. Although significantly different values, both describe the same absolute change. For this reason, the choice of zero reference point systematically alters the strain values obtained, with the QRS-gating providing systematically larger values for atrial strain than the p-wave gating. However, the proportional values of reservoir vs. pump strain will remain the same irrespective of the method chosen.

In the majority of published data, the QRS is defined as the zero-reference point and is therefore the preferred method of the BSE [89]. According to vendor, strain analysis may be performed in a single A4C view or a global average by biplane from both the A4 and A2C views optimised for maximal LA dimensions [2]. As with estimates of LA maximum volume, A2C and A4C views optimised for the LV do not provide the greatest LA volume and therefore overestimate LAs values [90]. LAs values do not differ between men and women and normal reference intervals have been described by both meta-analysis and study of normal healthy hearts [91] (Table 3).

**Table 3:** Normal reference intervals for LA reservoir and pump strain. Adapted from Singh et al. [91]

Lower and upper limits of normal left atrial strain parameters						
Age	Male 18–40 y	Male 41–65 y	Male > 65 y	Female 18–40 y	Female 41–65 y	Female > 65 y
LA reservoir strain (%)	25–63	23–61	24–57	29–62	22–56	21–56
LA pump strain (%)	2–23	5–28	9–32	2–21	6–28	7–30

*LA reservoir strain—*LA dimensions increase from a post contraction minimum at end-diastole to a maximum at end-systole. The positive reservoir strain therefore reflects total LA lengthening.

*LA pump strain—*LA dimensions decrease as the LV lengthens in early diastole with further reduction in LA dimensions following atrial contraction. Pump strain values are therefore negative.

#### LA strain in the assessment of LV diastolic function

It is well recognised that dilated atria are associated with a poorer prognosis across a range of cardiovascular disease states [92]. However, increased LA volume does not confirm increased LA pressure, nor does it identify LA function. For instance, LA dilation is known to occur secondary to certain normal physiological conditions, including athletic conditioning and prolonged bradycardic states. In both scenarios, LA pressure and function remain normal, suggesting that function rather than volume may be more relevant when investigating diseases that affect LVDF [93].

The clear and evidenced disturbances of LA function in a variety of cardiac diseases [94–100] reflects the sensitivity and utility of LAs for the assessment of impaired LV diastolic function. The measurement of LAVi has long been considered crucial for the assessment of LVDF on the premise that chronically increased LAP leads to LA dilatation [85]. While this is often the case in the long-term, overlap with normal physiological variation and the low sensitivity of LAVi for the early detection of increased LAP limit the accuracy of LAVi as a reliable and sensitive marker for abnormal increases in LAP. However, because the LA is exposed to LV pressures throughout the diastolic period and because LA function and emptying is influenced by LA afterload and properties of LV systolic shortening and diastolic lengthening, parameters of LAs can be considered indicative of LV diastolic relaxation and filling pressures and are therefore a useful parameter in the assessment of LV diastolic function.

When LV relaxation is impaired but with normal LVFP and normal LAP, reduced lengthening of the LV in early diastole causes a reduction in LA shortening and consequently reduced LAs during this period (conduit phase), while the shift to proportionally greater LV filling from LA contraction causes an increase in LAPs. However, LAPs is also affected by properties of intrinsic LA contractile function and LA afterload at end-diastole (LVEDP), therefore directly correlating with MV A and a′ velocity. Given the influence by LVEDP, LAPs is also related to A-wave transit time and inversely related to Ar-A duration (see later sections) [85].

Although studies have demonstrated an inverse relationship between LARs and LAPs for both PCWP and LVEDP irrespective of LVEF, the correlation is not strong enough to justify LAs as a standalone measure of LVFP and therefore diastolic function. However, the correlation is stronger than that of LAVi and may be help differentiate severity. When individuals with impaired diastolic function and similar LV size, mass, ejection fraction and, most importantly, left atrial volume were compared, LAs parameters were found to be significantly lower in those with symptoms of HFpEF, thus reinforcing the importance of LA function over size alone [101]. Furthermore, and suggesting potential prognostic importance of LA function, when comparing patients with HFrEF and HFpEF, although those with reduced LVEF tend to have larger LAVi, those with preserved LVEF have greater LA stiffness with consequently lower LAs values and more AF [83, 84].

**Fig. 11:**
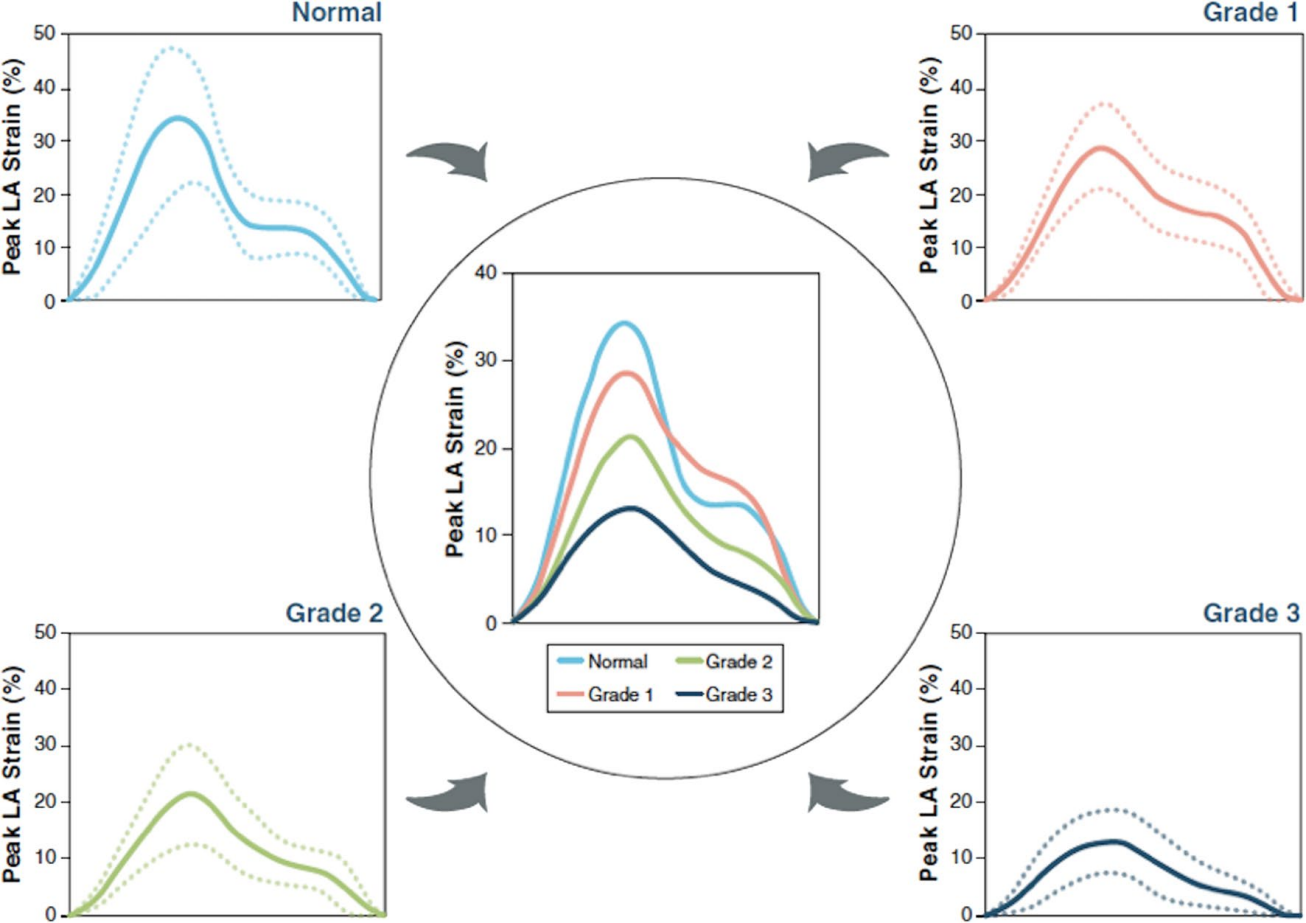
Step-wise reduction in LA strain parameters as LV diastolic function worsens and LVFP increase. From Singh et al. [102]

A step-wise reduction in LARs is observed as LVDF worsens, with LARs found to be the only parameter of LV filling that consistently deteriorated as LV diastolic impairment worsened, thus distinguishing all grades of diastolic function [102] (Fig. 11).

In contrast, conventional measures of LVDF, LAVi and E/e′, increase and therefore differentiate normality from impaired diastolic function with raised LVFP, before plateauing with no identifiable differentiation between higher grades of diastolic impairment (raised LVFP versus restrictive filling). Consistent with a continuous deterioration in LA function as diastolic impairment progresses, the prevalence of abnormal LARs appears to be significantly linked with the severity of impaired diastolic function, with a reported increase from 62.9% in those with normal LVFP, to 88.6% in those with raised LVFP and 95.7% in those with restrictive filling. A step-wise decline in LARs is also observed as LVDF worsens, from 22.2% (± 6.6%) in those with normal LVFP, 16.6% (± 7.4%) in those with raised LVFP and 11.1% (± 5.4%) in those with restrictive filling [84].

Despite this continuous stepwise reduction in mean LARs across all grades of diastolic impairment, there exists an overlap in strain values between the normal cohort and those with impaired relaxation but normal LVFP, such that LARs may be insensitive to accurately identify the early stages of LV diastolic impairment [102]. However, the significant differences in LARs between those with normal filling and those with raised LVFP identifies LAs as a useful adjunct to the assessment of LVDF. In those with confirmed cardiac disease, PCWP > 12 mmHg was identified by LARs < 18% and absolute LAPs < 8%, while < 16% and < 6% identified PCWP > 15 mmHg [103, 104]. Importantly, in those with normal LV systolic function and absolute GLS 18%, high-normal LA pump strain (> 14%) identified normal LVFP with 92% accuracy [103]. In the same study, the authors reported 83% accuracy of the 2016 ASE algorithm for differentiating normal from raised LVFP, this level of accuracy remained the same when LAs was incorporated into the assessment. However, because a parameter was missing in 10% of patients, most commonly TR, feasibility of the 2016 algorithm improved from 90 to 99% by adding LAs [103]. On this basis, LARs and LAPs can be considered during the assessment of LVDF with a cut-off of < 18% and < 8% suggesting increased LVFP [104].

#### Reproducibility of LAs: inter/intra-observer and vendor variability

Reproducibility of LA strain measures was found to be high during inter-observer variability analysis with inter-class correlation coefficient (ICC) for LARs and LAPs of 0.89 and 0.82 respectively. Intra-observer ICC for LARs and LAPs was also found to be excellent at 0.93 and 0.92 respectively [103].

#### Limitations of LA strain

*Image axis:* off-axis imaging leading to foreshortening of the LA (when apical views are optimised for the LV) overestimates LAs values. *Feasibility:* measurement of LAs is considered feasible in 95% of patients but training and validation may be required for those unfamiliar with LAs measurements. *Normal systolic function:* the correlation between LAs and LVFP is weaker in those with LVEF ≥ 50% in comparison to those with LVEF < 50%. *Atrial arrhythmia:* in patients with AF, LARs is routinely low and typically < 20% [103]. *Vendor variability:* when comparing measures performed on different analysis platforms, there is overall minor variation in the strength of the correlation between measures of LAs and invasively measured LAP across multiple vendors. However, the referenced cut-offs within this guideline have been derived from aggregated data collected by multiple vendors and can therefore be applied irrespective of the ultrasound system manufacturer [103]. To minimise the risk of vendor variation influencing LAs measures and therefore the diagnosis of LV diastolic function, it is recommended, where possible, that surveillance/repeat echocardiography is performed using the same vendor analysis platform as previous studies. Where this is unfeasible, potential vendor differences should be considered within the global interpretation of LV diastolic parameters (Table 4).

**Table 4:** Table of the routine measures of LV diastolic function

Routine measures of LV diastolic function	
*Transmitral Doppler signals*	
In the apical 4-chamber view, place the PW Doppler sample volume (1–3 mm) at the level of the mitral leaflets. Colour flow Doppler must be used to align the sample with the centre of transmitral flow. This is especially important in the setting of LV dilation when transmitral inflow may be directed postero-laterally due to tethering of the mitral leaflets. Spectral gain, wall filters (100–200 MHz), baseline/scale and signal reject should be optimised to ensure a clear signal that identifies the onset and cession of transmitral flow	
*Peak E and A velocity*	
*E wave velocity and deceleration time* *E wave velocity:* peak modal velocity at the leading edge of the transmitral flow in early diastole (following the T-wave). *E deceleration time:* Time from the peak E-wave velocity to the point at which the E wave signal ends, measured along the deceleration slope of E wave signal—either when LA-LV pressures equalise and flow ends at the zero-velocity baseline or at atrial contraction and the onset of the A wave. When the E deceleration slope is bi-modal, the second and typically longer deceleration slope should be measured. For AF or variable R-R, ensure the two preceding R-R intervals are similar and that the heart rate < 100 bpm *A wave*—peak modal velocity at the leading edge of the transmitral flow in late diastole (after the P-wave). Should not be measured in the setting of atrial arrhythmia *E/A ratio—*peak of the modal E velocity is divided by the peak of the modal A velocity	
*Pre-A velocity*—is only relevant and should therefore only be considered when the degree of E and A wave fusion causes the pre-A velocity to exceed 20 cm/s. Spectral Doppler gain and reject should be optimised to reduce transit time artefact. The velocity is measured at the point where the E and A waves meet.	
*Mitral annular Tissue Doppler Imaging*	
The apical 4-chamber axis should be optimised for the annular region being sampled, ensuring the apically directed movement of the annulus is parallel to the cursor—this may require image adjustment when moving from the septal to the lateral wall. Place the PW tissue Doppler sample volume (5–10 mm) at or within 1 cm of the insertion of the mitral valve leaflets. Both the septal and lateral walls should be sampled and averaged where possible	
*e′*—peak modal velocity at the leading edge of the spectral waveform in early diastole (after the T-wave). Gain and reject settings should be optimised to display high amplitude annular velocities with clearly defined modal waveform. Measurements should be averaged over 3 cardiac cycles, at end expiration	
*Average E/e′*—MV E velocity divided by the average of septal and lateral wall e′ measurements. When only the septum or lateral wall are hypokinetic with no further regional wall motion abnormalities present, annular velocities at the base of the impaired wall are unlikely to reflect global myocardial function and should be avoided. Both measures should be made at end expiration and during similar R-R interval to ensure accurate comparison of values	
*LAVi*	
LA volume should be measured at left ventricular end systole (largest LA size at the frame just before mitral valve opening) using the Simpson’s biplane MoD and then indexed to BSA. Since apical views that are optimised for the LV will foreshorten the LA, dedicated apical 4-chamber and 2-chamber images should be acquired to maximise LA dimensions. The difference between the length of the 4-chamber (A4C) and 2-chamber (A2C) views should not exceed 5 mm [105]. Although simultaneous biplane acquisition utilising 3D imaging may improve measurement accuracy, the A4C and A2C views are not simply orthogonal imaging planes and each view must be optimised to maximise LA dimensions. Compression of the LA from the aorta may invalidate the measurement. Do not include the mitral valve tenting area below the annulus, appendage or pulmonary veins in the trace. The Area-Length method produces values that are 10–15% higher than the Simpson’s method [106]. The BSE therefore recommend the Simpson’s method of LA volume estimation [80]	
*TR velocity*	
Place the CW Doppler sample through the TR flow, using colour flow Doppler to guide. Ideally, the CW cursor should be placed through the TR flow convergence zone (PISA) and vena contracta to achieve maximum TR velocity. Obtain the peak velocity from either the A4C, parasternal short-axis or RV inflow view. Optimise the gain and reject settings to obtain a complete envelope with elimination of transit-time artefact. If in AF, averaging over five to ten consecutive beats can be performed. However, where possible, a single measure can be made if specific criteria are met—when the preceding and pre-preceding RR intervals are within 60 ms of each other and both exceed 500 ms, measures of a single beat are similar to those averaged over 15 cycles of varying durations [107]. Although there is little data available for the assessment of TR velocity in AF, these findings suggest that selection of beats with similar RR intervals is more important for reproducibility than the total number of measures made and should be considered when estimating SPAP	
*LA strain (reservoir and pump)*	
The method for measuring left atrial strain varies according to vendor and is semi-automated on most platforms. When acquiring images for LA strain analysis, dedicated atrial windows should be acquired to maximum LA volume. LA strain analysis performed in views optimised for the LV and therefore foreshortening the LA leads to overestimation of LA strain values. Zooming on the LA is likely to improve image accuracy. Although both monoplane and biplane LA strain analysis methods are available, there is no significant difference in calculated values between the two methods. Red trace—reservoir phase, blue trace—conduit phase, green trace—pump phase	

### Supplementary parameters of LV diastolic function

### Transmitral A wave duration (for Ar–A duration)

The transmitral A wave and pulmonary venous a-wave (PVa) flow are caused by atrial contraction and therefore occur simultaneously at end-diastole. When the LV is compliant, EDP is low and offers low resistance to atrial contraction, resulting in greater volume of flow moving forward into the LV than that of flow ejected back into the PV’s. However, despite the differences in volume and velocity, there is no difference in duration between MV A and PVa flow when diastolic function is normal [108].

#### Transmitral A wave and duration—impaired diastolic function

Similar in physiology and echocardiographic appearance to normal aging, impaired LV relaxation but with normal compliance causes the transmitral A velocity to increase but with no reduction in the duration of flow. As diastolic impairment progresses and LV compliance decreases, elevated LV EDP increases LA afterload and therefore resistance to LA ejection, leading to a reduction in the transmitral A volume, velocity and duration of flow.

#### Limitations of A wave velocity and duration

*Doppler optimisation:* measurement of the MV A wave duration may be prevented if the start and end of flow cannot be identified due to poorly optimised spectral Doppler signals. *Aortic regurgitation:* transmitral A duration is affected by increases in LA afterload. When severe AR significantly increases LVEDP, LA afterload is markedly increased with consequently reduced LA pump volume and therefore low A wave velocity and reduced duration [48]. *Short P-R interval:* the A wave duration may be truncated by a shortened PR interval when LV systolic contraction occurs before atrial contraction has been completed. *Doppler alignment:* sampling of PV flow becomes increasingly difficult as LA volume/dimensions increase, a common finding in those with impaired diastolic function or atrial fibrillation.

### Pulmonary vein flow

**Fig. 12:**
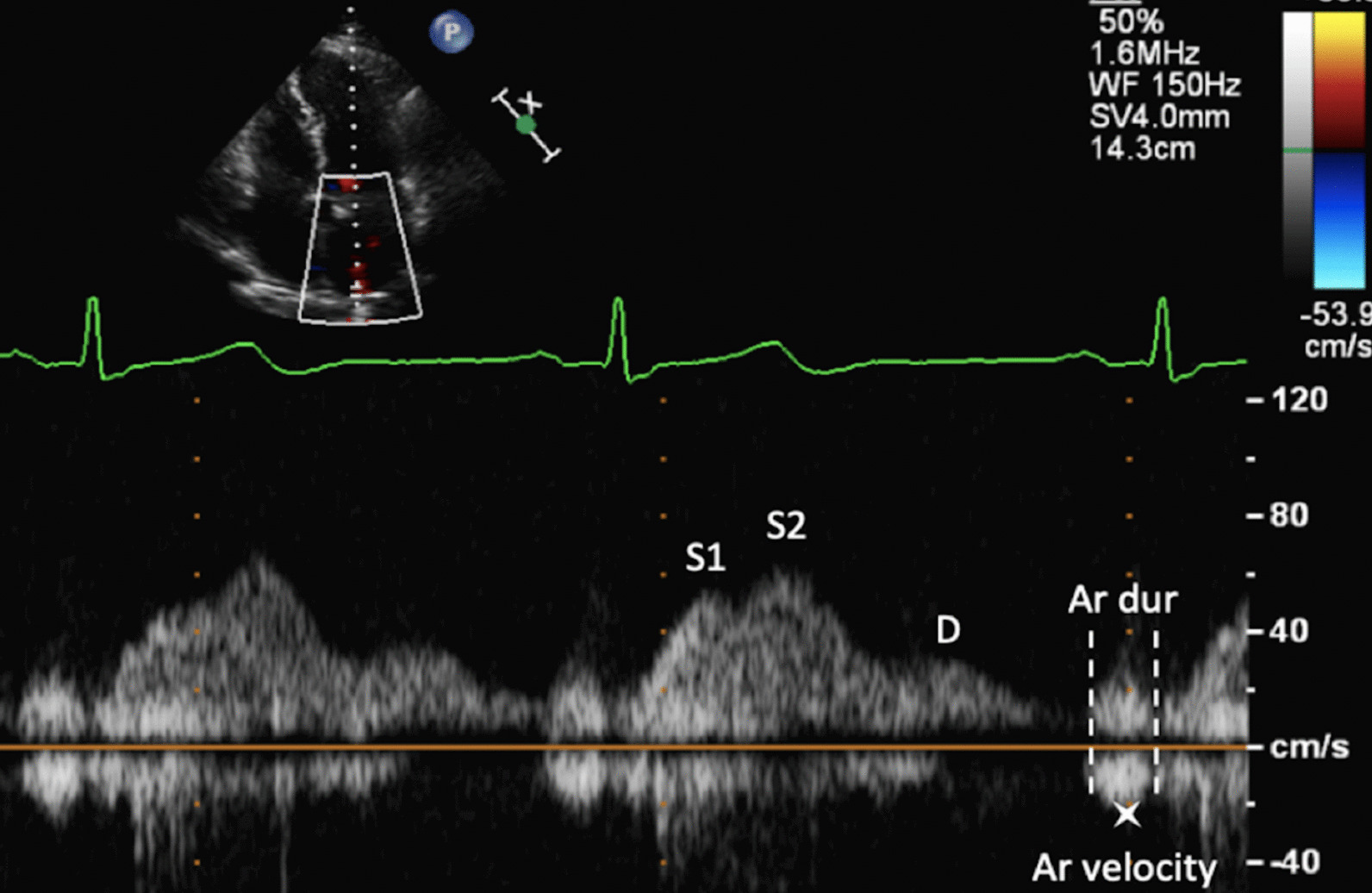
PV Doppler: S^1^ and S^2^ waves occur in systole, contributed to by elastic recoil of the LA, LV systolic shortening and RV SV/SPAP propagating through the lungs. The D waves occurs with LV relaxation in early diastole while the Ar wave occurs in late diastole following atrial contraction

Phasic flow through the PV’s in governed by LV relaxation, compliance of both the LV and LA and by LA function. As such, PV flow profiles provide insight into LV filling and diastolic function. In SR, PV flow occurs over three phases (Fig. 12). Although the deceleration times for the PV S and PV D waves are not supplementary parameters within the diastolic algorithms (they are considered non-routine measures), they are described below within the broader description of pulmonary vein flow.

#### PV S wave velocity and deceleration

The PV S wave may have two identifiable peaks that correspond to the two phases of flow during this period: S^1^ and S^2^. During LV systole and immediately after contraction, elastic recoil of the LA causes dimensions to increase and pressure to fall, drawing a small volume of blood from the PV’s into the LA by suction effect—identified as the S^1^ wave. During mid and late systole, ongoing shortening of the LV and descent of the mitral annulus towards the apex further increases LA dimensions, while RV SV and systolic pressure are propagated through the lungs. The combination of these processes drives a second S wave—identified as S^2^. Overall, because the volume of PV flow generated by LA recoil is minimal, S^2^ is the predominant contribution to the peak S wave velocity (Fig. 12). Consequently, the PV S wave velocity is not only influenced by LV diastolic properties and LAP, but also by LA contraction (that determines the magnitude of elastic recoil), LA relaxation and compliance, LV compliance as well as LV and RV contractility.

#### PV D wave velocity and deceleration

Rapid early diastolic relaxation of the LV causes pressure to fall below LAP, creating a suction-effect that opens the MV and draws blood into the LV from the LA. As the LA empties, the fall in LAP causes blood to flow from the PV’s through the LA and into LV—identified as the PV D wave (Fig. 12). The reduction in LA dimensions during this period is secondary to relaxation and lengthening of the LV, pushing the MV annulus upwards and towards the roof of the LA, with no real mechanical contribution by the LA. After peaking in early diastole, PV D flow gradually decelerates until the end of early diastolic filling, corresponding to the deceleration of transmitral flow. As PV D velocity and deceleration reflect transmitral forward flow, it is therefore altered by variations in LV relaxation rate and LAP.

#### PV Ar velocity and duration

Following the early LV filling phase, atrial contraction at end-diastole contributes to the remainder of total LV filling. However, while normal LV compliance and normal/low EDP allows for most of the blood ejected by the LA to enter the LV, a small volume of blood is ejected back into the PV’s (Fig. 12). The velocity and duration of retrograde flow into the PV’s is therefore determined by a number of factors, including: atrial preload, afterload and intrinsic LA pump function. When LVDF is normal, the duration of retrograde flow into the PV’s is equal or very similar to that of transmitral flow into the LV.

#### PV flow—impaired diastolic function

#### PVS/PVD ratio and systolic fraction

When diastolic function is normal, PV S velocity is typically equal to or higher than PV D velocity and the ratio of S/D is therefore typically > 1; in athletes or the young and fit, highly efficient and rapid relaxation and predominance of early filling often causes the PV D velocity to exceed the PV S velocity and a ratio of < 1. Reduced LA compliance and pump function and increased LAP secondary to impaired diastolic function may cause the PV S^1^ velocity to fall with a consequent reduction in the combined PV S velocity [109], thus reducing the PV S/D ratio to < 1. The PV systolic fraction describes the ratio of PV S and D flow and can be calculated by PVS_VTI_/(PVS_VTI_ + PVD_VTI_). A systolic fraction of < 40% in those with reduced LVEF provides high specificity for raised LAP [110, 111].

#### Deceleration of PVS slope

**Fig. 13:**
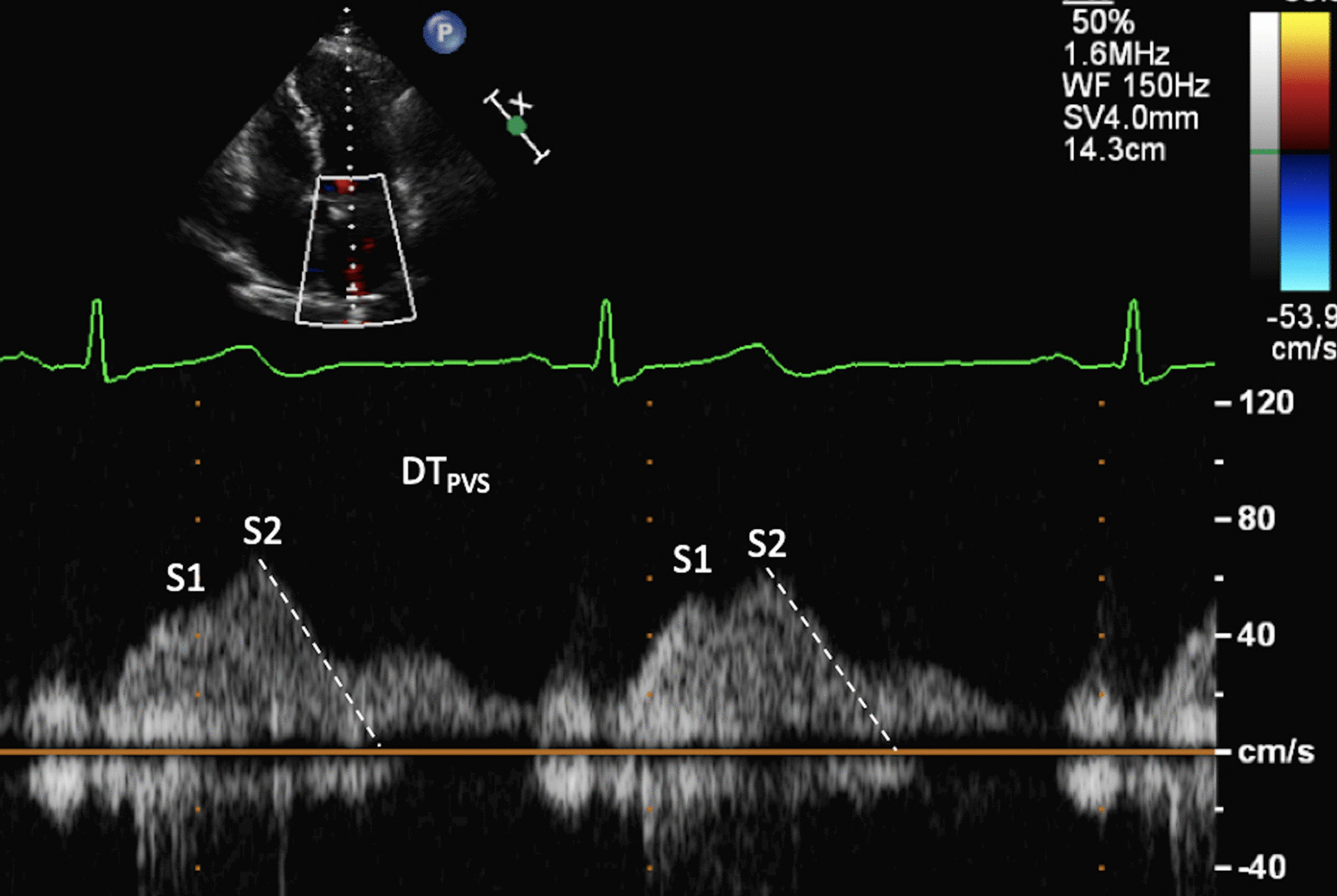
Deceleration slope of the PVS wave—DT_PVS_

The deceleration slope of the PV S wave can be measured as an indicator of LA compliance and therefore LAP (Fig. 13). When the LA is compliant, chamber distensibility results in a gradual increase in pressure and therefore gradual deceleration of the PV S wave. Increased LAP decreases LA compliance and causes pressure to rise more rapidly with filling from the PV’s, leading to rapid equalisation of pressure between the PV’s and LA and therefore rapid deceleration of PV S flow [112]. Although no validated diagnostic parameters are available in order for this measure to be considered routinely, sudden or interval changes in PV S deceleration may provide insight into alterations in LAP [113, 114].

#### PVD deceleration slope

**Fig. 14:**
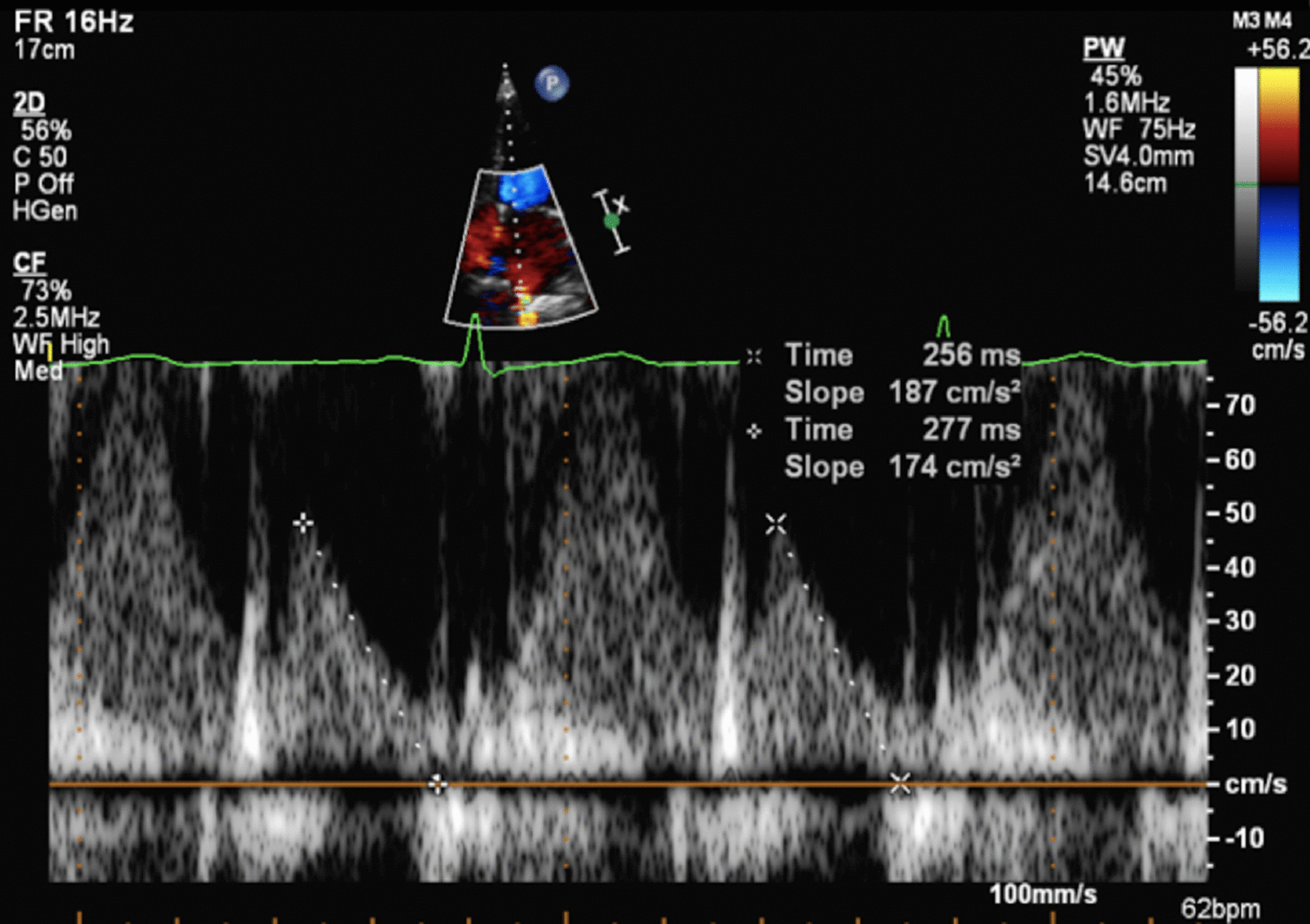
Deceleration slope of the PVD wave—DT_PVD_

Early diastolic LV filling (MV E wave) and PV D flow occur simultaneously. Because LA function provides no real contribution to PV flow during this phase, both MV E and PV D flow profiles are influenced primarily by LV relaxation, while PV D flow is additionally influenced by LA compliance and LAP rise. The peak velocity of PV D flow increases as LAP increases while the deceleration slope of the PV D wave (DT_PVD_) reflects the rate of pressure equalisation between LA and LV (Fig. 14) and, therefore, the rate of LAP increase. When impaired diastolic function is confirmed, shorter DT_PVD_ is suggestive of elevated LAP while longer deceleration time is suggestive of normal/low LAP [109]. A DT_PVD_ of < 175 ms was found to have 100% sensitivity and 94% specificity for identifying LAP ≥ 17 mmHg, while a deceleration time of > 275 ms had sensitivity of 88% and specificity of 95% for LAP of ≤ 6 mmHg in patients in SR with normal LVEF and undergoing cardiac surgery (coronary artery bypass grafting and/or aortic valve replacement) [109]. As no validated diagnostic parameters are available for this measure across large patient groups, it is not recommended in routine practice. However, sudden or interval changes in PV D deceleration may provide insight into alterations in LAP.

#### PV Ar velocity and duration 

Increased LV diastolic pressure secondary to decreased LV compliance increases LA afterload and resistance to LA ejection. Consequently, the volume, velocity and duration of transmitral forward flow from atrial contraction (MV A) decreases when LV EDP is increased. Simultaneously, because resistance to LA ejection is lower in the PV’s than in the LV, a greater volume of blood is ejected back into the PV’s and over a longer duration [69, 115, 116]. The PV Ar velocity and the difference in duration between MV A and PV Ar (Ar-A duration) can therefore be considered as indicators of raised LV diastolic pressures, with an Ar-A duration of > 30 ms or a PV Ar velocity > 35 cm/s suggesting increased LAP [82, 117, 118]. As the only echocardiographic measure of LV pressure at end-diastole, this parameter may help differentiate patients with impaired relaxation but normal LVFP from those with raised EDP only, the first haemodynamic indicator of impaired diastolic function.

#### Limitations of PV Doppler

*Doppler:* measurement of PV flow by PW Doppler may be unattainable due to poor signal quality and in some cases may not be detected at all. Even when attainable, spectral Doppler waveforms of PV flow velocity and deceleration times may be inaccurate when the direction of sound and blood flow are not parallel. However, sampling of the right upper or right lower PV (RU/LPV) in the A4C view usually provides near-parallel alignment with flow. Although measurement of PV S and PV D velocity is possible with good spectral Doppler signals, the PV Ar signal is of lower velocity and short in duration; measurement of PV Ar is therefore subject to greater measurement error, limiting the accuracy of Ar-A duration estimates. The very short time-interval being measured may also be affected by the non-simultaneous method of measuring MV A and PV Ar signals. *MV disease:* the deceleration of PV S wave may not be specific for LV diastolic function. When significant MV disease causes elevated LAP with consequently reduced LA compliance, the PV S deceleration time may be reduced irrespective of LV diastolic properties; even non-severe jets of mitral regurgitation directed into the RUPV can reduce the PV S velocity and consequently reduce the PVS/PVD ratio, irrespective of LVDF and LAP [47]. Additionally, because the PV S velocity is also influenced by the degree of LV longitudinal shortening and RV SV (thereby RV systolic function), the PV S/D ratio may not be consistently < 1 when RV function remains normal despite impaired diastolic function and raised LAP. *LVEF:* the correlation between S/D ratio and LAP is best in those with reduced LVEF [110, 119]. Although these limitations prevent the PV S/D ratio from being routinely considered for the diagnosis of LV diastolic function, in patients with confirmed impaired diastolic function and raised LVFP (irrespective of LVEF), mortality is markedly increased in those where the PV S/D ratio is < 1 in comparison to those where the ratio is > 1, suggesting a possible prognosticating role in those where impaired diastolic function is confirmed [120, 121]. *HR:* HR should be considered when interpreting PV flow for the assessment of LVDF. At lower HR, the low PV flow-rate towards the end of the PV D phase creates little resistance to retrograde flow following atrial contraction and flow reversal is seen during the PV Ar period. However, at higher HR, the shorter diastolic period results in atrial contraction occurring earlier in diastole when PV D forward flow-rates are higher and offer greater resistance to retrograde ejection. Therefore, despite increased LAP, PV Ar waves may not be present during periods of tachycardia [122]—if present, velocity and duration are likely to be affected. The assessment of Ar-A duration is therefore less valid in sinus tachycardia and long PR intervals with E and A fusion and cannot be measured in AF or atrial flutter. Accuracy of the systolic fraction is reduced when LVEF is normal or in the presence of AF, MV disease or HCM. *Healthy young:* in healthy young individuals and athletes, enhanced LV relaxation causes a suction effect that increases the PV D velocity and may lead to S/D ratio reversal. Although this PV pattern may also be accompanied by high E/A ratio and possibly larger LA size in athletes (potentially giving the impression of impaired diastolic function), this is easily recognised as a normal finding by analysis of the e′ velocity, E/e′, TR velocity and LA function (see later section).

### L-wave

Although LA and LV pressures are near equal during diastasis, continued PV flow during this period causes a continued rise in LAP. In normal healthy hearts, this momentum of pulmonary vein flow entering the LA and causing LAP to rise results in very low velocity transmitral forward flow, usually measuring < 20 cm/s [39].

#### L-wave—impaired diastolic function

Continued PV flow into a dilated and incompliant LA may cause consistent elevation of LAP following early LV filling, leading to transmitral flow during diastasis with a velocity that reflects the degree of LAP elevation [22]. Although some degree of transmitral flow may be seen in normal circumstances, high velocity flow (> 20 cm/s) during diastasis reflects impaired relaxation and elevated LAP [23].

#### Limitations of L-wave

The limitations for measuring and interpreting both the L-wave and pre-A velocity are similar given that both describe the point in mid-diastole between early and late LV filling. *E/A fusion:* whereas the pre-A measure describes the velocity of transmitral blood-flow at the cross-over point between E and A waves, the L-wave velocity is a measure of transmitral flow occurring between two identifiably separate E and A waveforms**.** Tachycardia or extended PR interval with subsequent E/A fusion therefore prevents the measure of L-wave velocity. *Aortic regurgitation:* in cases of eccentric AR where regurgitant flow contaminates the assessment of transmitral forward flow, an L-wave velocity may not be clearly identified and may prevent a measurement from being made (Table 5).

**Table 5:** Table of the supplementary measures of LV diastolic function

Supplementary measures of LV diastolic function	
*Transmitral A wave duration*	
Measured by PW Doppler, the time from the onset of the A-wave to the end of flow at valve closure. A sample volume placed at the level of the mitral annulus provides clearer identification of A onset and cessation than a sample volume placed at the leaflet tips. The measurement is more easily made when the A-wave is clearly seen to start and end at the zero-velocity baseline. Identifying the onset of flow may be difficult when E and A are fused	
*Pulmonary vein flow*	
In the apical 4-chamber view, superior angulation of the transducer and use of colour flow will help locate the PV. The RUPV is usually easiest to identify and is adjacent to the atrial septum. If the signal is weak, manoeuvre the patient into a more supine position. Place the PW Doppler sample volume (1–3 mm volume) 1–2 cm into the RUPV Wall filter settings should be lowered (100–200 MHz). Sweep speed should be increased to > 100 mm/s. Ensure clear visualisation of the atrial reversal velocity waveform. Measurements should be averaged over 3 cardiac cycles, *at end expiration*. When two PV S peaks are present (S1 and S2), peak S2 should be measured for the S/D ratio	
*PV Ar duration*—measure by PW Doppler from the onset of the Ar wave to the end of flow. Optimised alignment with flow is usually best when sampling within the right PV adjacent to the inter-atrial septum. Low velocity reject/wall-filter should be adjusted to ensure that the onset/cessation of flow is clearly identified For the purpose of calculating the Ar-A, measurements of A and Ar duration should be made during cardiac cycles with similar R-R intervals. When the onset of either A or Ar flow is indistinct or there is E & A fusion, the preceding P-wave or QRS can be used as the starting reference point. Irrespective of the degree of impaired diastolic function, the onset of both A and Ar flow is simultaneous; it is the time-point at which each signal ends that identifies the difference in duration. Therefore, measuring from a common starting point to the end of each signal enables an estimation in the difference in duration between them. However, it is essential that R-R intervals are identical for both measures when applying this method	
*L-wave*	
The velocity of transmitral flow during the period of diastasis	

### Non-routine measures of diastolic function

Although the following parameters are not recommended for routine clinical practice, they may provide insight into LVDF in certain scenarios.

### Mitral annular tissue Doppler imaging (TDI)—e′/a′ ratio

In the normal heart, the TDI diastolic pattern at the mitral annulus mirrors that of transmitral blood-flow Doppler where the both E wave and e′ velocity are high, due to the predominance of rapid early diastolic filling, while the A wave and a′ are comparatively low. Therefore, high e′ velocities and relatively low a′ velocities result in an e′/a′ ratio that exceeds 1, is typically high in the young and fit, and increases with athletic conditioning and supra-normal early diastolic relaxation [65, 123, 124]. As expected, the age-related decrease in global LV relaxation rate leads to a continuous decline in peak e′ velocities with normal aging (Table 2). The age-related shift in early to late filling ratio is also reflected by a reversal of the e′/a′ ratio to less than 1 [125].

#### e′/a′—impaired diastolic function

With impaired LVDF, MV annular velocities and pattern reflect the decline in LV relaxation and shift in ratio between early and late LV filling. As such, when LV relaxation is impaired but LVFP and LAP remain normal, the onset of the e′ is subtly delayed [68, 126] and the velocity decreases, falling below that of a′ resulting in an e′/a′ ratio of < 1. Although decreased LV compliance and increased LVFP causes the transmitral blood flow E/A ratio to increase to above 1 (when not affected by pre-A velocity), this shift in ratio is secondary to an increase in E velocity due to elevated LAP and a reduction in A velocity due to increasing LA afterload and consequently reduced end-diastolic pressure difference between the LA and LV. Although MV annular velocities are affected by loading conditions and left heart pressures, they are much less load dependent than transmitral Doppler. Therefore, the absolute MV annular velocity continues to fall and the e′/a′ ratio remains < 1 when LVFP and LAP initially become elevated.

#### Limitation of e′/a′:

When LV filling becomes restrictive, significantly increased LA afterload leads to a marked reduction in LV filling from atrial contraction and although total filling volume is reduced, the majority of LV filling occurs in early diastole. Mitral annular velocities may reflect this physiology and explains why an e′/a′ ratio of > 1 may be seen in those with restrictive filling [127].

### IVRT

As described, in those with normal hearts, the IVRT is influenced by the rate of LV relaxation and therefore age. In the young and those who are athletic, rapid recoil and untwisting of the LV causes intracavity pressure to fall rapidly, resulting in a very short time between AV closure and MV opening and a consequently short IVRT (Table 5). With aging, the rate of myocardial relaxation slows with a corresponding increase in the rate of intra-cavity pressure fall and a consequent increase in the IVRT [58].

#### IVRT—impaired diastolic function

When LV relaxation is impaired but with normal compliance and normal LVFP, the slower rate of relaxation results in a slower rate of pressure decay within the LV and a consequently longer period between AV closure and MV opening. Although less accurate than direct measures of τ, the IVRT therefore reflects the rate of pressure decay and is increased when LV relaxation is impaired—similar to the physiology seen in normal aging hearts. When LVFP is elevated, the LV to LA diastolic pressure difference is reduced causing the MV to open sooner. Impaired LV diastolic function with elevated LAP therefore leads to shortening of the IVRT. For a healthy adult < 60 years old, a normal IVRT is typically < 80 ms. Impaired relaxation but with normal LAP results in a lengthened IVRT [128], typically > 100 ms. Conversely, decreased LV compliance with increased LAP results in an IVRT that shortens to a ‘normal’ range of 60–100 ms. When LV filling becomes restrictive and mean LAP is significantly raised, the MV opens sooner and the IVRT shortens further and may be as brief as 40–60 ms [129–131]. The relationship between impaired LVDF and the IVRT is therefore U-shaped, increasing in the early stages of diastolic impairment before decreasing as LAP increases (Table 6).

**Table 6:** Mean, standard deviation and lower/upper reference intervals for age-specific IVRT [51]

IVRT measures in normal hearts				
Age group	16–20	21–40	41–60	> 60
IVRT	50 ± 9 (32–68)	67 ± 8 (51–83)	74 ± 7 (60–88)	87 ± 7 (73–101)

#### Limitations of IVRT

*Short duration:* IVRT is typically very short in duration and is therefore subject to measurement error, particularly during periods of tachycardia, limiting its routine application. *Variations in pressure:* as the duration is entirely determined by pressure differences, the IVRT varies according to alterations in pressure either side of the MV. When LV systolic pressure is increased, LV relaxation will start from a higher pressure and therefore extend the time period between AV closure and MV opening, thus extending the IVRT [51]. Conversely, when SBP is low, relaxation starts from a lower pressure and IVRT will be shorter. IVRT is also affected by alterations in LAP and will therefore decrease in scenarios where significant MR or MS cause elevated LAP, irrespective of LV relaxation properties. Given the very short time-period being measured and the continuous nature of the variable, and therefore overlap in parameters between normal and abnormal filling, IVRT interpretation is most accurate and robust at the extreme ends of the spectrum where very long periods indicate slower LV relaxation while very short periods are likely to indicate elevated LAP.

### Velocity propagation (Vp)

Combining colour flow Doppler (CFD) with M-Mode (CMM) provides a spatiotemporal map of flow within the LV that is relatively independent of loading conditions and may be helpful for the assessment of LVDF. In the healthy heart, normal elastic recoil creates small mitral-to-apex pressure gradients that help generate the suction for early diastolic LV filling, causing blood to accelerate from the LA in early diastole and rapidly propagate to the apex [132–134]. This flow propagation velocity (Vp) can be mapped by CMM and measured as an indicator of LVDF. When diastolic function is normal, blood flow from base to apex is rapid with a Vp of ≥ 45 cm/s. Impaired relaxation leads to slower rate of pressure decay and therefore slower rate of early diastolic filling with a reduced Vp (< 45 cm/s). Vp is therefore an indirect measure of τ [135, 136].

#### E/Vp

Combining Vp (as a measure of LV relaxation) with peak E velocity (as an indicator of LAP) may be helpful for the assessment of LVFP. Although a direct estimation of LVFP is theoretically possible [135], the calculation is limited by inaccurate measures of the CMM slope for Vp estimation. However, utilising the E/Vp ratio may differentiate normal from raised PCWP (thereby LVFP) in both SR and AF [137]; an E/Vp ≥ 1.4 is suggestive of raised LVFP. In patients with impaired LV systolic function and low LVEF, an E/Vp ratio of > 2.5 predicts LVEDP of > 15 mmHg [71].

The E/Vp ratio may also hold prognostic value in the setting of HF. A ratio > 1.5 following acute myocardial infarction (MI) predicts in-hospital HF while a separate study found that a ratio of > 1.8 not only predicted HF in those with low or normal LVEF, but also correlated well (r = 0.73) with LV EDP [138]; in those with systolic HF, a ratio of > 2.7 predicted death, transplantation or HF hospitalisation [139].

#### Limitations of Vp and E/Vp

*Other determinants:* Vp is not solely determined by LV diastolic properties and is also affected by: LV geometry, ratio of MV orifice size to LV cavity size and by dyssynchronous relaxation [140–142]. The Vp slope may be erroneously normal in patients with impaired relaxation but where the LV cavity is small and hypertrophied. *Measurement reproducibility and standardisation:* Different measurement techniques exist (non-colour/colour flow interface *vs* slope of the first aliasing velocity) while interobserver variability has been reported to be as high as 20% [143]. Lack of standardisation limits the reproducibility of findings and hinders the comparison of studies, therefore restricting the application of results.

### Intracavity flow during the IVRT

In the normal healthy heart, there is very little movement of blood within the LV cavity during the IVRT. However, when abnormal and dyssynchronous relaxation is present, either due to LBBB or CAD, temporal differences in regional relaxation may create a pressure difference between the base and apex of the LV that results in intracavity flow during the IVRT. CW interrogates flow along the length of the LV cavity and is therefore the most suitable modality to identify and measure this abnormal flow. Flow is typically towards the apex with a velocity in the range of 20–60 cm/s, reaching 2 m/s in extreme cases. Although this parameter is not recommended for the routine assessment of LVDF, it’s presence may help identify abnormal LV relaxation, especially in the scenarios where LV dyssynchrony is present or expected.

### IVRT/T_E-e′_

**Fig. 15:**
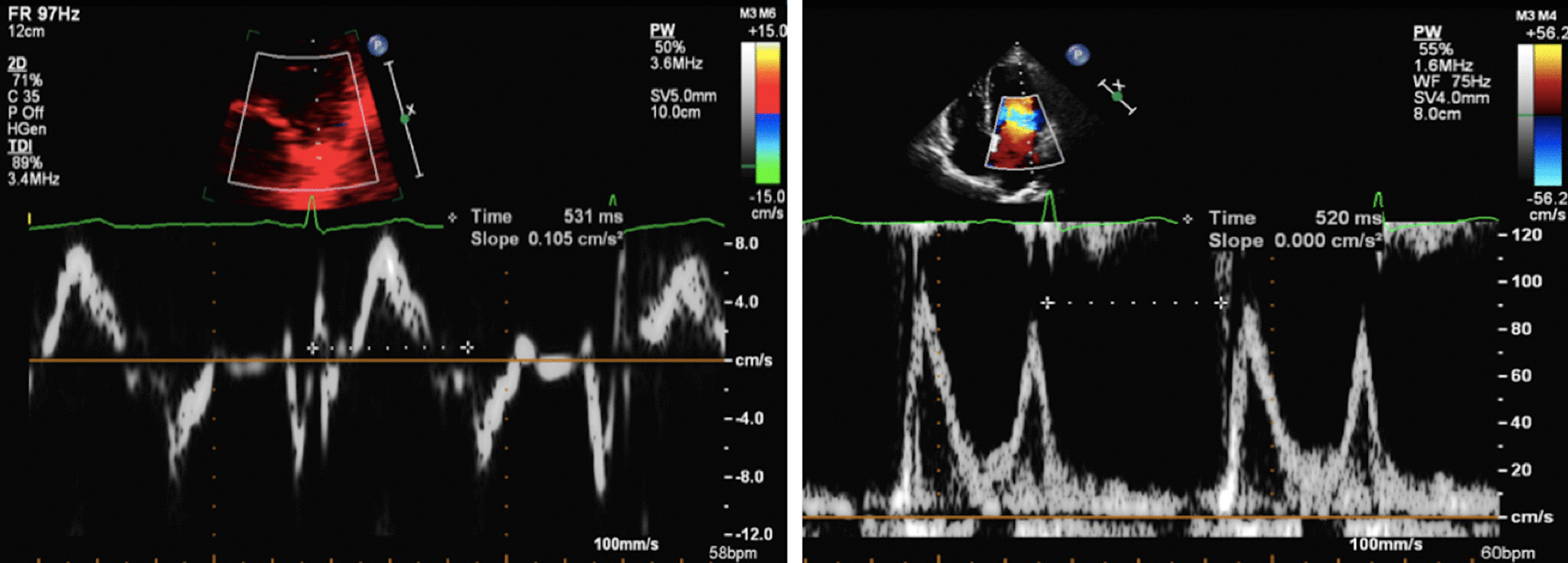
Measurement of the time difference between the onset of E and e′. Note the optimisation to decrease transit-time artefact and reduction of wall-filter/low-velocity reject to ensure signal onset is clearly seen

When diastolic function and LAP are normal, the onset of early myocardial relaxation and LV filling is almost simultaneous, with little to no measurable time difference between the onset of the E and e′ Doppler signals (Fig. 15) (Table 7). However, as impaired diastolic function with elevated filling pressures develops, slower LV relaxation causes a delay in the onset of e′ while increased LAP causes earlier opening of the MV and therefore earlier onset of the MV E wave. The time difference between the onset of the E and e′ waves therefore increases as diastolic function worsens. Measurement of this time difference (T_E-e′_) therefore relates directly with τ [140]. Combined with IVRT, to incorporate LAP, the IVRT/T_E-e′_ ratio can be calculated as an indicator of LVFP, with a value < 2 suggesting a PCWP of > 15 mmHg [144]. Because the IVRT/T_E-e′_ considers the timing of e′ onset relative to the IVRT and E signal onset, this measure can be applied for the assessment of LVDF in those with significant MV disease [144]; LV diastolic pressures are likely raised when the ratio is < 4.2 in those with MS and < 5.6 in those with significant MR [145].

**Table 7:** Table of the non-routine measures of LV diastolic function

Non-routine measures of LV diastolic function	
*IVRT*	
IVRT can be measured using either PW or CW Doppler. From an apical 3 or 5-chamber view, the sample volume or cursor is moved from the mitral leaflet tips toward the left ventricular outflow tract until it captures both the completion of aortic ejection and the onset of mitral inflow. NB, the low-velocity reject/wall filter should be adjusted to ensure the end of aortic and onset of mitral flow can be clearly identified IVRT can also be measured from a TDI trace, although this requires multiple (ideally four) annular measurements to average the significant regional variation between annular points measured. The IVRT is longer in the anteroseptal wall compared with the lateral wall and longer in ischaemic segments [131]. The interval begins at the cessation of the S-wave and ends with the start of the e′ wave	
*Mitral annular tissue-Doppler imaging—e′/a′ ratio*	
Peak modal velocity at the leading edge of the spectral waveform in early diastole (after the T-wave). Gain and reject settings should be optimised to display high amplitude annular velocities with reduced clearly defined modal waveform. Measurements should be averaged over 3 cardiac cycles, at end expiration	
*Pulmonary vein S and D wave deceleration times*	
The PVS and PVD deceleration slopes are measured from the peak velocity of the jet to the end of flow. Greater PW Doppler scale will enlarge the spectral waveform and increase measurement accuracy	
*Vp*	
Acquisition is performed in the A4C. A narrow CFD sector should extend across the mitral valve and at least 4 cm into the LV with the M-mode cursor carefully aligned with the direction of blood flow at a sweep speed of > 100 cm/s. The Nyquist limit is adjusted to around 40–50% of the peak E velocity to generate aliasing of the higher velocity flow at the centre of the blood flow column [148]. The flow propagation slope is measured	
*IVRT/T*_*E-e′*_	
When measuring IVRT/T_E-e′_, both waveforms are acquired as per described previously but at a faster sweep-speed of 100 mm/s and greater spectral Doppler scale to improve temporal resolution and increase measurement accuracy of these short time-periods. The time is measured between the peak R-wave on the ECG to the onset of both the E and e′ waves. Due to the non-simultaneous method, R-R intervals should be matched. Low gain and wall-filter settings should be optimised to provide waveforms with clear onset. The e′ should be measured at the four annular sites from the A4 and A2C views and an average time calculated. The ratio of IVRT to T_E-e′_ indexes the measure to LV relaxation time and can be useful in borderline cases or significant mitral valve disease; a ratio < 2 suggest holds high sensitivity and specificity for raised LVFP [144, 145]	
*A-wave transit time*	
When both the transmitral E/A and Er/Ar waves are identifiable on the same Doppler trace, a simple measure of the time to onset or time to peak velocity can be made. If it is not possible to identify both signals on the same spectral Doppler display, individual time measures can be made using the ECG R wave as the starting reference point and the time difference established—similar R-R intervals is essential for the accuracy of this measure	

### A-wave transit time

**Fig. 16:**
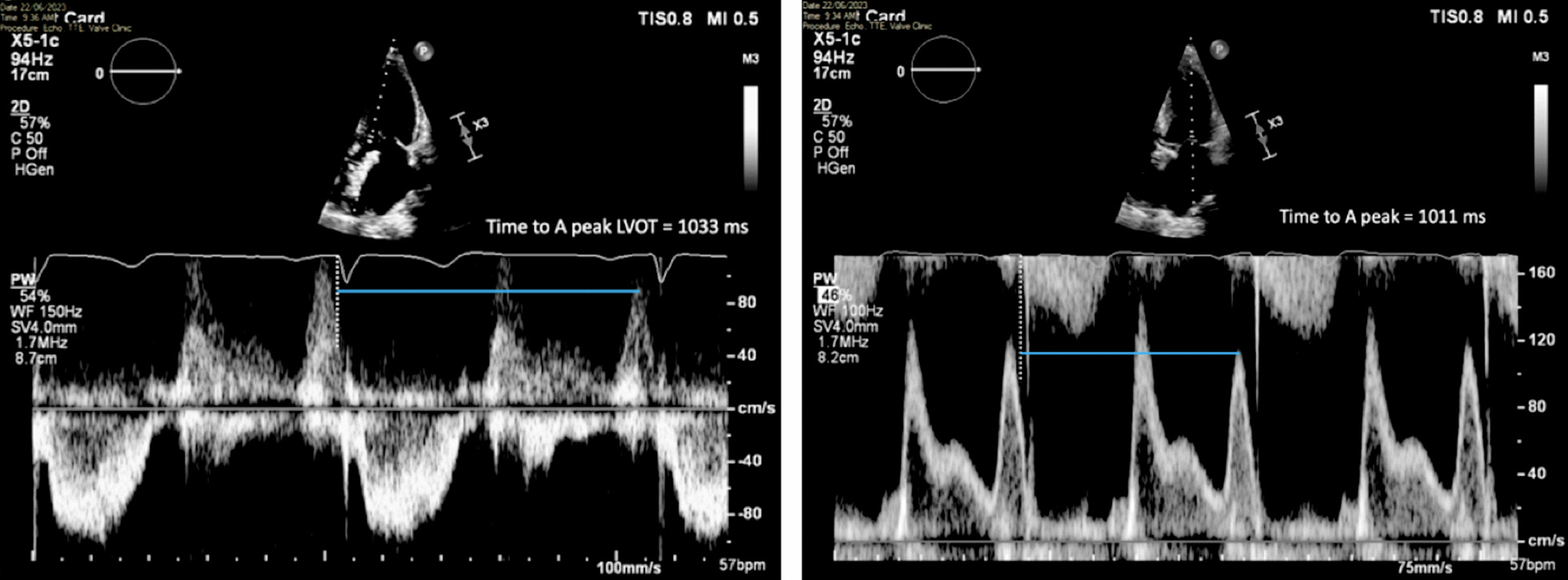
Measurement of A-wave transit time. The time-to-peak A wave has been measured in this case with the R-wave defined as the starting point. The short transit time of 22 ms is suggestive of decreased LV compliance

Flow entering the LV during early and late diastole causes a movement of blood towards the AV that is detectable within the LVOT by Doppler imaging, described as Er and Ar waves (Fig. 16). These waves do not occur instantaneously with transmitral forward flow, however, and a time-delay exists between LV inflow and the onset of the Er and Ar within the LVOT. This time-delay is referred to as the Er or Ar transit-time with the Ar transit-time being typically shorter than that of the Er (Table 7).

In patients with impaired diastolic function and increased myocardial stiffness, the A wave propagates rapidly through the LV with a consequently short Ar transit time. A peak-to-peak transit time of ≤ 45 ms or onset-to-onset transit time of ≤ 50 ms suggests decreased LV compliance and increased end-diastolic stiffness, consistent with impaired diastolic function [146, 147].

### Other indicators of impaired LV relaxation

The parameters described below reflect global myocardial function and are therefore considered indicative of impaired LV relaxation when they fall below the normal expected range.

### Impaired myocardial function – LVEF and strain analysis

There are certain clinical scenarios in which impaired LV relaxation can be assumed to be present. Impaired systolic contractility identifies impaired myocardial function and indicates that LV relaxation is likewise impaired. Therefore, an LVEF of < 50% or absolute GLS < 16% [149] is consistent with impaired LVDF; an assessment of LVFP should then be made. Additionally, impaired relaxation should also be assumed in those with left ventricular hypertrophy, regional wall motion abnormalities or known myocardial disease of other aetiology. However, reduced GLS or LVEF are not ubiquitous amongst those with impaired diastolic function and may be within the normal range despite diastolic impairment and raised LVFP. As such, LVEF ≥ 50% or absolute GLS ≥ 16% do not confirm normal diastolic function and wider consideration of secondary parameters may be required. In athletically fit individuals, physiological adaptation of the heart may result in low-normal parameters of systolic contraction (LVEF and GLS) at rest as the larger ‘athletes’ heart’ is able to deliver the required stroke volume and cardiac output at a lower magnitude of contractility. In this scenario, normal or supra-normal diastolic parameters usually help to differentiate normal athletic adaptation from myocardial impairment [42]. However, if parameters of both systolic and diastolic function are at the lower limits of normal, further imaging (incorporating exercise testing) may be required to confirm normality/pathology.

### Algorithms for the assessment of LV diastolic function

#### Normal systolic function

##### Normal versus impaired systolic function

Although previous diastolic guidelines provided recommendations that applied to all patients, the diastolic assessment is simplified by consideration of key parameters of LV systolic function. When LVEF is < 50%, GLS < 16% or the presence of myocardial disease is confirmed (ischaemic heart disease with regional wall motion abnormalities, cardiomyopathy, pathological LVH), abnormal relaxation is extremely likely and therefore assumed—the focus of the diastolic assessment is to investigate whether LVFP are consequently elevated. However, when LV systolic function is normal, relaxation must be investigated along with LVFP. Furthermore, the accuracy of LVDF parameters to identify elevated LVFP is greatest when LV systolic function is impaired. The process of investigating LVDF is therefore subtly but necessarily different between those with normal LV systolic function and those with confirmed abnormal systolic function or known myocardial disease.

#### Normal systolic function algorithm

Systolic function is considered impaired when either LVEF is < 50% or absolute GLS < 16%, whereas LVEF ≥ 55% and GLS ≥ 18% are consistent with normal systolic function. There exists, therefore, a grey-zone of LVEF (50–54%) and GLS (16.0–17.9%) where analysis of systolic function requires interpretation of other factors, including: LV volumes and TDI, prior echo reports, clinical history, cardiovascular symptoms, family history, and potentially functional assessment [86]. Consequently, LVEF and GLS cannot provide binary normal/abnormal cut-offs for systolic function. As such, the LVDF algorithm for those with normal systolic function cannot be defined by specific LVEF or GLS ranges. Instead, confirmation of normal systolic function should be made by global assessment and in accordance with the previously published guidance [86].

**Fig. 17:**
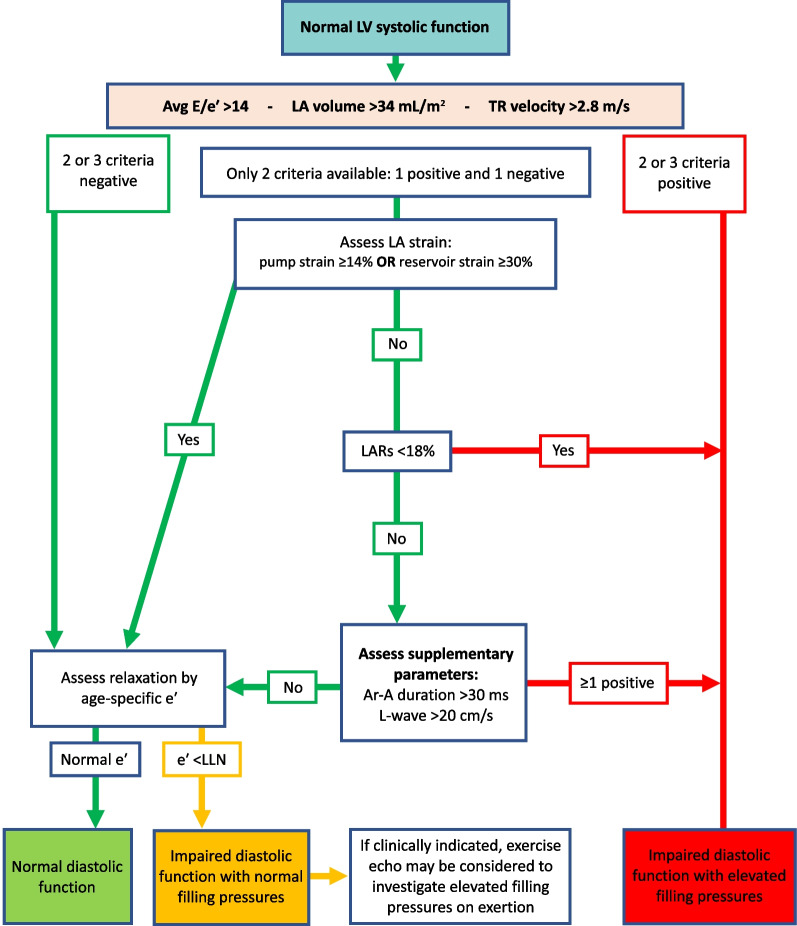
Algorithm for the assessment of LVDF in those with normal systolic function. It is recommended that the algorithm is not applied in the following conditions: severe MR/MS or MAC or MV replacement or repair

In those with normal systolic function and no evidence of myocardial disease, there exists significant overlap between normal and abnormal for parameters of LV relaxation. As such, the assessment of LVDF in those with normal systolic function must start with the assessment of LVFP (Fig. 17). This assessment centres on three key variables: E/e′ > 14, TR velocity > 2.8 m/s and indexed LA volume > 34 mL/m^2^. When two or three of these parameters are negative (ie, below the referenced cut-off), LVFP are considered normal. If two or three parameters are positive (ie, above the referenced cut-off), diastolic function is impaired and LVFP are elevated.

When only two criteria are available, typically in the absence of TR, with one positive and one negative, the missing parameter can be replaced by LAs. In those with normal systolic function, high-normal values of LARs (> 30%) and LAPs (> 14%) indicate normal LVFP with very high accuracy [103]; LV relaxation should then be reported according to age-specific e′. As diastolic impairment progresses and LVFP increase, parameters of LA function typically deteriorate. When replacing a missing key parameter, LARs < 18% is suggestive of elevated LVFP [103]. However, when systolic function is normal, there is overlap in LAs parameters across normal and elevated LVFP, such that a range of LARs > 18% is seen in both normal diastolic function as well as impaired diastolic function with elevated LVFP [103]. The accuracy of each parameter to identify elevated LVFP is therefore weaker when systolic function is normal and further analysis of diastolic parameters is therefore required when LARs falls within a range of 19–29%. In this setting, the supplementary parameters of Ar-A duration > 30 ms and L-wave velocity > 20 cm/s correlate well with elevation of LVFP and should therefore be considered. If one or both of these parameters are positive, the diagnosis of impaired diastolic function with elevated LVFP is made. If not, LVFP are considered normal and LV relaxation is reported according to age-specific e′.

Importantly, replacement of the missing parameter does not improve the accuracy of the algorithm. However, because TR is absent in a significant number of patients (between 40 and 60% in some studies [150, 151]), replacement with LAs analysis improves algorithm feasibility to around 95% and reduces the number of indeterminate outcomes [104]. Feasibility studies have reported that LA strain can be measured in between 92–95% of patients [103]. However, in those with one positive and one negative key variable and where LA strain analysis is not possible, the assessment of LVFP is indeterminate and LV relaxation is reported according to age-specific e′, although the supplementary parameters described within the algorithm may provide some insight into LVFP.

#### Impaired systolic function or known myocardial disease algorithm

**Fig. 18:**
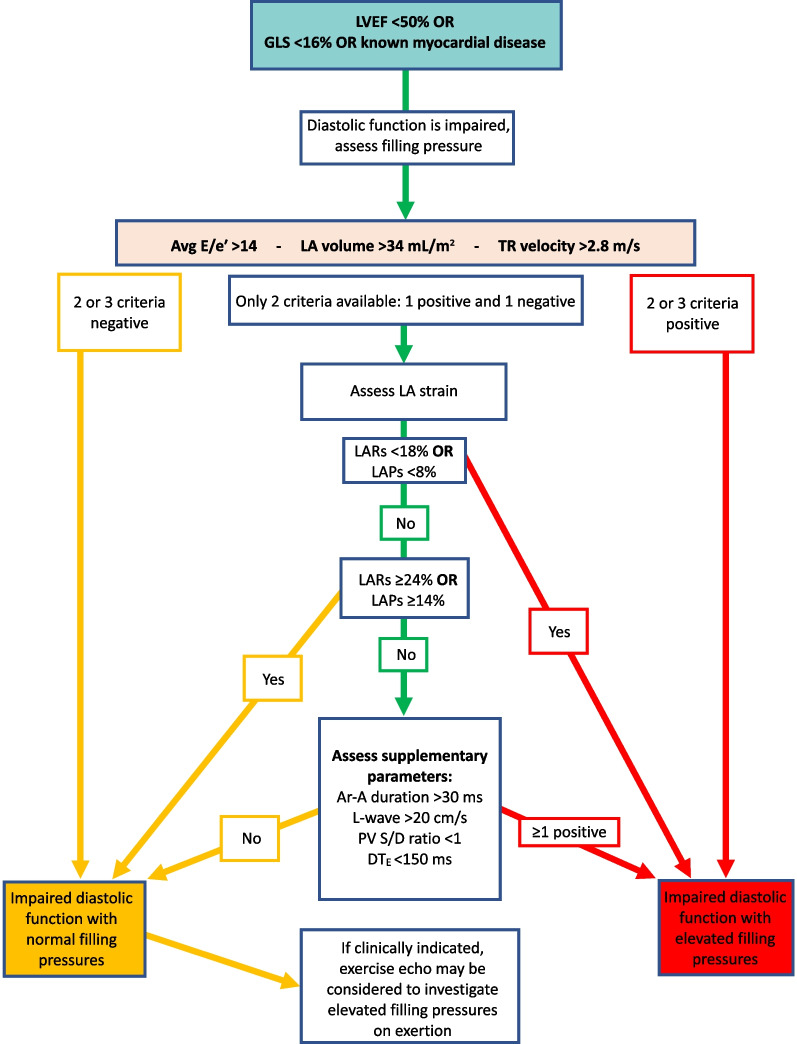
Algorithm for the assessment of LVDF in those with impaired systolic function or known myocardial disease. It is recommended that the algorithm is not applied in the following conditions: LBBB, RV apical pacing or resynchronisation pacing (CRT); LV assist devices

When LV systolic function is impaired or known myocardial disease is present, LV relaxation is almost certainly impaired—the focus of the diastolic assessment is therefore to determine whether LVFP are elevated using the three key variables of LVFP (Fig. 18). When two or three of these parameters are negative, LVFP are considered normal. If two or three parameters are positive, diastolic function is impaired and LVFP are elevated. When only two criteria are available with one positive and one negative, the missing parameter can be replaced by LAs. However, the cut-offs are subtly different to those with normal LVEF. In those with impaired systolic function, absolute LAPs < 8% or LARs < 18% indicate elevated LVFP while ≥ 14% and ≥ 24% indicate normal LVFP with high accuracy. Due to the overlap in LA strain parameters between normal and elevated LVFP, LAPs values of 8–13% and LARs 18–23% may be seen in the settings of both normal and elevated LVFP [103] and supplementary parameters should be considered. In addition to Ar-A duration > 30 ms and L-wave velocity > 20 cm/s, a PV S/D ratio < 1 and MV E deceleration time < 150 ms are accurate indicators of elevated LVFP with high sensitivity and specificity when LV systolic function is impaired. If one or more of these supplementary parameters are positive, elevated LVFP should be reported. If all are negative, LV relaxation is impaired but LVFP are normal. As impaired relaxation has been established, age-specific e′ are not considered in those with impaired systolic function or known myocardial disease. In those with one positive and one negative key variable but LA strain analysis is not possible, the assessment of LVFP cannot be performed and LV relaxation is therefore impaired but with indeterminate LVFP. However, the supplementary parameters described within the algorithm may provide some insight into LVFP.

#### Atrial fibrillation algorithm

Although the assessment of LVDF is complicated by cardiac arrhythmia, AF is a common finding in those with impaired diastolic function (AF being commonly caused by abnormalities of LV structure and/or function) and often seen in patients with HF; the echocardiographic investigation of diastolic function should not be omitted because of the presence of AF alone. However, because it is not possible for all measures of diastolic function to be made during the same cardiac cycle, the R-R variability, and therefore beat-to-beat variation of loading conditions that is characteristic of AF risks invalidating the assessment of diastolic function if appropriate measurement methods are not followed. The writing group recommend that if the HR is < 100 bpm (ideally < 90 bpm), Doppler parameters can be obtained from a single beat if the two preceding R-R intervals are of similar duration (within 60 ms of one another). When deriving the E/e′ ratio, it is important that the E velocity and the e′ velocity are measured from cardiac cycles that are similar length [107].

The algorithm for the assessment of LV diastolic function in those with AF incorporates parameters that when combined provide an overall accuracy of 75% for the detection of elevated filling pressures in those in AF [152]; the assessment of LV relaxation cannot be performed accurately in AF. This algorithm should be used in *all *patients in AF, irrespective of LV systolic function.

**Fig. 19:**
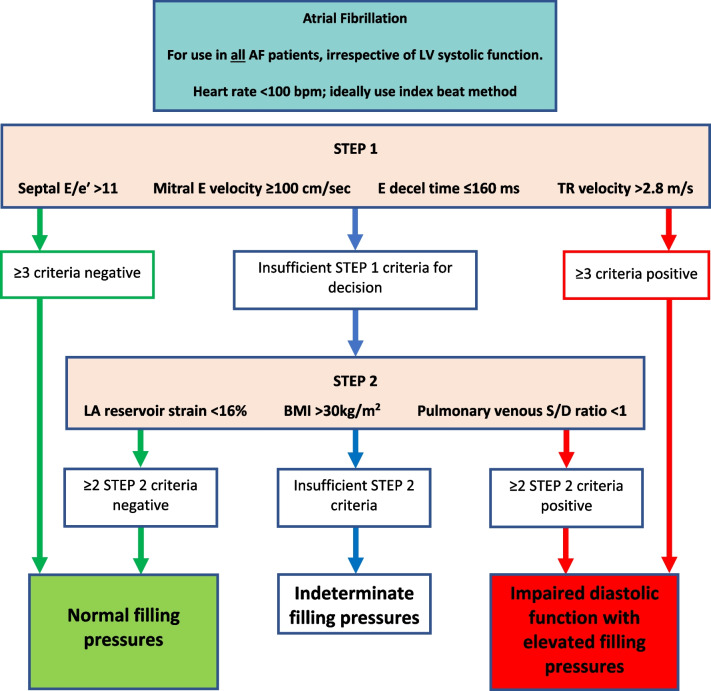
Algorithm for the estimation of LVFP in patients with AF. None of the parameters listed in the algorithm are of sufficient accuracy to be considered adequate stand-alone measures for the assessment of LVDF. The algorithm is not recommended for application in the following conditions: complex congenital heart disease, cardiac transplants, end-stage liver disease, mitral stenosis or mitral annular calcification resulting in significant mitral stenosis, prosthetic mitral valve, severe aortic stenosis, severe mitral or tricuspid regurgitation, and atrial fibrillation with rapid average ventricular rate at rest (> 120 bpm) [152]

Step 1 incorporates four key variables: septal E/e′, MV E velocity, MV E deceleration time, and TR velocity. When ≥ 3 parameters are positive, LVFP are considered elevated. When ≤ 3 are negative, LVFP are considered normal. In scenarios where ≤ 3 parameters are available and only ≤ 2 are positive or negative, filling pressures are unclassified and further supplementary parameters should be considered in Step 2: LARs, PV S/D ratio and BMI. When ≥ 2 of these 3 are positive, filling pressures are considered elevated. When ≤ 2 of 3 are negative, filling pressures are considered normal. If these criteria in STEP 2 are not met, LV filling pressures are indeterminate (Fig. 19).

#### Septal wall E/e′

In the setting of AF, greater variability of the R-R interval results in greater variation of the lateral e′ velocity than that of septal e′. It is therefore recommended that the E/e′ ratio is calculated using septal wall e′ alone. When considered within the AF algorithm, a ratio of > 11 provides greatest accuracy for detecting elevated LVFP.

#### Mitral E peak velocity ≥ 100 cm/s and deceleration time ≤ 160 ms

The transmitral peak E velocity is reflective of the early diastolic pressure difference between the LA and LV. When LV diastolic function is normal, rapid and efficient LV relaxation generates a suction effect that increases the LA-LV pressure gradient without elevation of LAP. When LV diastolic function is impaired with elevated LVFP, increased LAP causes MV E velocity to increase while decreased LV compliance causes rapid equalisation of LV and LAP, resulting in rapid deceleration of flow velocity. As the most common cause of AF is an abnormality of LV structure and or function [153], an E velocity > 100 cm/s and deceleration time ≤ 160 ms in this setting are therefore supportive of impaired LVDF with consequently elevated LVFP and LAP.

#### TR velocity > 2.8 m/s

Although the cut-off for TR velocity remains the same as in SR at > 2.8 m/s, care should be taken to avoid measurement following short R-R intervals that may be associated with underestimated peak TR velocity.

#### Obesity—body-mass index (BMI) > 30

The relationship between obesity and HFpEF has become clearer in recent years. Rather than merely a mechanical cause of dyspnoea that is associated with comorbidities for HFpEF (hypertension (HTN), diabetes mellitus (DM), CAD etc.), the metabolic consequences of obesity have system wide effects on the cardiovascular system that lead to systemic inflammation, mitochondrial dysfunction, autonomic dysregulation and altered haemodynamic loads. In turn, causing abnormal myocardial structure, function and metabolism that is the basis for the independent relationship between obesity and HFpEF that is not explainable by the associated cardiovascular risk factors alone [154]. Given the relationship between obesity and HFpEF and that AF is a recognised consequence of chronically elevated LVFP (the haemodynamic correlate of HFpEF), a BMI of > 30 is therefore considered supportive of elevated LVFP in the setting of AF.

#### LARs

Irrespective of LVFP, due to the absence of LA contraction/pump phase, parameters of LARs are routinely lower in AF than in SR [97]. Consequently, the cut-off for identifying elevated LVFP in AF is lower at < 16% [152].

#### PV S/D ratio

As described, decreased LA compliance (common in AF) and increased LAP causes a reduction in PV D velocity, leading to a reduction in the PV S/D ratio to below 1.

### Assessment of diastolic function in special groups

### Valvular disease

#### Mitral stenosis

In those with moderate or severe MS, under filling of the LV results in normal or even low LV diastolic pressure while MV obstruction increases LAP, causing the E velocity, E/A ratio, E/e′, LA volume and SPAP to increase; if MS is calcific in nature and extends into the MV annulus, e′ velocities may also decrease. Although impaired LV diastolic function is not a typical finding in those with rheumatic MS, when moderate or severe MS is present the transmitral blood flow velocity and MV annular relaxation velocities are predominantly determined by the severity and extent of MV disease rather than diastolic properties of the LV; however, because even mild MS can lead to increased transmitral E velocity and alter the E/A and E/e′ ratios, the diastolic assessment should incorporate absolute e′, TR velocity, LA volume and parameters of LV geometry and function (including mass and GLS) in order to make a global judgement of diastolic function and the likelihood of impairment. Ultimately, it may not be possible to determine LVDF by echocardiography in the presence of moderate or severe MS and invasive catheterisation procedures may be required. Understanding the clinical presentation of the patient to identify the likelihood of LV myocardial impairment (DM, HTN, CAD etc.) can help determine whether impaired diastolic function is likely.

Although both IVRT < 60 ms and MV A velocity > 1.5 m/s suggest raised PCWP in those with MS [145], this does not differentiate the cause. By incorporating the time difference between the onset of MV annular motion and blood-flow, IVRT/T_E-e′_ may identify impaired relaxation. Although parameters of LV systolic function are likely affected by LV under filling in those with moderate or severe MS, low GLS may help identify abnormal myocardial function when other parameters of diastolic function cannot be interpreted. AF is common in those with severe MS and should be considered when reporting GLS and LVDF. Markers suggestive of impaired relaxation—IVRT/T_E-e′_ < 4.2

#### Mitral regurgitation

When the LV and LA are compliant, moderate or severe primary MR results in chamber dilation that negates an increase in LAP. However, once the regurgitant volume exceeds the compliance capacity of the LA to accommodate the additional volume loading, LAP becomes elevated, leading to increased E velocity, E/A ratio, E/e′, LA volume and SPAP; significant MR may also lower or completely diminish PV S velocity. Even when LAP is normal and MR moderate, the additional regurgitant volume increases the early diastolic transmitral volume and may elevate E velocity, causing the E/A and E/e′ ratios to increase and PV S velocity to reduce. The increase in LAP and forward flow volume caused by moderate or severe primary MR causes an elevation in E velocity that consequently confounds the assessment of LVDF. Although the below markers may be of some utility in identifying elevated LAP, it may not be possible to differentiate the cause of raised LVFP or accurately assess LVDF in the presence of moderate or severe primary MR. LV diastolic pressure measurements may require cardiac catheterisation if impaired diastolic function is suspected and of clinical importance. In patients with severe MR secondary to LV disease, the parameters of LVFP and LAP reflect the combination of disease processes.

*Markers of raised LAP:* IVRT < 60 ms, Average E/e′ > 14 (only if impaired LV systolic function), Ar–A ≥ 30 ms, IVRT/T_E-e′_ < 5.6 if normal LVEF (more specific if < 3). GLS is affected less by loading conditions than LVEF and may help identify abnormal myocardial function in the setting of severe primary MR.

#### Mitral annular calcification

In the presence of mitral annular calcification (MAC) and mild calcific MS, the assessment of LVDF can be challenging. Absolute e′ velocities may be reduced due to tethering of the MV annulus and consequently reduced relaxation velocities, while E velocity may be increased due to accelerated flow through the decreased mitral valve area. E/e′, E/A ratio, and LA size may all increase secondary to MAC. However, MAC is commonly associated with hypertensive heart disease, chronic kidney disease and CAD and is therefore seen in patients with impaired diastolic function. Consideration of timings may be helpful alongside assessment of the degree of valvular obstruction. For instance, if no significant obstruction is present but the IVRT is 20 ms less than expected for age, LAP may be increased. Conversely, if the E wave is increased but the IVRT is within normal range, then E velocity is likely raised due to the decreased MVA. In the absence of pulmonary disease, elevated TR velocity may suggest increased LAP. Given the comorbidities associated with MAC, a simple estimate of E/A can help differentiate normal LAP (< 0.8) from raised LAP (> 1.8); for those with an intermediate ratio (0.8–1.8), an IVRT of < 80 ms suggests raised LAP [155].

#### MV repair/replacement

Assessment of LV relaxation and LVFP is difficult following mitral valve surgery. Decreased MV orifice area will lead to increased transmitral flow velocity while annular velocities are very likely to be reduced due to the presence of an annuloplasty ring or replacement valve—LA volume will very likely be increased and function possibly reduced due to previously severe regurgitation or stenosis. As with MS/MR, TR velocity and flow timings (IVRT and IVRT/TE-e′) may be of value alongside LV GLS and understanding the clinical background to determine the likelihood of impaired diastolic function.

#### Aortic regurgitation

Chronic severe AR is well tolerated in those with a distensible and compliant LV (typically young individuals with bicuspid aortic valve because the additional diastolic filling is matched by an increase in LV size to maintain low diastolic pressure [156]). Once the AR volume exceeds the capacity for LV preload to adapt, LV diastolic pressure will rise progressively throughout diastole [157–159] leading to increased EDP and therefore LA afterload with consequently low A velocity with increased E/A ratio; the E deceleration time may also decrease as LV diastolic pressure rises more rapidly. In chronic AR, a combination of increased E/e′, LA volume and TR velocity are suggestive of raised LAP.

When severe AR is acute, the LV is not instantaneously distensible to accommodate the sudden onset of significant additional diastolic volume. The inability to distend renders the LV incompliant with diastolic pressures becoming markedly elevated [160]. This rapid increase in LV diastolic pressure may cause early closure of the MV and an abbreviated filling period with potentially some degree of diastolic MR due to significantly raised LV EDP [161]. However, these findings merely reflect the haemodynamic consequence of sudden onset severe AR overloading an LV that may be entirely normal. Although acute severe AR may prevent an accurate assessment of intrinsic myocardial diastolic function, it is poorly tolerated and the detailed assessment of LV relaxation is unlikely to be of immediate clinical importance.

Intrinsic diastolic impairment and decreased compliance limit the capacity of the LV to dilate in the face of volume overload. A normal end-diastolic volume in the setting of severe chronic AR may therefore be suggestive of decreased LV compliance, especially when combined with parameters suggestive of raised LV EDP.

### Cardiomyopathies

#### Hypertrophic cardiomyopathy (HCM)

Due to increased LV mass, reduced chamber compliance, microvascular ischaemia and myocardial fibrosis, impaired diastolic function is common in HCM and results in elevated LVFP and LA dilatation [162, 163]. Accurate classification of diastolic function severity is essential for appropriate therapy decisions, yet challenging in HCM due to the concomitant presence of LVOTO and MR in many patients. Many independent echo variables have weak correlations with LVFP, including E/e′ [164]. As such, integration of several parameters is necessary to quantify diastolic function accurately [165]. For appropriate medical management and identification of those who may be suitable for advanced heart failure therapy, it is essential to identify patients with preserved LVEF but a restrictive diastolic filling pattern [166]. The algorithmic approach to assessing LVDF in the setting of myocardial disease should be undertaken in those with HCM [167], although LA volume and function should be interpreted in caution when moderate or severe MR is present.

#### Restrictive cardiomyopathy

This group of cardiomyopathies of variable aetiology have been described according to their impact on LV filling. Unsurprisingly, therefore, some degree of diastolic impairment is expected in those with infiltrative and storage disorders that fall into the category of a restrictive cardiomyopathy. The standard algorithm for assessing diastolic function remains valid in this group. Although diastolic function may be markedly abnormal in the chronic stages of these processes, impairment may be subtle in the early stages with slower relaxation but normal LVFP. A restrictive filling pattern can be found in a number of disease groups however (ischaemia, valvular disease, HCM, DCM) and the aetiology of the underlying myocardial disease may not be identifiable from the echocardiogram alone [168].

### Arrhythmia

#### Sinus tachycardia

Measurement of timings, especially those of brief duration, will be challenging during sinus tachycardia. A sweep-speed of 100 mm/s should be utilised. Fusion of the transmitral E and A waves makes assessment of E velocity and E/A ratio difficult or may even prevent measurement. Although it is possible to perform measures of diastolic function during the compensatory pauses that follow ectopics, the effect of persistent tachycardia on cardiac loading conditions should be considered and how this might affect LVFP and therefore echocardiographic parameters of diastolic function. Although an accurate assessment of LV relaxation may not be possible, the following parameters and associated cut-off values are suggestive of raised LVFP:

*Markers of raised LVFP:* average E/e′ (> 14 provides highest specificity, > 10 is more sensitive but less specific), PV systolic filling fraction ≤ 40% (if good tracings possible), IVRT ≤ 70 ms, TR velocity > 2.8 m/s, E/A > 1 in patient with LVEF < 50% (if pre-A velocity < 20 cm/s).

#### AV block, LBBB and paced rhythms

A long PR interval, LBBB or ventricular pacing may result in fused E/A waves, causing elevated pre-A velocity. A very long PR interval (> 320 ms) or similar paced AV delay will result in marked E/A fusion and potentially diastolic MR. E/A fusion increases atrial pump SV and may result in a longer A wave duration, altering the Ar-A duration, and higher pulmonary venous systolic velocity leading to altered PV S/D ratio. If the fusion is minimal, grading of diastolic function may still be possible according to the standard algorithm. Decreased IVRT may help identify raised LAP.

*Markers of raised LVFP:* average E/e′ > 14, LA volume > 34 mL/m^2^, Ar-A duration > 30 ms (if E/A not fused), TR velocity > 2.8 m/s.

### Pulmonary arterial hypertension - differentiating pre and post-capillary PH

**Fig. 20:**
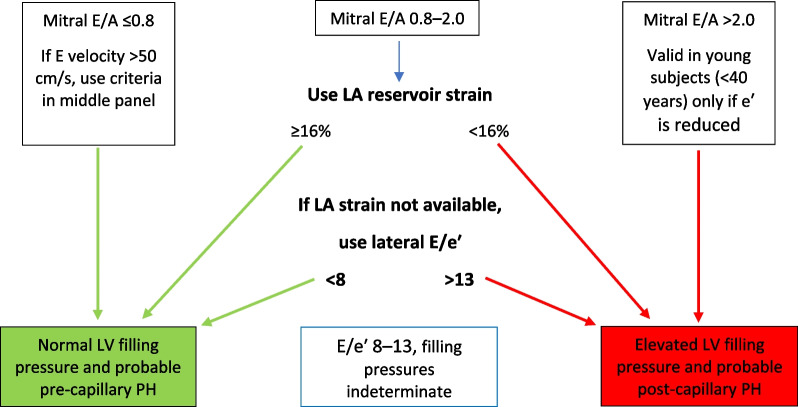
Algorithm for differentiating pre and post-capillary PH—reproduced with permission [170]. Accuracy to differentiate between normal and elevated LV filling pressure: Mitral E/A and LA reservoir strain: 85% accuracy. Mitral E/A and lateral E/e′ < 8 or > 13: 86% accuracy

In the absence of lung disease, raised LVFP translates to raised PCWP and consequently raised SPAP, causing PH. A TR velocity > 2.8 m/s is therefore an important indicator of impaired diastolic function. However, PH may occur for reasons other than abnormal LV diastolic function. Distinguishing pre-capillary PH (pulmonary vascular remodelling) from post-capillary PH (left-heart disease) has important prognostic and therapeutic implications. The definition of pre-capillary PH requires the PCWP to be ≤ 15 mmHg [169]. Thus, identifying elevated LVFP effectively excludes the diagnosis of pre-capillary PH (although occasionally some patients may have both conditions). Differentiating which patients have pre-capillary versus post-capillary PH (or both) is challenging. Markers suggesting that PH may be *pre-capillary* [76, 88] and *not* due to LVDF and elevated LVFP are listed:

Fixed dilated inferior vena cava, LV eccentricity index > 1.2, RV > LV size, RV dysfunction, E/e′ < 10 (if significant septal flattening, use lateral e′ only), normal LA size and function, mid-systolic notch in right ventricular outflow tract, PW Doppler or pulmonary valve Acceleration Time < 80 ms and medical condition associated with PH. However, by combining transmitral E/A ratio, LA reservoir strain and lateral E/e′, LVFP can be investigated as a potential cause of PH (Fig. 20) [170].

### Heart transplantation

Before diastolic assessment is made, it is essential to consider the parameters being measured in the context of the heart age, rather than the age of the recipient patient. Donor hearts are commonly those of healthy and young individuals and may present with very different diastolic flow profiles to those expected of an older recipient patient. Bi-atrial surgery will result in enlarged atria with clearly abnormal atrial function. Bicaval surgery may not affect left atrial function. Reduced atrial contraction often leads to a reduced A wave velocity and consequently abnormally raised E/A ratio. Reduced e′ velocities due to bi-atrial surgery may lead to abnormally raised E/e′ [170]. A tachycardia is commonly seen despite normal systolic and diastolic function. If bi-atrial surgery has been performed, competing atrial signals may cause discordant atrial contractions, significantly altering transmitral flow profiles. The only reliable marker of raised LVFP may be a raised SPAP in the absence of pulmonary disease.

### Provocative manoeuvres to unmask increased LVFP

#### Diastolic stress-echocardiography

##### Indications for diastolic stress-echocardiography

In patients with symptoms of exertional dyspnoea of unknown cause and impaired relaxation but normal LVFP at rest and no other identifiable cause on resting echocardiography, exercise stress echocardiography (ESE) for the assessment of diastolic function can be considered [171].

#### Diastolic function during exercise

The normal heart is able to increase SV and CO without a significant increase in LVFP. During exercise, augmented elastic recoil and relaxation causes the minimum diastolic pressure to fall, generating a greater transmitral pressure gradient that enhances early diastolic suction and facilitates greater LV filling without an increase in LAP [172–174]. These acute physiological adaptions to exercise are observed echocardiographically as an increase in septal and lateral annular velocities (augmented LV relaxation) and an increase in the E velocity (greater transmitral gradient). As the increase in both E wave and MV annular velocities is roughly proportional, the E/e′ ratio is not elevated by exercise in the normal heart and remains relatively unchanged throughout the test [175]. Excluding exercise induced ischaemia, increasing HR does not induce impaired diastolic function. In those with impaired diastolic function, the relaxation response is attenuated during exercise so that LV minimal pressure does not decrease significantly and the greater transmitral pressure gradient required to augment LV filling volume is achieved by an increase in LAP [172]. Peak E velocity increases proportionally with LAP while impaired myocardial relaxation results in persistently low e′ velocities throughout exercise, thus leading to an increased E/e′ ratio. With rising LAP, elevated SPAP causes TR velocity to increase [85].

An assessment of diastolic function during ESE is therefore indicated in those experiencing exertional symptoms and whose resting echo demonstrates impaired relaxation with normal LVFP (ESE is very unlikely to reveal elevated LVFP during exercise when resting LV diastolic function is entirely normal). When LVFP are raised at rest, diastolic-specific ESE does not add clinical benefit as the likely cause of exertional symptoms has been identified by resting TTE. However, if performed for the investigation of myocardial ischaemia, correlating patient symptoms with diastolic and systolic function may be beneficial [173].

#### Performing a diastolic ESE—exercise protocol

As pharmacological agents (Dobutamine, Adenosine, Atropine etc.) do not provide the same level of physiological stress as physical exercise and do not generate the same degree of venous return, the diastolic stress test is performed using either a treadmill or semi-supine bike. Assessment of diastolic function at any point, whether at rest or during exercise, relies on separation of the early (E, e′ and E deceleration time) and late (A and a′) filling Doppler signals. As increasing HR decreases the diastolic filling period and causes early and late diastolic waveforms to merge, identification of the E max velocity becomes increasingly difficult at HR around 100–105 bpm.

When performed using the treadmill, the assessment of diastolic function is made during the recovery phase. With bike stress echo, assessment of diastolic function is not limited to recovery alone and can be performed during exercise. This not only offers the additional benefit of correlating symptom onset with the estimation of LVFP at that time, but also allows the exercise protocol to be tailored to the patient’s symptoms, exercise capacity and echo findings.

Once the patient is connected to all monitoring equipment (usually 12-lead ECG, BP and O_2_ saturation monitor) the bike is reclined and tilted leftward until images of diagnostic quality are obtainable. Once in the exercise position, the echo windows should be optimised. Parameters that are to be measured during exercise should be re-measured with the patient in the exercise position to establish baseline reference values in the new windows. For those who are physically able, a protocol starting with 25W resistance and increasing by 25W at 2-min intervals is appropriate. For those who have a low level of exercise capacity, a lower resistance protocol should be considered (starting resistance of 10W with 2-min increments of 10W). A standard cycle protocol directs a cycle rate of 55–65 rpm, although this can be tailored to control HR response and enable image acquisition at the required HR. As part of the ESE, assessment of significant CAD and valve disease may also be considered. The test for ischaemia typically aims to achieve 85% of target HR (maximum age predicted) in the absence of symptoms. Measures of diastolic function can therefore be performed both pre and post maximal exertion when heart rates are between 95 and 105 bpm.

#### Performing a diastolic ESE—measurement protocol

The measurement protocol for assessing diastolic function during exercise incorporates the Doppler parameters that are performed at rest (Table 8). During each stage, at peak exercise and during recovery, the following diastolic parameters are acquired:

- Mitral annular septal and lateral e′
- Transmitral E velocity
- TR velocity

**Table 8:** Table for the interpretation of diastolic parameters measured during stress echocardiography. Adapted from Nagueh et al. [85]

Interpretation of diastolic parameters during stress echocardiography		
	Normal	Abnormal
Average or septal only E/e′	< 10	> 14 (> 15 if septal only)
TR velocity	≤ 2.8 m/s	> 2.8 m/s

Where exclusion of CAD is the main indication, a maximal stress test is performed and 2D images prioritised for assessment of regional wall motion abnormalities (RWMA). If contrast is required for improved endocardial definition, tissue Doppler signals for diastolic assessment may be unobtainable; TR velocity (if measurable) maybe the only indicator of mean LVFP in this case. At peak exercise, assessment of RWMA and systolic function can be performed for 60 s post-peak stress. If both E/A and e′/a′ signals remain fused after this time, continue to assess TR velocity until HR allows diastolic assessment. It is important to bear in mind that pulmonary artery pressure is the product of flow (SV) and pulmonary artery (PA) vasculature resistance. SPAP may therefore become raised during exercise in those who are elderly with reduced PA compliance (resistance), or in athletes who have normal PA compliance but augment SV and CO massively (flow). Caution must therefore be exercised when interpreting TR velocity alone.

#### Interpretation of results

Interpreting the results of the diastolic stress echocardiogram should incorporate exercise induced symptoms, haemodynamic factors (HR and BP) and patient age. Echocardiographic parameters are suggestive of impaired diastolic function with exercise induced elevation of LVFP when E/e′ > 14 and TR velocity > 2.8 m/s [85]. However, irrespective of LV diastolic function, SPAP and therefore TR velocity may increase at higher HR and CO in the elderly. Importantly, the E/e′ ratio remains a single parameter within the algorithm for assessing LV diastolic function. As such, although a ‘positive’ test, where E/e′ ratio exceeds 14 during exertion, is strongly suggestive of exercise-induced elevation of LVFP, a ‘negative’ test with an E/e′ < 14 throughout exercise does not confirm normal LVFP [174, 176]. In this situation, the findings of the test should be considered within the clinical context and alongside patient symptoms.

#### Diastolic ESE Summary

ESE is a non-invasive, physiological, and convenient investigation to evaluate symptomatic patients with suspected diastolic heart failure. Diastolic stress testing is particularly suited for those with evidence of impaired relaxation yet normal LVFP at rest, and where pulmonary disease and other significant cardiac causes have been excluded. In comparison to the treadmill, bike stress testing offers the major advantage of real time assessment of diastolic haemodynamic parameters in conjunction with patient symptoms.

### Leg-raises and preload increase

LV diastolic pressures are determined by LV compliance and filling volume, manoeuvres to increase LV preload can therefore help unmask elevated LVFP [177]. Although this is more definitively achieved by exercise, passive leg-raise (PLR) increases LV preload through increasing venous return and can be considered as an ad-hoc addition to routine TTE in those with symptoms of exertional dyspnoea, impaired LV relaxation on TTE yet normal LVFP at rest and with no identifiable cardiac cause of symptoms. When positive, the increased LV preload into an incompliant LV causes elevated LVFP that are identifiable by the standard diastolic algorithm. However, a negative test does not rule-out more significant diastolic function impairment and ESE for diastolic assessment may be considered. Although PLR may increase LV preload and cause LVFP to become elevated, active leg raises, where each leg is alternatively raised and lowered, introduces an element of exercise and may help augment this response.

### Valsalva manoeuvre

Given the load dependency of diastolic pressures, performing manoeuvres that offload the left heart, such as the Valsalva manoeuvre, can have the opposite effect of the diastolic stress-test and can reveal an impaired relaxation filling pattern (ratio < 1) when LVFP is otherwise increased and the E/A ratio ‘pseudo-normalised’ to > 1 [178]. When the Valsalva manoeuvre is performed effectively, increased thoracic pressure decreases venous return and thus reduces left heart filling. With a reduction in left sided volume, LAP and LV diastolic pressures fall, leading to a decrease in E velocity. Reduction in LV pressure in late diastole decreases LA afterload, allowing LA contraction volume, and therefore velocity, to increase and thus causing the E/A ratio to reverse [179]. When restrictive filling physiology is present but Valsalva manoeuvre offloads the left heart, reducing E velocity and E/e′ and reversing the E/A ratio, LV filling is considered restrictive but reversible. However, when an effective Valsalva manoeuvre does not offload the left heart and LVFP and LAP remain high, filling is considered restrictive and irreversible, portending a poorer prognosis. Although the resting E/A ratio alone may not differentiate normal from raised LVFP, other standard measures of diastolic assessment should help diagnose impaired diastolic function and identify raised LVFP. Therefore, the Valsalva manoeuvre may only be required to distinguish fixed from reversible restrictive filling.

### Performing the Valsalva manoeuvre

Measurements should be taken at inspiration and throughout 10 s of forced expiration against a closed glottis. A slow sweep-speed can capture the full manoeuvre. It is difficult to perform well—the expiratory pressure must be maintained for 10 s and the sample volume kept at the same position throughout. A decrease in the MV E-wave of 20 cm/s suggests a good technique. If the E/A ratio decreases by ≥ 50% or the A-wave increases (but not due to E & A fusion) then this is highly specific for raised LVFP. A normal response is a balanced reduction in E and A velocities and an increased heart rate.

## Future directions of the diastolic function assessment

### LV untwisting and diastolic strain analysis

Since myocardial deformation is altered despite normal LVEF in diseases that predispose to impaired diastolic function and HFpEF (obesity, DM, renal disease, HTN and age) [180], diastolic strain and parameters of LV untwisting may be useful indicators of LV diastolic function. Whereas GLS measures longitudinal deformation of the myocardium in systole and is reflective of global systolic function, untwist and torsional mechanics are important components of LV diastolic recoil and LV filling and can be described according to: the degrees of basal or apical rotation, rotation relative to ventricular length and the rate at which this occurs. Therefore, assessment of untwisting and torsion mechanics may help identify impairment of LV filling [181].

Given that over 40% of LV untwisting is achieved within the first 15% of the diastolic period [182], untwist during the IVRT appears to be important for global diastolic function. In one hybrid animal-human study, diastolic strain rate (DSR) was able to identify impaired LV relaxation with a significant inverse correlation existing between SR during the IVRT (DSR_IVR_) and τ in animals (r = 0.83) and in humans (r = 0.74); a positive yet less strong correlation was found in animals between DSR_IVR_ and -dP/dt (r = 0.71) [183]. When considered alongside Doppler parameters of LV filling, diastolic strain analysis was also able to predict LVFP. The ratio of transmitral E to DSR_IVR_ and SR in early diastole (DSR_E_) were found to correlate directly with PCWP, with E/DSR_IVR_ demonstrating the strongest relationship (r = 0.79) [183, 184]. Although E/DSR_IVR_ could not predict a specific PCWP, all patients, except one, with a ratio of < 236 had a PCWP of < 15 mmHg, whereas all patients, except one, with a ratio > 300 had a PCWP of > 15 mmHg; 75% of the patients in this study with a ratio of 236–300 had a PCWP of > 15 mmHg. In addition, E/DSR_IVR_ was found to be more accurate than E/e′ at predicting raised LVFP in patients with normal LVEF or regional impairment. The correlation of PCWP with E/DSR_E_ was significant but weak in comparison (r = 0.46). Although DSR_IVR_ is preload dependent (increasing when LVFP increases), the load dependency only becomes significant when LV relaxation is normal, similar to E/e′. When LV relaxation is impaired the influence of preload is less significant and DSR_IVR_ is reduced in the setting of impaired diastolic function [183]. In another animal study, the authors measured the peak untwisting velocity in healthy pigs in comparison to pigs with induced metabolic syndrome. At three months, the peak untwisting velocity was unchanged in the healthy pigs yet significantly reduced in those with induced metabolic dysfunction, despite no significant change in E/A ratio or E/e′ [185], suggesting a role for untwisting velocities in the assessment of subclinical diastolic impairment.

However, in canine studies, LV untwisting rate (UR) was found to be heavily influenced by loading conditions, LV end-systolic volume (ESV) and systolic contractility, such that decreasing LV ESV or increasing LV twist through Dobutamine infusion resulted in a greater rate of LV untwisting in early diastole but without a significant change in τ [186]. Even when τ was significantly lengthened by beta-blocker infusion, the LV UR remained unchanged when ESV was reduced, suggesting that UR is significantly dependent on LV ESV. In HFpEF and HFrEF patients, the peak UR was related to indicators of LV contractility (LV twist and ESV) and was only related to τ in those with reduced LVEF. In the same study, irrespective of the presence of HF, LV twist, a seemingly important factor influencing untwist, was found to be decreased at rest in those with reduced LVEF yet normal in those with impaired diastolic function but normal LVEF, further supporting the notion that systolic twist is a key determinant of diastolic UR. Importantly, LV UR is reduced during exercise in patients with HFpEF.

As with other parameters of LV filling, age influences the rate of diastolic recoil and therefore strain parameters measured during this period. As previously described, LV recoil during the IVRT and early filling period is achieved, in part, through release of potential energy stored within the elastic elements of myocytes during systolic deformation and twist. However, degenerative changes through normal aging reduce the elastic resistance of the myocardium and attenuates the degree of potential energy stored within the twisted myocardium, thereby reducing the relative peak diastolic untwisting velocity and untwisting rate and delaying the time to peak untwisting velocity [9, 187, 188].

The assessment of diastolic strain appears promising for future iterations of diastolic guidelines but remains a research tool at this time and is not currently recommended for the routine application of LV diastolic assessment.

### Multivariate reference regions

**Fig. 21:**
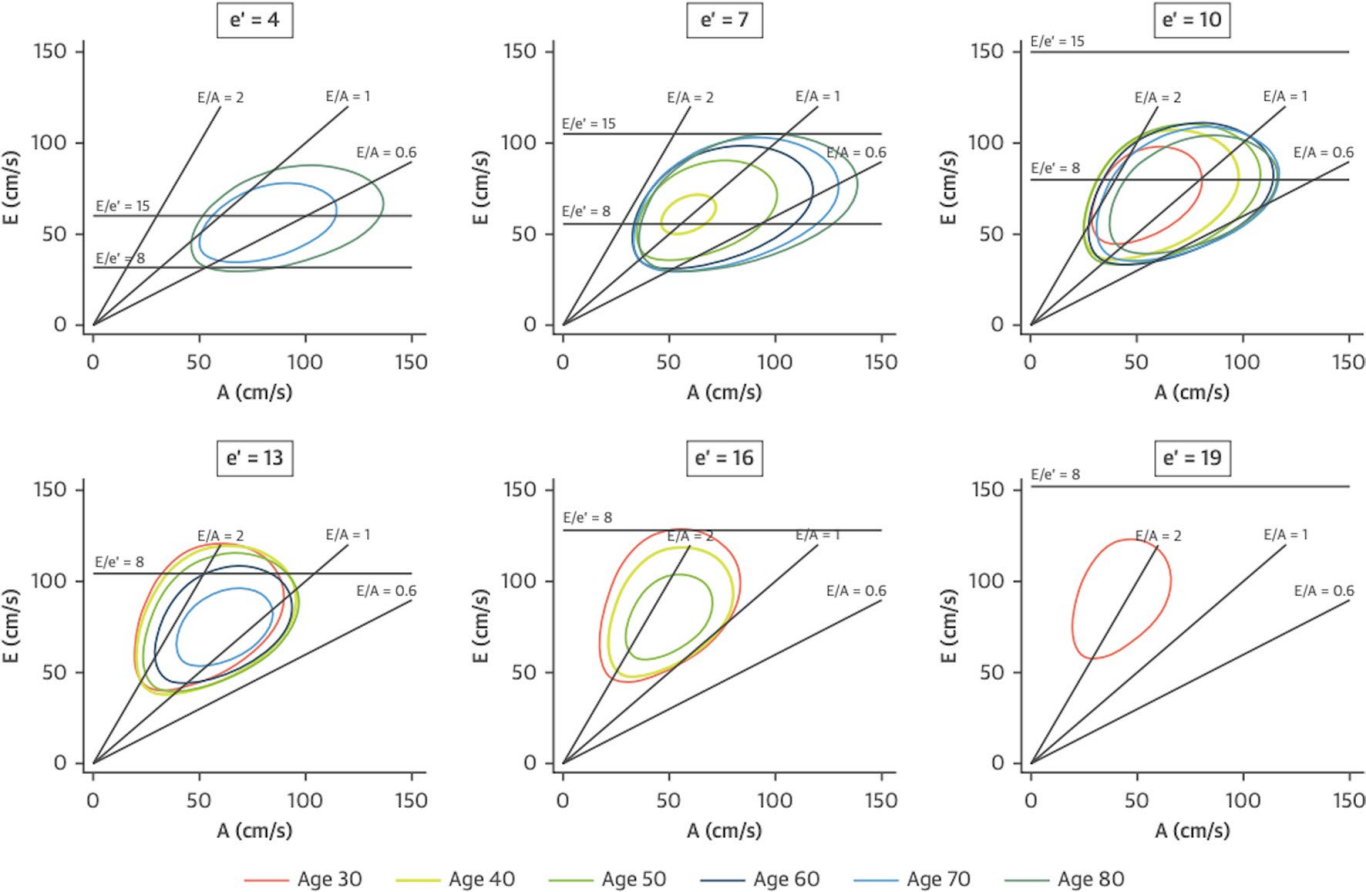
Age-specific datasets plotted for E, A and e′ result in a three-dimensional skewed ellipsoid reference region. Here, the colour-coded ellipsoids have been sliced and displayed to demonstrate the expected E and A velocity and therefore E/A ratio according to six incremental e′ velocities. For example, it would be expected that for an individual in their thirties with an average e′ of 16 (centre-bottom graph), the E and A velocity would fall within the red ellipsoid and the E/A ratio would not be expected to fall below 1. Furthermore, the E/ e′ would not be expected to exceed 8. From Selmeryd et al. [189]

Assessment of diastolic parameters according to multivariate reference regions may be considered more broadly in future diastolic function guidelines. When large-scale studies have sought to identify normal echocardiographic reference intervals for given parameters, the data for each measure is typically presented as the mean value accompanied by the standard deviation (SD) [91, 189]. As 2SD either side of the mean value provides us with a measurement range within which 95% of all measures lie, we are provided with lower and upper limits of normal (LLN/ULN); measures that fall outside of this reference interval are considered highly likely to be abnormal. However, although this method provides us with an expected normal reference interval for individual univariate parameters, considering multiple univariate parameters side-by-side and simply dichotomising each one as normal or abnormal, as is required for the assessment of LVDF, is problematic. For example, three separate variables at the very lower end of their normal reference interval (ie, e′ velocity, E velocity and E/A ratio) may be individually normal as they are all (just) within a normal reference interval, but the combination of three very low-normal values may be abnormal and indicative of disease. When considering age-specific multivariate reference regions for identifying abnormal filling patterns, using multiple univariate reference intervals side-by-side to confirm abnormality in this way is not advisable [189]. By doing so: (a) increases the risk of false-positives, (b) reduces test sensitivity and (c), because discrepancies are common among groups of measurements with univariate reference intervals, large proportions of patients may be deemed unclassifiable. When reviewing multiple univariate parameters of diastolic function, Selmeryd et al. demonstrate multivariate analysis for age-specific patterns of LV inflow (E/A) that incorporates velocity of E, A and e′, identifying expected E and A velocity, E/A ratio and E/e′ according to age and e′ velocity [189] (Fig. 21). By doing so, a more global consideration of these parameters is possible and within the context of other univariate parameters.

The authors go on to report that in those with normal healthy hearts without impaired diastolic function, the multivariate upper limit of E/e′ in the young is around 8, whereas a ratio of 15 may be considered normal in older subjects with normal hearts if the E/A ratio is < 1 and/or e′ is < 7 cm/s, highlighting the contextual role of E/e′ in the assessment of diastolic function. However, current guidance continues to apply a single and high E/e′ cut-off value for all ages. Although this provides high specificity for elevated LVFP, it renders the ratio insensitive to the detection of impairment leading up to this point. While a cut-off of > 14 is optimal for identifying PCWP > 15 mmHg, there is clearly a period prior to the development of elevated LVFP when E/e′ is increasing and potentially indicative of impaired diastolic function in the young, but not yet at the required cut-off for suggesting raised LVFP and therefore diastolic impairment. Conversely, and as described, E and e′ are continuous variables with significant dependency on age. However, because of the disproportionate age-related decline between the two variables (e′ decreasing to a greater extent than E), the E/e′ ratio increases with normal aging (8.2 ± 2.2 at age < 45 years and 12.4 ± 3.3 at ≥ 75 years) [91]. Despite this progressive increase with age, a single cut-off for diagnosing impaired diastolic function applies to all age groups. This naturally impairs the ability of E/e′ to detect impaired diastolic function in the young (where an E/e′ > 9 is extremely uncommon [91, 189, 190]) and risks over-diagnosis and false positives in the elderly. Applying such multivariate reference regions may improve the diagnosis of impaired diastolic function in the future.

### LA stiffness index

The LA stiffness index is a measure that incorporates echocardiographic estimates of LAP (E/e′) and LA function (LARs) to describes the compliance properties of the LA in the setting of impaired diastolic function [191] – (E/e′) / LARs. In those with diastolic impairment and raised LVFP, elevated LAP causes increased LA wall stress, decreasing LA compliance and leading to reduced LA filling [87, 192].

Greater LA stiffness has been identified in HFpEF patients [83, 101, 193] with reportedly greater accuracy for the diagnosis of HFpEF than LARs alone [193, 194], although this is likely due to LA stiffness indicating a well-established and more advanced stage of impaired diastolic function than raised LVFP alone, therefore explaining the closer correlation with adverse outcomes [195]. Furthermore, although LVFP may be reduced through optimal medical management, the chronic morphological and functional alterations of the LA persist and identifies an increased risk of adverse outcomes that would be missed by assessment of LVFP alone [195]. Additionally, it is very common in patients with impaired diastolic function for LVFP to be normal at rest but for LA function and stiffness to be abnormal and therefore detectable by assessment of LA stiffness [195]. Prognostically, increased LA stiffness is recognised in patients with chronic AF due to the development of LA myocardial fibrosis and is associated with higher rates of AF recurrence following AF ablation [196].

Although reference value cut-offs have been published that identify an increased risk of heart failure hospitalisation and mortality in patients with increased LA stiffness (an index of > 0.26 identified patients who were at greater risk of heart failure hospitalisation or death at five years in those with HFpEF (LVEF ≥ 50%) and LVEDP > 16 mmHg [190]), these parameters have not been validated. Furthermore, no data is currently available for LA stiffness index in those with normal and compliant LA [195]. Although the published data suggests that the LA stiffness index provides important diagnostic and prognostic value, this important physiological and haemodynamic concept may be considered but is not recommended for routine clinical practice.

### LV diastolic function reporting recommendations

Previous guidelines for the assessment of LVDF have classified the degree of impairment into grades I, II and III or mild, moderate and severe, where: grade I/mild impairment identifies impaired ventricular relaxation but with no evidence of raised LVFP; grade II/moderate indicates a more advanced degree of diastolic impairment with raised LVFP; grade III/severe identifies restrictive filling with significantly raised LVFP and is further defined as reversible or irreversible according to the response of LVFP to Valsalva manoeuvre. In this guideline, the BSE have departed from reporting diastolic function according to numerical grades (I, II, III) or grades of inferred severity (mild, moderate or severe). These grading systems are largely unfamiliar to clinicians outside of cardiology and infer a degree of significance that may be contradictory to the clinical context—impaired LV relaxation with normal LVFP in a young athlete is of markedly different clinical significance than for a 75 year-old hypertensive patient, yet would be described as mildly impaired for both. Furthermore, the role of echocardiography is to describe diastolic function and its effect on LVFP. Although there are instances where medical management may reverse the process that has caused impaired diastolic function (for example, enzyme replacement therapy for Anderson-Fabry’s disease, LV mass regression following aortic valve replacement), in many cases the medical optimisation of patients with more significant diastolic function impairment includes off-loading of the left heart in order to reduce LVFP, leading to symptomatic improvement. As such, reduction in LVFP through medical management may give the false impression of improving myocardial function on serial echocardiography when reported by grades or perceived severity (‘improving’ from moderate to mild, grades II to I), when in fact it is merely a reduction in LVFP. Rather, the BSE recommend that the spectrum of diastolic function should be reported according to the continuum of physiology that it is, ranging from a prolonged rate of ventricular relaxation and with no significant increase in LVFP in the initial stages, through to decreased myocardial compliance and consequently elevated LAP. The BSE therefore recommend that the reporting of impaired diastolic function is in accordance with the observed physiology and haemodynamic sequelae; the recommended reporting statements are as follows:

- Normal diastolic function for age
- Impaired LV diastolic function with normal filling pressure at rest
- Impaired LV diastolic function with elevated filling pressure at rest

In those with elevated LVFP, LVEF ≥ 50%, no more than moderate left-sided valve disease and symptoms of exertional breathlessness with unknown cause, the following statement may be considered for inclusion within the report:

- these findings may be consistent with HFpEF and should be considered in the context of clinical presentation and symptoms.

## Conclusion

The assessment of LVDF is complex, requiring a multiparametric approach to the investigation of relaxation, chamber compliance and filling pressures. However, by considering the recommendations within this guideline alongside the patient’s clinical presentation, and therefore identifying the pre-test probability of impaired diastolic function, a successful investigation of diastolic function and LVFP is possible is most patients.

### Supplementary Information

Additional file 1.

## Data Availability

All data generated or analysed during this study are included in this published article [and its supplementary information files].

## Abbreviations

AF: Atrial fibrillation
AR: Aortic regurgitation
AV: Aortic valve
BP: Blood pressure
BSA: Body surface area
CAD: Coronary artery disease
CFD: Colour Flow Doppler
CMM: Colour M-mode
CO: Cardiac output
CW: Continuous wave
DSR: Diastolic strain rate
DSRIVR: Diastolic strain rate during isovolumetric relaxation time
DSRE: Early diastolic strain in sinus rhythm
ESE: Exercise stress echocardiography
ESV: End systolic volume
GLS: Global longitudinal strain
HF: Heart failure
HCM: Hypertrophic cardiomyopathy
HFpEF: Heart failure with preserved ejection fraction
HFrEF: Heart failure with reduced ejection fraction
HR: Heart rate
IVCT: Isovolumetric contraction time
IVRT: Isovolumetric relaxation time
LA: Left atrium
LAP: Left atrial pressure
LACs: Left atrial conduit strain
LAPs: Left atrial pump strain
LARs: Left atrial reservoir strain
LAs: Left atrial strain
LAVi: Left atrial volume index
LBBB: Left bundle branch block
LH: Left hand
LV: Left ventricle
LVFP: Left ventricular filling pressure
LVDF: Left ventricular diastolic function
LVEDP: Left ventricular end diastolic pressure
LVEF: Left ventricular ejection fraction
LVOT: Left ventricular outflow tract
MAC: Mitral annular calcification
MI: Myocardial infarction
MoD: Method of disks
MR: Mitral regurgitation
MS: Mitral stenosis
MV: Mitral valve
MVR: Multivariate reference regions
PA: Pulmonary artery
PCWP: Pulmonary capillary wedge pressure
PH: Pulmonary hypertension
PV: Pulmonary vein
PW: Pulse wave Doppler
RA: Right atrium
RAP: Right atrial pressure
RH: Right hand
RV: Right ventricular / right ventricle
RWMA: Regional wall motion abnormalities
SBP: Systolic blood pressure
SPAP: Systolic pulmonary artery pressure
SR: Sinus rhythm
SV: Stroke volume
TDI: Tissue Doppler imaging
TR: Tricuspid regurgitation
TTE: Transthoracic echocardiography
UR: Untwisting rate
Vp: Propagation velocity

## Key terms

**Compliance**: Compliance describes the change in intracavity pressure secondary to an increase in cavity volume such that a highly compliant chamber is able to increase volume significantly (distensibility) with only a small associated increase in internal pressure (compliance = ΔV/ΔP where V = volume and P = pressure). Within an incompliant (stiff) chamber, pressure rises rapidly with only small increases in volume
**Distensibility**: Describes the ability of a chamber to stretch/expand to increase volume. A highly compliant chamber is therefore distensible whereas an incompliant and stiff chamber is unable to stretch/expand and is therefore of low distensibility
**Mean left atrial pressure (LAP)**: Left atrial pressure varies throughout the cardiac cycle. The V-wave occurs during ventricular systole peaking at 6–12 mmHg, while the A-wave occurs during atrial contraction peaking at 4–16 mmHg. Mean LAP is between 2–12 mmHg
**Left ventricular end-diastolic pressure (LV EDP)**: LV end-diastolic pressure occurs following atrial contraction and immediately prior to the rapid increase in pressure at the onset of systole. LVEDP is normally between 4–12 mmHg
**Pulmonary capillary wedge pressure (PCWP)**: Due to the continuity between the LA, pulmonary veins and pulmonary vasculature, left atrial pressure can be measured by ‘wedging’ a balloon-tipped catheter within a distal pulmonary artery
**Left ventricular filling pressures (LVFP)**: Due to the wide range of pressure throughout diastole, LV filling pressures is used as a general term within this guideline to describe the pressures under which LV diastolic filling occurs—ie, normal versus elevated LV filling pressure
**Preload**: Describes the degree of myocyte stretch prior to LV contraction. As this cannot be measured directly by echocardiography, LV end-diastolic volume is considered a surrogate of LV preload. LV preload is increased by severe aortic or severe mitral regurgitation
**Afterload**: Describes the resistance against which the LV fibres contract against in systole. Elevated systolic blood-pressure and aortic stenosis increase LV afterload
