# Supplementary material for: The assessment of left ventricular diastolic function: guidance and recommendations from the British Society of Echocardiography
Source: Echo Res Pract. 2024 Jun 3;11:16. doi: 10.1186/s44156-024-00051-2 (PMC11145885; doi:10.1186/s44156-024-00051-2)
Supplement: Supplementary file 1 — Additional file 1. [file 44156_2024_51_MOESM1_ESM.docx]

**The Assessment of Left Ventricular Diastolic Function: a quick-reference guide from the British Society of Echocardiography**

Shaun Robinson^1^ (lead author), Liam Ring^2^, David Oxborough^3^, Allan Harkness^4^, Sadie Bennett^5^, Bushra Rana^1^, Nilesh Sutaria^1^, Francesco Lo Giudice^1^, Matthew Shun-Shin^1^, Maria Paton^6^, Rae Duncan^7^, James Willis^8^, Claire Colebourn^9^, Gemma Bassindale^6^, Kate Gatenby^6^, Mark Belham^10^, Graham Cole^1^, Daniel Augustine^8^ and Otto A. Smiseth^11^

1 – Imperial College Healthcare NHS Trust, London, UK

2 – West Suffolk Hospital NHS Trust, Bury St Edmunds, UK

3 – Liverpool John Moore’s University, Liverpool, UK

4 – East Suffolk and North Essex NHS Foundation Trust, Colchester, UK

5 – University Hospital of the North Midlands, Stoke-on-Trent, UK

6 – Leeds Teaching Hospitals, Leeds, UK

7 – Newcastle upon Tyne Hospitals NHS Foundation Trust, Newcastle, UK

8 – Royal United Hospital Bath, Bath, UK

9 – Oxford University Hospitals, Oxford, UK

10 – Addenbrookes Hospital, Cambridge University Hospitals, Cambridge, UK

11 – Institute for Surgical Research, Oslo University Hospital and University of Oslo, Oslo, Norway

Corresponding author: Shaun Robinson – shaunrobinson@nhs.net

**Introduction**

The echocardiographic assessment of diastolic function is complex. No one echocardiographic parameter can unequivocally confirm impaired diastolic function, rather a combination of several measures is required. Nevertheless, because diastolic function impairment is an increasingly important cause of cardiovascular morbidity and mortality, being the primary functional abnormality in those with heart failure with preserved ejection fraction (HFpEF), the analysis of diastolic parameters is essential during standard echocardiography.

A comprehensive description of normal diastolic physiology and pathophysiology, natural history, and the range of echocardiographic tools available for investigating diastolic function are detailed within the main text of this diastolic guideline. The full guideline also provides context to the current and future state of diastolic function assessment, along with a detailed rationale as to the decisions made regarding the current BSE guidance. This pocket guideline is therefore a focussed summary of the key measures and principles that should enable echocardiographers to undertake diastolic assessment accurately in day-to-day practice. The strengths and weaknesses of each diastolic measure are covered in detail within the main text. In order to ensure the diagnostic accuracy of each algorithm, it is recommended that all echocardiographers applying this BSE guidance are familiar with the scenarios in which the strength of each parameter might be compromised.

**Categories of diastolic function**

Reporting of diastolic function according to three categories is recommended, these are:

- Normal diastolic function for age.
- Impaired diastolic function with normal filling pressures.
- Impaired diastolic function with elevated filling pressures.

In some scenarios a comprehensive assessment of diastolic function will not be feasible, in which case the report should comment that the assessment of filling pressures is ‘indeterminate’. In those with elevated filling pressures, LVEF >50%, no more than moderate left-heart valve disease and symptoms of breathlessness with no known cause, the following statement may be considered for inclusion within the report – *these findings may be consistent with HFpEF and should be considered in the context of clinical presentation and symptoms*.

Table 1 describes practical guidance for image acquisition and measurement of the **routine parameters** for the assessment of LV diastolic function. In the majority of cases, these simple parameters (which are included in the BSE minimum dataset) will be sufficient to classify diastolic function.

**Table 1**

**Approach**

The echocardiographic approach to investigating LV diastolic function and filling pressures is simplified and algorithm accuracy is improved if the assessment is considered according to the clinical scenario. Therefore, rather than a single algorithm for use in all patients, with consequently limited sensitivity/specificity, this guidance incorporates three algorithms that are specific to the clinical scenarios of: a) abnormal LV systolic function or known myocardial disease, b) normal LV systolic function and c) atrial fibrillation.

***Normal systolic function***

In patients with normal systolic function, any of the three categories of diastolic function are possible: normal diastolic function for age; impaired diastolic function with normal filling pressures; impaired diastolic function with elevated filling pressure. Therefore, the algorithm is structured to enable the echocardiographer to identify the presence or absence of elevated filling pressures. If filling pressures are deemed normal, the assessment of relaxation can then be made according to age-specific e′. The approach to assessing diastolic function in patients with normal systolic function is outlined in Figure 1.

**Normal systolic function**

**Avg E/e′ >14 - LA volume >34 mL/m**² **- TR velocity >2.8 m/s**

**Normal LV systolic function**

2 or 3 criteria negative

Only 2 criteria available: 1 positive and 1 negative

2 or 3 criteria positive

Assess LA strain:

pump strain ≥14% **OR** reservoir strain ≥30%

No

Yes

Yes

LARs <18%

No

**Assess supplementary parameters:**

Ar-A duration >30 ms

L-wave >20 cm/s

Assess relaxation by
age-specific e′

No

≥1 positive

Normal e′

e′ <LLN

If clinically indicated, exercise echo may be considered to investigate elevated filling pressures on exertion

Impaired diastolic function with normal filling pressures

Impaired diastolic function with elevated filling pressures

Normal diastolic function

Fig 1 – algorithm for the assessment of LVDF in those with normal systolic function

**Impaired systolic function or known myocardial disease**

In patients with impaired systolic function, a degree of impaired diastolic function is anticipated. Therefore, in this setting, the focus of the diastolic assessment is simply to establish whether filling pressures are elevated or not. Furthermore, because the pre-test probability of elevated filling pressures is greater than in those with normal systolic function, more sensitive thresholds for certain supplementary parameters can be considered. The approach to assessing diastolic function in patients with abnormal systolic function is outlined in Figure 2.

**Impaired systolic function** **or known myocardial disease**

**LVEF <50% OR**

**GLS <16% OR known myocardial disease**

Diastolic function is impaired, assess filling pressure

**Avg E/e′ >14 - LA volume >34 mL/m**² **- TR velocity >2.8 m/s**

2 or 3 criteria negative

2 or 3 criteria positive

Only 2 criteria available: 1 positive and 1 negative

Assess LA strain

LARs <18% **OR** LAPs <8%

No

LARs ≥24% **OR** LAPs ≥14%

Yes

No

Yes

**Assess supplementary parameters:**

Ar-A duration >30 ms

L-wave >20 cm/s

PV S/D ratio <1

DT_E_ <150 ms

≥1 positive

No

Impaired diastolic
function with normal filling pressures

Impaired diastolic function with elevated filling pressures

2 or 3 criteria positive

Fig 2 - algorithm for the assessment of LVDF in those with impaired systolic function or known myocardial disease

**Atrial Fibrillation**

In the setting of AF, the assessment of relaxation is complicated by variable cardiac cycle length, and consequently loading conditions, that cause significant beat-to-beat variation of diastolic parameters. The focus of the diastolic assessment is primarily to establish whether filling pressures are elevated or not. In AF, the BSE recommends that the ‘index beat method’ is used for obtaining Doppler parameters - heart rate should be <100bpm and ideally <90bpm. Doppler parameters can then be obtained from a single beat if **the two preceding R-R intervals are of similar duration (within 60ms of one another).** When deriving the E/e′ ratio, it is important that the E velocity and the e′ velocity are measured from cardiac cycles that are similar length. In those with both AF and LV systolic function impairment or myocardial disease and in whom the assessment of supplementary parameters is required, it is recommended that the supplementary measures from the abnormal LV function algorithm are utilised, where possible.

**Atrial fibrillation**

**Atrial fibrillation**

Heart rate <120bpm; ideally use index beat method

**STEP 1**

**Septal E/e′ >11 Mitral E velocity ≥100 cm/sec E decel time ≤160 ms TR velocity >2.8 m/s**

Insufficient STEP 1 criteria for decision

≥3 criteria negative

≥3 criteria positive

**STEP 2**

**LA reservoir strain <16% BMI >30kg/m^2^ Pulmonary venous S/D ratio <1**

≥2 STEP 2 criteria negative

Insufficient STEP 2 criteria

≥2 STEP 2 criteria positive

**Indeterminate filling pressures**

**Normal filling pressures**

**Impaired diastolic function with elevated filling pressures**

Fig 3 - algorithm for the assessment of LVDF in those in atrial fibrillation

**Table 2**

| **Septal and lateral e′ values suggestive of impaired LV relaxation** | | | | |
| --- | --- | --- | --- | --- |
| **Age** | **18 – 40 y** | | **41 – 65 y** | **>65 y** |
| **Sex** | **Male** | **Female** | **All** | **All** |
| **Septal e′ (cm/s)** | **<7.0** | **<8.0** | **<5.0** | **<4.0** |
| **Lateral e′ (cm/s)** | **<9.0** | **<11.0** | **<6.0** | **<5.0** |

Table 2. Septal and lateral e′ values suggestive of LV diastolic function impairment. Any value below that stated within the table is suggestive of impaired LV relaxation.

**Occasional clinical scenarios**

The assessment of LV diastolic function is complicated by disease processes or clinical scenarios that affect the routine parameters of the LV diastolic assessment. Comprehensive guidance for how to assess diastolic function in these patient groups can be found in the main guideline document. Table 3 outlines the supplementary and non-routine measures that can be considered in each setting.

**Table 3**

| **Specific measures and considerations in special groups** | |
| --- | --- |
| **Clinical setting** | **Markers of raised LAP** |
| Mitral stenosis (>mild) | IVRT/T_E-e′_ < 4.2 |
| Mitral regurgitation (>moderate) | IVRT/T_E-e′_ < 5.6 if normal LVEF (more specific if < 3). |
| Mitral annular calcification | TR velocity >2.8 m/s, normal LAP (<0.8) from raised LAP (>1.8); for those with an intermediate ratio (0.8 – 1.8), an IVRT of <80 ms identifies raised LAP (146). |
| MV repair/replacement | IVRT and IVRT/TE-e′ may be of use |
| Aortic regurgitation | Routine measures can be applied |
| Recent DC cardioversion from AF | Septal E/e′ ratio ≥ 11, mitral E deceleration time ≤ 150 ms in the setting of LV systolic dysfunction, IVRT ≤ 65 ms, DT of pulmonary vein diastolic velocity ≤ 220 ms, Mitral L-wave > 20 cm/s, TR velocity > 2.8 m/s, acceleration of E ≥1900 cm/s^2^, E/Vp ≥1.4. |
| Sinus Tachycardia | Average E/e′ (>14 provides highest specificity, >10 is more sensitive but less specific), pulmonary vein systolic filling fraction ≤ 40% (if good tracings possible), IVRT ≤ 70 ms, TR velocity > 2.8 m/s. E/A >1 in patient with LVEF <50% (if pre-A velocity <20 cm/s). |
| AV Block, LBBB and paced rhythms | E/A fusion increases atrial pump SV and may result in a longer A wave duration, altering the A-Ar duration, and higher pulmonary venous systolic velocity leading to altered PV S/D ratio. If the fusion is minimal, grading of diastolic function may still be possible according to the standard algorithm. Decreased IVRT may help identify raised LAP. |
| Hypertrophic cardiomyopathy | Routine measures. Individual parameters, including E-e′, have weak correlation with filling pressure when the LVEF is normal |
| Post heart transplant | Raised SPAP in the absence of pulmonary disease. |
| Pre-capillary PH | Please see main guideline – Fig 20, page 90 |

Table 3 – specific measures for the assessment of LV diastolic function in certain patient groups

**Accuracy**

Every attempt has been made to ensure that the algorithms are as accurate as possible. However, it is important to appreciate that the echocardiographic assessment of diastolic function is imperfect and, in some patients, may provide inaccurate conclusions. It is therefore essential that clinical context is considered. For example, where a strong clinical suspicion of HFpEF exists, an echocardiographic report documenting completely normal diastolic function may be questioned and discussed. The BSE recommends that difficult or challenging cases are at least reviewed or brought to an echo MDT meeting to help improve reporting standards within each clinical setting.

**Algorithm limitations**

Discussed in more detail in the main guideline text, an algorithm that considers multiple univariate parameters side-by-side is inherently limited. However, identifying subtle and early indicators of myocardial impairment is key for ensuring optimum outcomes in those with confirmed disease and may be crucial for the early identification of cardiomyopathy. It is therefore recommended that the findings of the diastolic assessment are considered within the clinical context (conditions that increase the pre-test probability of diastolic impairment) and that considering the supplementary and non-routine measures will likely improve the overall assessment of LV diastolic function.

| **Routine measures of LV diastolic function** | |
| --- | --- |
| **Transmitral Doppler signals** | |
| In the apical 4-chamber view, place the PW Doppler sample volume (1–3 mm) at the level of the mitral leaflets. Colour flow Doppler must be used to align the sample with the centre of transmitral flow. This is especially important in the setting of LV dilation when transmitral inflow may be directed postero-laterally due to tethering of the mitral leaflets. Spectral gain, wall filters (100–200 MHz), baseline/scale and signal reject should be optimised to ensure a clear signal that identifies the onset and cession of transmitral flow. | |
| **Peak E and A velocity** | |
| **E wave** **velocity and deceleration time**  E **wave** **velocity:** peak modal velocity at the leading edge of the transmitral flow in early diastole (following the T-wave). **E deceleration time:** Time from the peak E-wave velocity to the point at which the E wave signal ends, measured along the deceleration slope of E wave signal - either when LA-LV pressures equalise and flow ends at the zero-velocity baseline or at atrial contraction and the onset of the A wave. When the E deceleration slope is bi-modal, the second and typically longer deceleration slope should be measured. For AF or variable R-R, ensure the two preceding R-R intervals are similar and that the heart rate <100 bpm. | 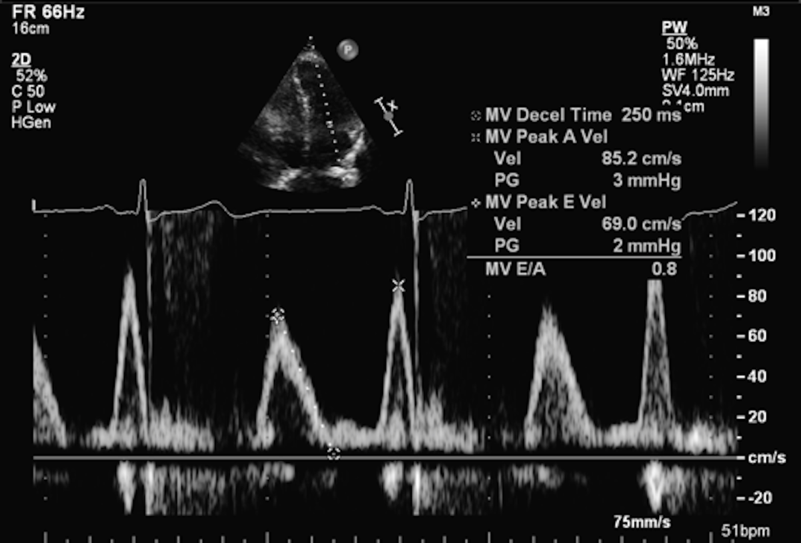 |
| **A wave** - peak modal velocity at the leading edge of the transmitral flow in late diastole (after the P-wave). Should not be measured in the setting of atrial arrhythmia. |  |
| **E/A ratio** – peak of the modal E velocity is divided by the peak of the modal A velocity. |  |
| **Pre-A velocity** - is only relevant and should therefore only be considered when the degree of E and A wave fusion causes the pre-A velocity to exceed 20 cm/s. Spectral Doppler gain and reject should be optimised to reduce transit time artefact. The velocity is measured at the point where the E and A waves meet. | 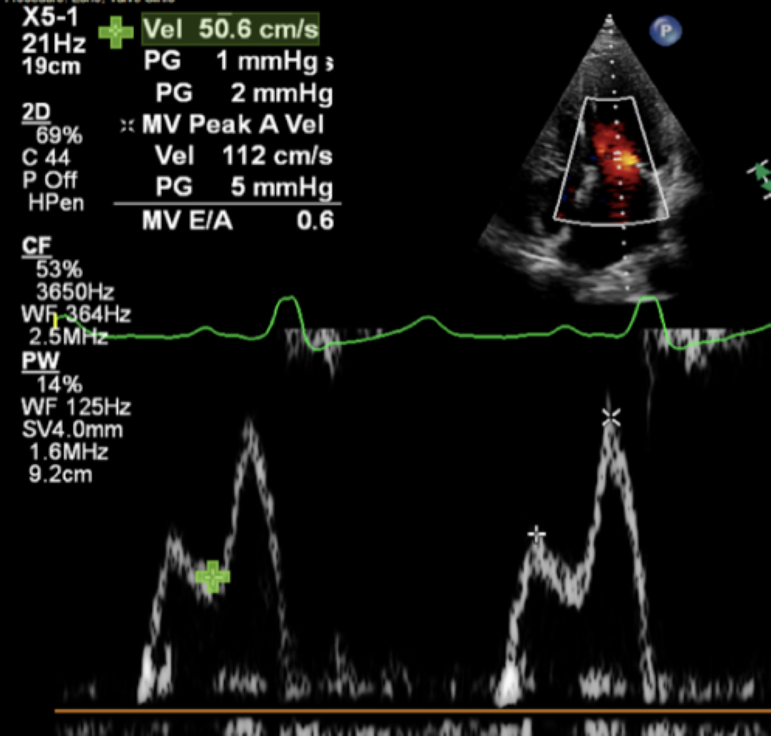 |
| **Mitral annular Tissue Doppler Imaging** | |
| The apical 4-chamber axis should be optimised for the annular region being sampled, ensuring the apically directed movement of the annulus is parallel to the cursor – this may require image adjustment when moving from the septal to the lateral wall. Place the PW tissue Doppler sample volume (5–10 mm) at or within 1 cm of the insertion of the mitral valve leaflets. Both the septal and lateral walls should be sampled and averaged where possible. | |
| **e′ -** peak modal velocity at the leading edge of the spectral waveform in early diastole (after the T-wave). Gain and reject settings should be optimised to display high amplitude annular velocities with clearly defined modal waveform. Measurements should be averaged over 3 cardiac cycles, at end expiration. | 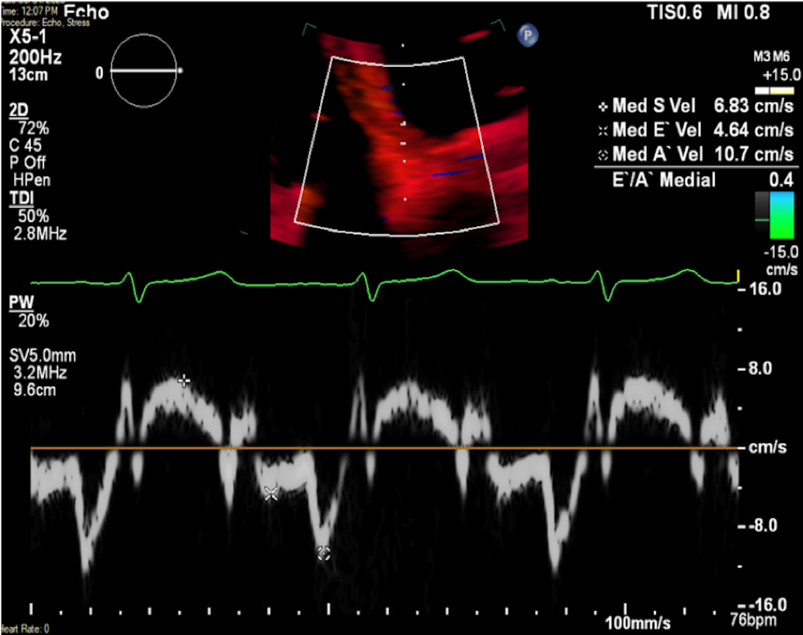  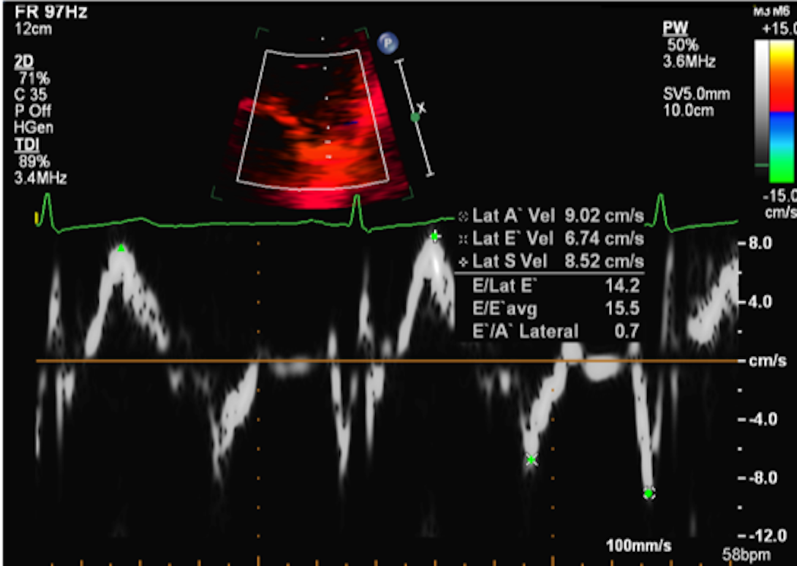 |
| **Average E/e′ -** MV E velocity divided by the average of septal and lateral wall e′ measurements. When only the septum or lateral wall are hypokinetic with no further regional wall motion abnormalities present, annular velocities at the base of the impaired wall are unlikely to reflect global myocardial function and should be avoided. Both measures should be made at end expiration and during similar R-R interval to ensure accurate comparison of values*.* |  |
| **LAVi** | |
| LA volume should be measured at left ventricular end systole (largest LA size at the frame just before mitral valve opening) using the Simpson’s biplane MoD and then indexed to BSA. Since apical views that are optimised for the LV will foreshorten the LA, dedicated apical 4-chamber and 2-chamber images should be acquired to maximise LA dimensions. The difference between the length of the 4-chamber (A4C) and 2-chamber (A2C) views should not exceed 5mm (105). Although simultaneous biplane acquisition utilising 3D imaging may improve measurement accuracy, the A4C and A2C views are not simply orthogonal imaging planes and each view must be optimised to maximise LA dimensions. Compression of the LA from the aorta may invalidate the measurement. Do not include the mitral valve tenting area below the annulus, appendage or pulmonary veins in the trace. The Area-Length method produces values that are 10–15% higher than the Simpson’s method (106). The BSE therefore recommend the Simpson’s method of LA volume estimation (80). | **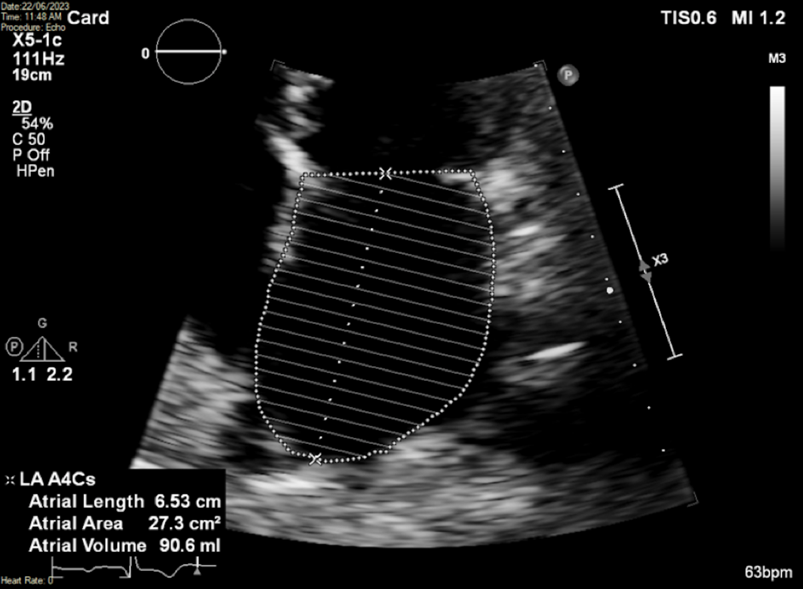**  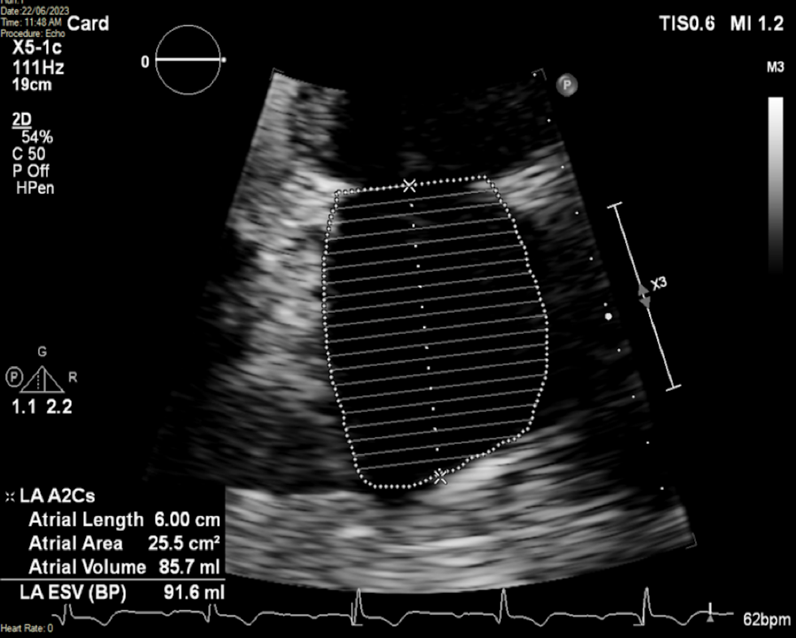 |
| **TR velocity** | |
| Place the CW Doppler sample through the TR flow, using colour flow Doppler to guide. Ideally, the CW cursor should be placed through the TR flow convergence zone (PISA) and vena contracta to achieve maximum TR velocity. Obtain the peak velocity from either the A4C, parasternal short-axis or RV inflow view. Optimise the gain and reject settings to obtain a complete envelope with elimination of transit-time artefact. If in AF, averaging over five to ten consecutive beats can be performed. However, where possible, a single measure can be made if specific criteria are met - when the preceding and pre-preceding RR intervals are within 60 ms of each other and both exceed 500 ms, measures of a single beat are similar to those averaged over 15 cycles of varying durations (107). Although there is little data available for the assessment of TR velocity in AF, these findings suggest that selection of beats with similar RR intervals is more important for reproducibility than the total number of measures made and should be considered when estimating SPAP. | 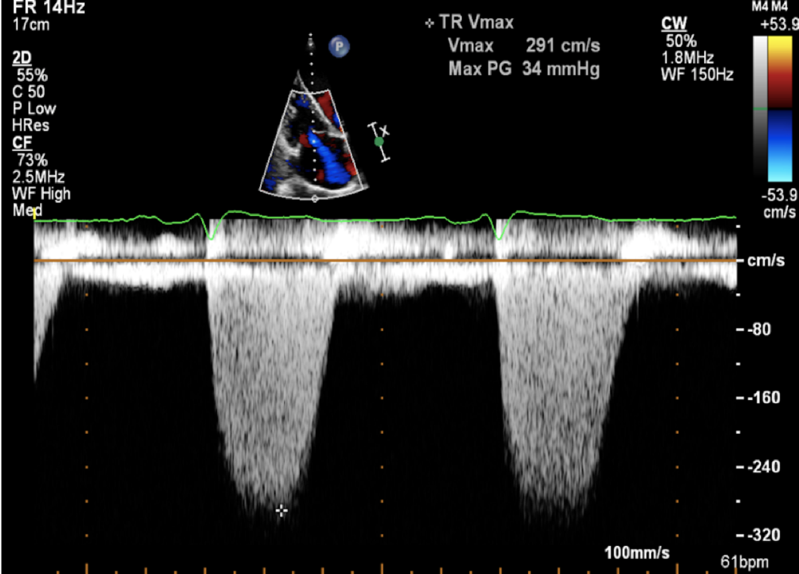 |
| **LA strain (reservoir and pump)** | |
| The method for measuring left atrial strain varies according to vendor and is semi-automated on most platforms. When acquiring images for LA strain analysis, dedicated atrial windows should be acquired to maximum LA volume. LA strain analysis performed in views optimised for the LV and therefore foreshortening the LA leads to overestimation of LA strain values. Zooming on the LA is likely to improve image accuracy. Although both monoplane and biplane LA strain analysis methods are available, there is no significant difference in calculated values between the two methods. Red trace – reservoir phase, blue trace – conduit phase, green trace – pump phase. | 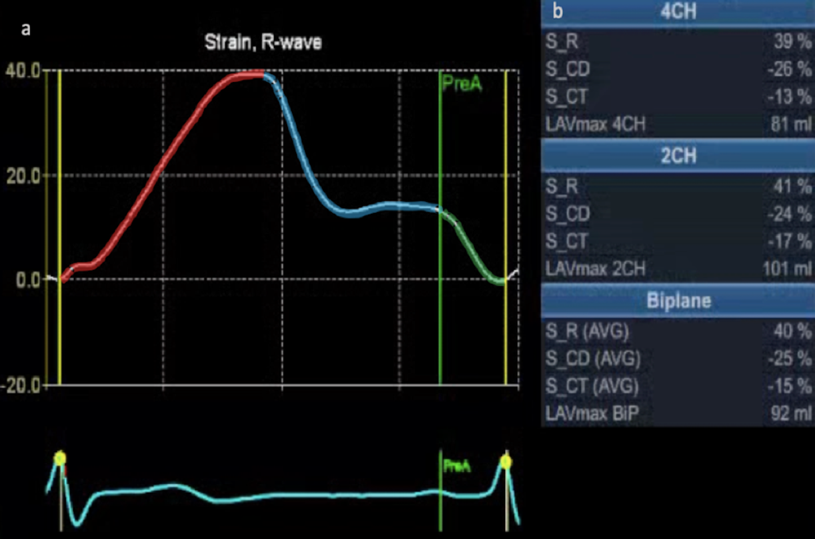 |
